# Introduction to Treating Patients Exposed to Chemical, Biological, Radiological, and Nuclear (CBRN) Threats: A Military Medical Case-Based Curriculum

**DOI:** 10.15766/mep_2374-8265.11433

**Published:** 2024-09-13

**Authors:** Alice Sardarian, Melissa Givens, James F. Schwartz, Rebekah Cole, Sherri L. Rudinsky

**Affiliations:** 1 Fourth-Year Medical Student, Uniformed Services University of the Health Sciences F. Edward Hébert School of Medicine; 2 Professor and Vice Chair, Department of Military and Emergency Medicine, Uniformed Services University of the Health Sciences F. Edward Hébert School of Medicine; 3 Assistant Professor, Department of Military and Emergency Medicine, Uniformed Services University of the Health Sciences F. Edward Hébert School of Medicine; 4 Research Associate Professor, Department of Military and Emergency Medicine, Uniformed Services University of the Health Sciences F. Edward Hébert School of Medicine; 5 Associate Professor and Chair, Department of Military and Emergency Medicine, Uniformed Services University of the Health Sciences F. Edward Hébert School of Medicine

**Keywords:** Emergency Medicine, Medical Toxicology, Case-Based Learning, Editor's Choice

## Abstract

**Introduction:**

Ensuring proficiency in responding to, evaluating, and treating chemical, biological, radiological, and nuclear (CBRN) casualties is a critical component of military medical student education. To meet this objective, we developed a case-based CBRN curriculum that can serve as a model to address potential curricular gaps for civilian prehospital, UME, and GME programs.

**Methods:**

The curriculum was administered in two sessions, 1 month apart, each with individual student preparation, including an optional asynchronous online module and a review of clinical practice guidelines. Session one consisted of a 2-hour introductory lecture, followed by a student reflection. Session two consisted of a 1-hour small-group case study, designed as a multimodal exercise with a corresponding computer-based worksheet and knowledge check.

**Results:**

Forty-five teams consisting of three to four second-year medical students (*N* = 170) completed the sessions and course survey. Sixty-four percent of student teams were extremely or quite satisfied with what they learned, 62% found the materials very or quite relevant to their needs, and 69% rated the instructional materials as extremely or quite understandable. Student feedback included designating additional time for worksheet completion.

**Discussion:**

A case-based training on CBRN patient care earned positive ratings for the clarity of instruction, the impact on students as learners, and the feasibility of the training. Future training evolutions could track student completion of prework, extend the allotted time for activity completion, and evaluate curricular effectiveness through pre-post measurement of students’ confidence in their ability to care for a CBRN patient.

## Educational Objectives

By the end of this activity, learners will be able to:

1. Describe the potential for chemical, biological, radiological, and nuclear (CBRN) injuries and where to locate curated resources.
2. Implement the CRESS (consciousness, respirations, eyes, secretions, skin) algorithm to rapidly evaluate for a potential chemical agent exposure.
3. Develop an organized approach to the evaluation and treatment of a CBRN patient using principles of (MARCHE)2 (Massive hemorrhage, Mask, Airway, Antidotes, Respirations, Rapid spot decontamination, Circulation, Countermeasures, Hypothermia, Head wounds, Evacuation, Extraction).
4. Differentiate the presenting signs and symptoms of nerve and pulmonary agents.
5. Describe the mechanism of action for nerve agents and how this informs treatment of these patients.

## Introduction

Chemical, biological, radiological, and nuclear (CBRN) threats continue to evolve and escalate in severity and frequency. They may involve the accidental or intentional deployment of weapons or hazardous substances, which can lead to local or global catastrophe. Modern CBRN events include the 2001 anthrax spore mailings, which resulted in five deaths; the widespread use of chemical weapons in the ongoing Syrian conflict, resulting in at least 1,200 recorded deaths between March 2011 and April 2017; and the poisoning of Sergei and Yulia Skripal by Novichok nerve agents in 2018.^1–3^ The COVID-19 pandemic has proven that biological threats can have significant implications for national security.^4^ Meanwhile, the recent incidents at nuclear facilities in Ukraine have highlighted the potential for nuclear disaster.^5^ The Department of Defense has developed service-specific publications and guidance on addressing CBRN patients while maintaining a robust health systems support.^6^ Contaminated casualty care is highlighted as an essential element of military medical preparedness, thus demanding its integration into military medical students’ curricula.^7^ The critical and emergent management of biological warfare agents, toxic and hazardous chemical exposures, procedural skills of decontamination and antidote administration, and emergency preparedness are all discrete elements within the Model of Clinical Practice in Emergency Medicine.^8^

CBRN and medical toxicology education has typically been limited to residency training in emergency medicine within the United States. This is, however, dependent on the specific residency program. A 2018 survey of residency directors for civilian emergency medicine programs found that 39 of 164 programs had no toxicology rotations available.^9^ To our knowledge, no CBRN teaching materials have been published for medical student education. Thus, there appears to be a gap in training that can be addressed earlier in medical education and across all specialties. An educational summary published in the *Journal of Education & Teaching in Emergency Medicine,* describing a blended asynchronous-synchronous toxicology curriculum for emergency medicine residents, found that this multimodal platform had a favorable response amongst students.^10^ A literature review from 2018 assessing CBRN-related training concluded that scenario-based training is the most effective educational strategy but that training opportunities are limited worldwide.^11^

Using the above concept of a blended curriculum based on a scenario and following the Defense Health Agency's Joint Trauma System clinical practice guidelines for CBRN injury, which integrate tactical combat casualty care (TCCC; prehospital trauma care) and CBRN, we developed a case-based learning curriculum appropriate for the medical student level of training.^12–14^ The curriculum is grounded in constructivist learning theory, which expands upon students’ preexisting knowledge and emphasizes team problem-solving through critical thinking and deductive reasoning within a real-world scenario.^15^ While we have tailored our curriculum to the standards of care determined by the Defense Health Agency for the Military Health System, we believe the framework can be broadly applied to civilian prehospital, graduate, and undergraduate medical education programs given the vast potential for CBRN threats in the civilian environment.

The case-based, multimodal curriculum described here was developed to address the following general topics: recognizing symptoms of CBRN exposure, prioritizing patient and provider safety, understanding the steps of medical management depending on the offending agent, and gaining familiarity with the clinical practice guidelines utilized in the military health system. It is tailored towards our preclerkship medical students, who have some or little prior knowledge of CBRN patient care.

## Methods

At the Uniformed Services University of the Health Sciences (USUHS), undergraduate medical education consists of 1.5 years of preclerkship study, followed by 2.5 years of core clerkships and advanced clinical rotations. The Military and Emergency Medicine Department provides a longitudinal curriculum across all 4 years that incorporates hands-on skills sessions, lectures, and case-based activities in the classroom, as well as larger multiday operational field training exercises like Operation Bushmaster.^16^ This CBRN curriculum was provided to second-year medical students towards the end of their preclerkship period, and its material will be revisited during a hands-on CBRN event at a large-scale immersive simulation exercise in their fourth year. The curriculum was delivered in November and December 2021 to approximately 170 medical students, organized into 45 preestablished teams. Participation was mandatory for all students in the second-year medical student cohort and included completion of every formative assessment within the exercise and the postexercise survey.

**Figure 1.**
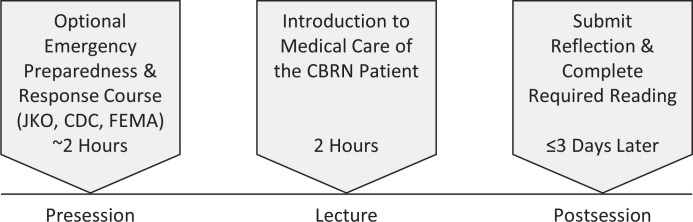
Timeline for session one events. Abbreviations: CBRN, chemical, biological, radiological, and nuclear; FEMA, Federal Emergency Management Agency; JKO, Joint Knowledge Online.

Our curriculum did not require prior knowledge on the topic of CBRN and was designed in two parts to provide foundational knowledge prior to the case-based exercise. We dedicated 2 hours to session one, which took place in November 2021 (Figure 1). Prior to the session, we highly encouraged the students to complete an optional online training module entitled Emergency Preparedness and Response Course. This particular module is available via the Joint Knowledge Online website operated by the Joint Chiefs of Staff as a platform for joint training and education throughout the Department of Defense. Given that military access is required to view this module, we have also cited similar open-access online modules available through the CDC and the Federal Emergency Management Agency that would serve well as introductory courses for civilian learners in place of the Joint Knowledge Online module.^17,18^ Prior to the lecture, we asked students to

look at the material through the lens of a leader, a clinician caring for a single patient or few patients, and as an individual who needs to protect themselves. Try to come up with some questions as you ponder those roles for the discussion at the end of the lecture.

During the allotted session time, students gathered in a lecture hall and as a cohort received a synchronous introductory lecture on medical care of the CBRN patient (Appendix A). The lecture was given by MG, whose expertise lies in emergency medicine, CBRN, and toxicology. Students also had the option to view the synchronous lecture in a virtual format. The lecture slides were available to the students prior to the session. The lecture hall included projector and microphone capabilities, and the lecture was broadcast virtually via Google Meets. This lecture addressed Educational Objectives 1–3, including explaining the North Atlantic Treaty Organization CRESS (consciousness, respirations, eyes, secretions, skin) algorithm and emphasizing the importance of CBRN preparedness amongst military medical leadership.^19^ Following the lecture, we tasked students with reading “Integrating Chemical Biological, Radiologic, and Nuclear (CBRN) Protocols Into TCCC Introduction of a Conceptual Model - TCCC + CBRN = (MARCHE)2.”^14^ Within 3 days of session one, we required the students to submit an individual reflection addressing the following question: “How are the constructs of TCCC and the approach to CBRN similar and dissimilar, and why is it important to have a common framework for these topics?” This reflection was graded for completion on a pass/fail basis. To supplement their studies, we provided additional, optional resources to the students for their review (Appendix B).

**Figure 2.**
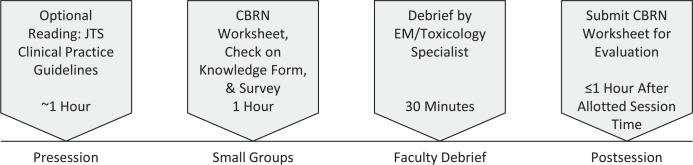
Small-group case-study timeline for session two events. Abbreviations: CBRN, chemical, biological, radiological, and nuclear; EM, emergency medicine; JTS, Joint Trauma System.

Session two was scheduled for 1.5 hours in December 2021, approximately 1 month after session one due to academic calendar constraints (Figure 2). Session two addressed Educational Objectives 3–5 via a CBRN small-group case-study. Prior to the session, we highly encouraged the students to review the Joint Trauma System clinical practice guidelines as optional prereading, again with the instructions to “look at the material through the lens of a leader, a clinician caring for a single patient or few patients, and as an individual who needs to protect themselves.”^12,13^ We split the students into predetermined teams of three to four to complete the case-based worksheet. Students gathered in a large meeting space, with one team per table, in order to complete their activity. We asked them to bring their computers to access and fill in the CBRN patient worksheet (Appendix C) with corresponding, author-owned videos (Appendices D–F), as well as the check on knowledge form (Appendix G). The CBRN patient worksheet included linked videos demonstrating the scenario, which students viewed at various steps throughout the activity (Appendices D–F). Per the worksheet instructions, we tasked each team of students with placing a chemical and biological respirator on one member of their team who acted as an unconscious patient.

We instructed students to complete the CBRN patient worksheet (Appendix C) as a team by generating a copy of the Google document, sharing it with their team members, and renaming the document according to their team. Only one student per team had to open the check on knowledge form (Appendix G), which was embedded within the CBRN patient worksheet and provided immediate feedback throughout the exercise. Three faculty facilitators from the Department of Military and Emergency Medicine were available throughout the case-based activity; while the student activity was largely independent, the facilitators were available to address any questions (Appendices H and I). At the conclusion of the activity, each team completed the survey form (Appendix J), which was attached to the check on knowledge form, with one survey representing each team. We asked that one student per team download their worksheet as a PDF and submit it for a completion grade (pass/fail) on behalf of their team; the submission deadline was 1 hour after the allotted session time. At the end of the session, students received a 30-minute faculty debrief in a lecture hall with microphone capabilities. The debrief addressed student questions and elicited key takeaways and relevance to clinical practice. Additional optional supplemental resources for review before and after the activity were provided as well, just as in session one (Appendix K).

The survey utilized to evaluate student course satisfaction was developed using the Google Forms platform (Appendix J). This was chosen to facilitate integration with the activity's check on knowledge form (also on Google Forms) and to centralize the data collection (Appendix G). In addition to soliciting feedback on a 5-point scale for each question regarding the curriculum design and execution, students were also given a free-response option to note any additional comments. Each point on the scale had appropriate descriptors to differentiate it, with the exception of the question on the students’ assessment of the amount of time they had to complete the lesson, which was a numerical scale only. Submission of the survey form was required for completion of the activity, with the exception of the optional narrative feedback question at the end of the survey.

## Results

Approximately 170 students and three facilitators participated in this two-part CBRN case-based curriculum, with all 45 student teams completing and submitting the survey at the conclusion of session two (100% response rate). The results from the survey are detailed in the Table. A majority of the student teams were satisfied with the clarity of the instruction, with 80% of the teams finding the scenario to be extremely or quite appropriate for the context of the lesson. A majority remained motivated as they progressed through the material, with 62% of the teams finding the materials very or quite relevant to their needs and interests. The format of the training was sufficiently engaging and understandable, and 67% of the teams rated the sequence of activities as seamless or quite connected.

**Table.**
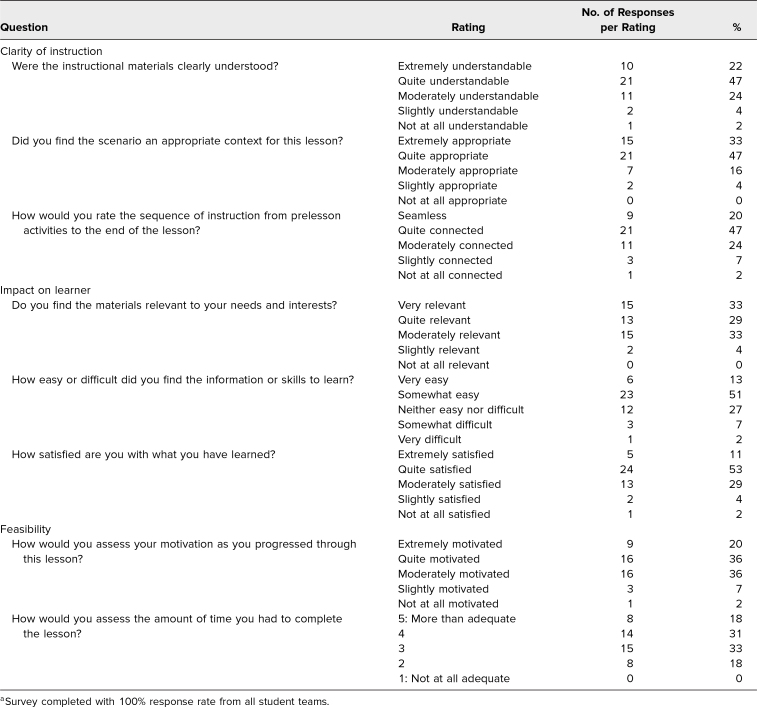
Student Team Ratings on Postactivity Survey (*N* = 45)^a^

Ten teams provided written feedback in the additional comments section of the survey. Two teams suggested scheduling the two CBRN sessions closer in time, or even paired on the same day. One team recommended making the activity entirely virtual, and another noted that they had “felt a little rushed” to complete the exercise. This was in line with student ratings of time to complete the session, with 64% of the student teams deeming the amount of time available to complete the lesson as a 3 or 4 out of 5 total points. The only comment that pertained specifically to modifying the curriculum noted that the patient decontamination site section was “confusing and needs additional instruction.” All other comments indicated that the students “enjoyed [the] session and debrief,” thought the training was “a good team exercise,” and liked the overall “format of these exercises. [They] liked working as a team with on hand props.”

## Discussion

This case-based CBRN training proved to be a well-received mode of teaching CBRN patient care to a medical student cohort. The curriculum was intentionally designed to be multimodal, as opposed to solely virtual, to facilitate an engaging student-driven learning experience. This approach was consistent with existing education literature and was intended to encourage students to work together as teams to simulate a response to CBRN events, which will likely be done by medical military-civilian teams in the future.^10,11^

There are, however, several points of improvement, as highlighted by some of the student teams. There was 1 month between the sessions, owing primarily to limitations in the students’ academic calendars. This lapse in time may have been beneficial, in that students needed to spend more time reviewing what they had forgotten, relating to the evidence-based learning strategy of spaced retrieval.^20^ In future iterations, if there is time and availability in student schedules, it may be possible to condense the sessions to 1 day with a scheduled break between them; this would require moving the reflection and reading assignments for session one to the conclusion of both sessions. A week between each session may allow time for students to review the content, while also being close enough that they feel more comfortable with applying their knowledge to the case study.

One team had difficulty with the patient decontamination site section, which involved determining an appropriate casualty drop-off point on a map (Appendices C and H). An optional supplemental resource detailing an example of a casualty decontamination site and a proposed patient flow depending on the wind direction, patient stability, and so on was linked within the activity. The student team asked for additional clarifying instructions for this section. While we assumed that students would refer to the supplemental resource, it may be more helpful to encourage them to open the resource and specifically refer to the decontamination site layout prior to answering the question.

Additionally, some students felt they had limited time to complete the exercise. On a scale of 1–5, a majority of students assessed the time allotted for the lesson as being a 3 or 4, suggesting a need for additional time added to session two. We will consider modifying the schedule to accommodate an additional 15–30 minutes for the case-based activity, so that students can complete the exercise and the survey while still having time to practice with the chemical and biological respirator. We have included the recommended time for this activity in the facilitator guide (Appendix I).

A potential limitation of this curriculum review was the format of the Google Forms completed by the student teams; the use of a digital worksheet with prerecorded videos and a knowledge check form with immediate feedback ensured a standardized approach to achieving the Educational Objectives. Attaching the feedback form to the end of the knowledge check helped streamline the process and ensure that the feedback was completed (since the feedback survey was mandatory). At the same time, this limited our ability to assess individual learners’ reactions to the curriculum, since they completed the feedback survey as teams. It is possible that a single student completed the feedback without asking for team input or that a few members of the team completed it without consulting the others, leading to skewed results that may not be representative of all students. In the future, this can be resolved by developing a separate feedback form to be completed by each individual student; in order to ensure form submission while ensuring anonymity, the feedback survey can also be utilized to track attendance.

An additional limitation was our inability to track completion of prereading and other prework, as well as their overall impact on student performance and readiness for the case-based worksheet. We also did not track whether in-person student attendance at the session one lecture facilitated enhanced student understanding of CBRN patient assessment and management when compared with students who attended the lecture remotely. We encourage future facilitators to track these metrics for their own program evaluation needs.

The general teaching framework implemented by the USUHS Military and Emergency Medicine Department trains students to be able to operate proficiently in high-fidelity, simulated medical exercises involving complex casualty care. These exercises progressively increase in complexity and difficulty, testing medical and leadership skills in simulated austere environments.^21^ Our introductory CBRN training can be augmented by hands-on skills training that occurs at these larger field exercises, with the goal of making the training as realistic as possible. In the future, it may be beneficial to assess students’ confidence in their skills before and after the case-based training, such as via OSCEs.^22^ This could be a more direct measure of the effectiveness of the training. Students could then be followed to their fourth-year field exercise at Operation Bushmaster to reassess their confidence in their skills and to obtain more objective measures, such as their ability to perform CBRN patient care completely and accurately.

We also find there to be great potential for collaboration in sharing military medical curricula with civilian institutions. The benefits of military-civilian medical training collaboration and all-hazards response have been well documented in the literature and highlighted during the recent COVID-19 pandemic response.^23–27^ For example, an interoperability study on the military-civilian National Disaster Medical System (NDMS) was conducted by Kirsch and colleagues between 2020 and 2022.^26,27^ The NDMS was established in 1984 to facilitate a national health care response to large-scale disasters or military conflicts. The authors identified several areas for improvement, including the lack of a “federal management plan to coordinate care across the federal and private sector.”^27^ Most respondents supported or strongly supported conducting “federal–private sector combat medical surge planning” and “federal–private sector training, education, and exercises for combat medical surge,” along with the development of “just-in-time training” for civilian and military partners.^27^

We believe there is significant value in beginning student familiarization with CBRN threats and patients in the preclerkship period, in advance of residency training, especially since not all programs offer formal CBRN/toxicology curricula. In both military and civilian contexts, it is essential to train a variety of personnel to facilitate a coordinated response in the event of a CBRN threat. Our curriculum provides one small example of how curricular development across the civilian-military spectrum can be accomplished. While this curriculum was designed for military undergraduate medical education and for use in operational environments, we believe it can be adapted to additional audiences. We have included examples of how to adapt it for emergency medical services personnel in the facilitator guide (Appendix I) but would recommend that facilitators reference their local health department, emergency medical services, hospital, and regional poison control guidelines.

When completing this exercise with the next class of students, we plan to respond to student feedback by scheduling the sessions closer in time, tracking student completion of optional prework, allotting additional time to the exercise, and soliciting individual as opposed to team feedback. The integration of a pre-post skills assessment and further evaluation at simulated medical exercises may better assess the effectiveness of this curriculum. Through this case-based training, we hope that medical students will have the tools and the general understanding necessary to organize training events for their future medical and support teams or, at the very least, be familiar with the resources they can use to further their knowledge and readiness as medical leaders. We also hope to further the collaboration between the military's medical school and its civilian partners.

## Appendices


Session One Lecture.pptxSupplemental Resources for Session One.docxCBRN Patient Worksheet.docxPatient Worksheet Video - Introduction to CBRN Patient.mp4Patient Worksheet Video - CBRN Corpsman Response.mp4Patient Worksheet Video - Physician Assessment.mp4Check on Knowledge Form.docxCBRN Patient Worksheet - Facilitator Version.docxFacilitator Guide.docxStudent Survey.docxSupplemental Resources for Session Two.docx
*All appendices are peer reviewed as integral parts of the Original Publication.*
