# Supplementary material for: Introduction to Treating Patients Exposed to Chemical, Biological, Radiological, and Nuclear (CBRN) Threats: A Military Medical Case-Based Curriculum
Source: MedEdPORTAL. 2024 Sep 13;20:11433. doi: 10.15766/mep_2374-8265.11433 (PMC11393073; doi:10.15766/mep_2374-8265.11433)
Supplement: Supplementary file 1 — Session One Lecture.pptxSupplemental Resources for Session One.docxCBRN Patient Worksheet.docxPatient Worksheet Video - Introduction to CBRN Patient.mp4Patient Worksheet Video - CBRN Corpsman Response.mp4Patient Worksheet Video - Physician Assessment.mp4Check on Knowledge Form.docxCBRN Patient Worksheet - Facilitator Version.docxFacilitator Guide.docxStudent Survey.docxSupplemental Resources for Session Two.docx [file mep_2374-8265.11433-s001.zip › A. Session One Lecture.pptx]

## Slide 1
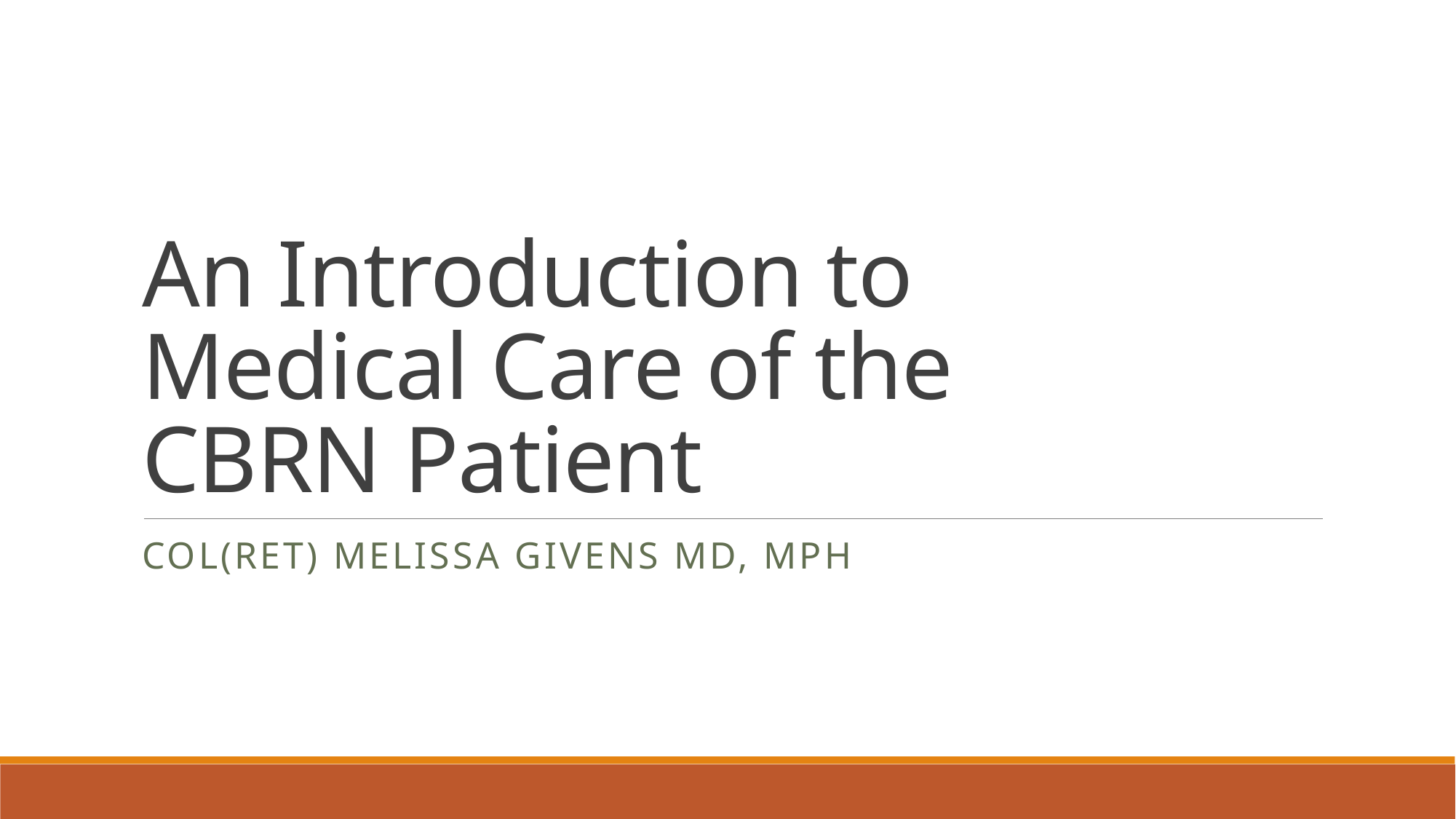

# An Introduction to Medical Care of the CBRN Patient
COL(RET) Melissa Givens MD, MPH

## Slide 2
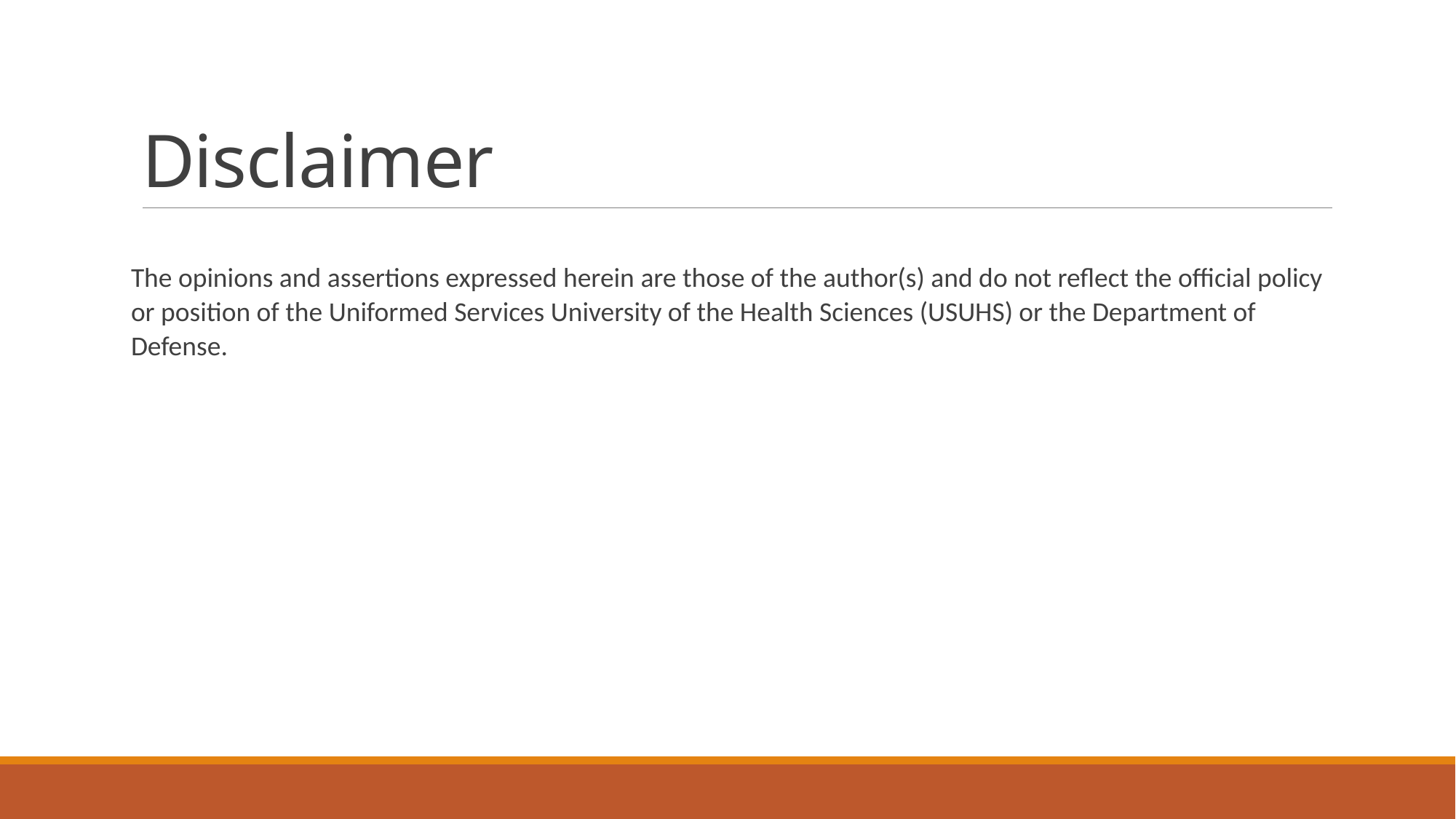

# Disclaimer
The opinions and assertions expressed herein are those of the author(s) and do not reflect the official policy or position of the Uniformed Services University of the Health Sciences (USUHS) or the Department of Defense.

## Slide 3
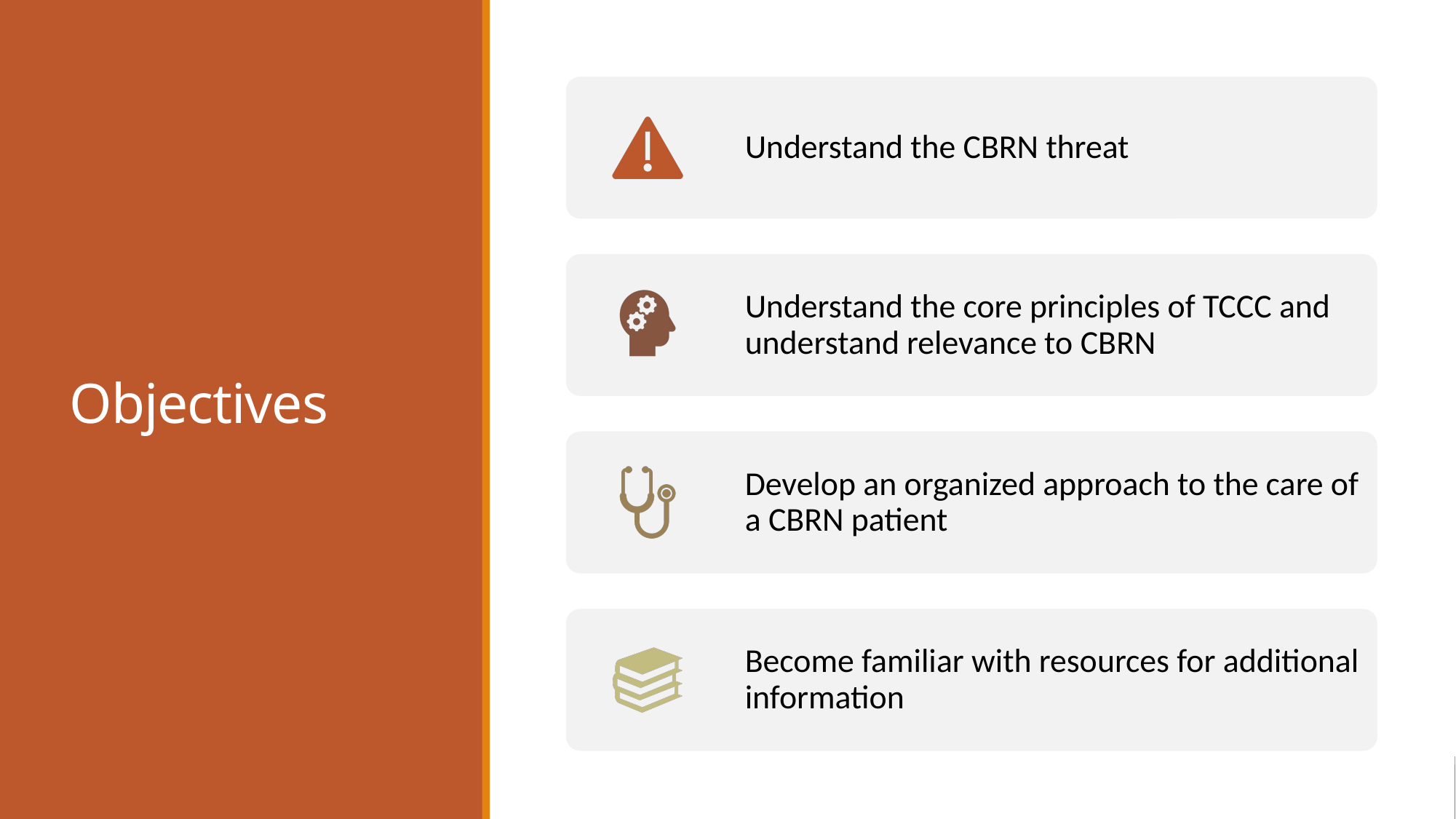

# Objectives

## Slide 4
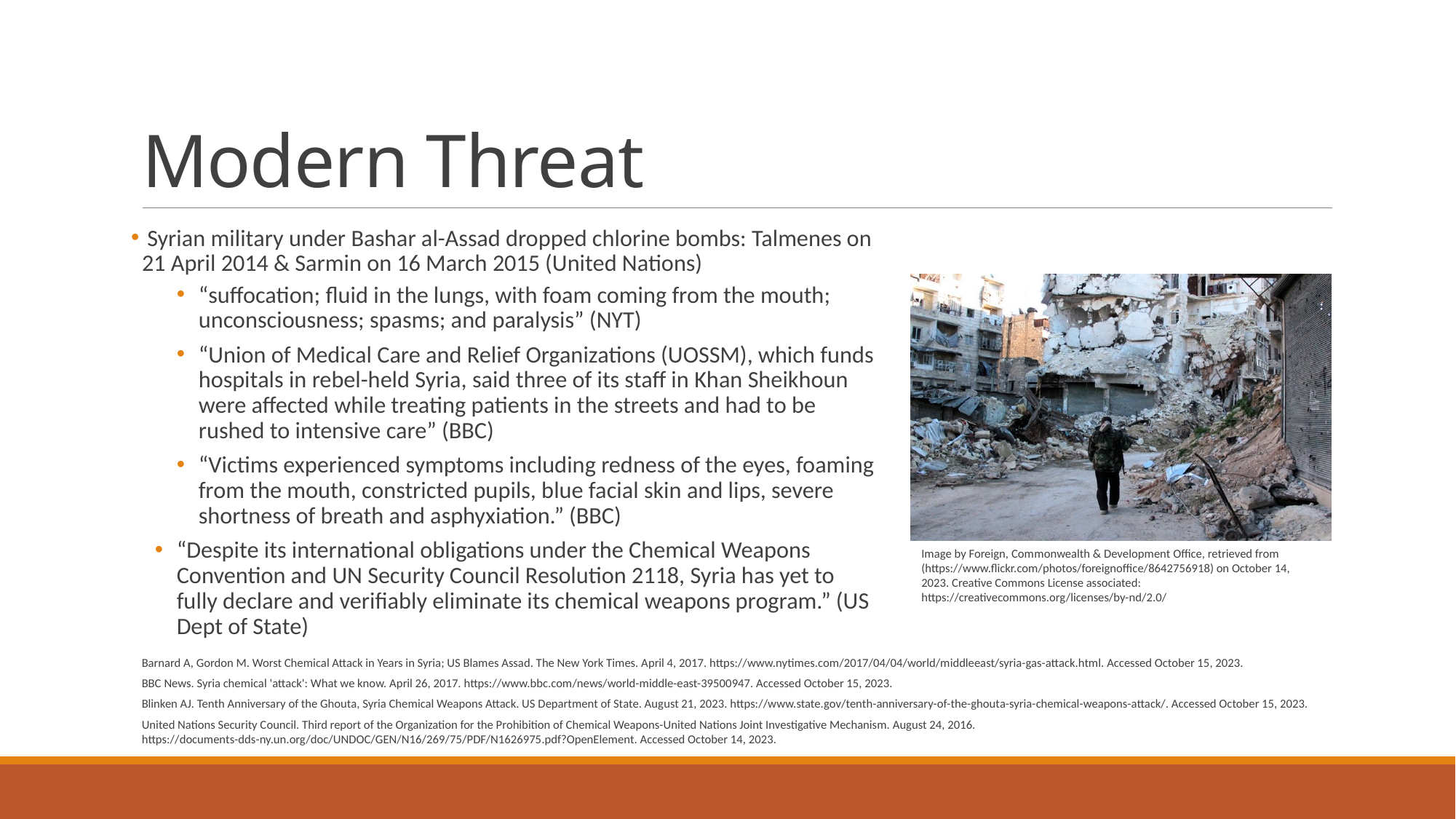

# Modern Threat
 Syrian military under Bashar al-Assad dropped chlorine bombs: Talmenes on 21 April 2014 & Sarmin on 16 March 2015 (United Nations)
“suffocation; fluid in the lungs, with foam coming from the mouth; unconsciousness; spasms; and paralysis” (NYT)
“Union of Medical Care and Relief Organizations (UOSSM), which funds hospitals in rebel-held Syria, said three of its staff in Khan Sheikhoun were affected while treating patients in the streets and had to be rushed to intensive care” (BBC)
“Victims experienced symptoms including redness of the eyes, foaming from the mouth, constricted pupils, blue facial skin and lips, severe shortness of breath and asphyxiation.” (BBC)
“Despite its international obligations under the Chemical Weapons Convention and UN Security Council Resolution 2118, Syria has yet to fully declare and verifiably eliminate its chemical weapons program.” (US Dept of State)
Image by Foreign, Commonwealth & Development Office, retrieved from (https://www.flickr.com/photos/foreignoffice/8642756918) on October 14, 2023. Creative Commons License associated: https://creativecommons.org/licenses/by-nd/2.0/
Barnard A, Gordon M. Worst Chemical Attack in Years in Syria; US Blames Assad. The New York Times. April 4, 2017. https://www.nytimes.com/2017/04/04/world/middleeast/syria-gas-attack.html. Accessed October 15, 2023.
BBC News. Syria chemical 'attack': What we know. April 26, 2017. https://www.bbc.com/news/world-middle-east-39500947. Accessed October 15, 2023.
Blinken AJ. Tenth Anniversary of the Ghouta, Syria Chemical Weapons Attack. US Department of State. August 21, 2023. https://www.state.gov/tenth-anniversary-of-the-ghouta-syria-chemical-weapons-attack/. Accessed October 15, 2023.
United Nations Security Council. Third report of the Organization for the Prohibition of Chemical Weapons-United Nations Joint Investigative Mechanism. August 24, 2016. https://documents-dds-ny.un.org/doc/UNDOC/GEN/N16/269/75/PDF/N1626975.pdf?OpenElement. Accessed October 14, 2023.

## Slide 5
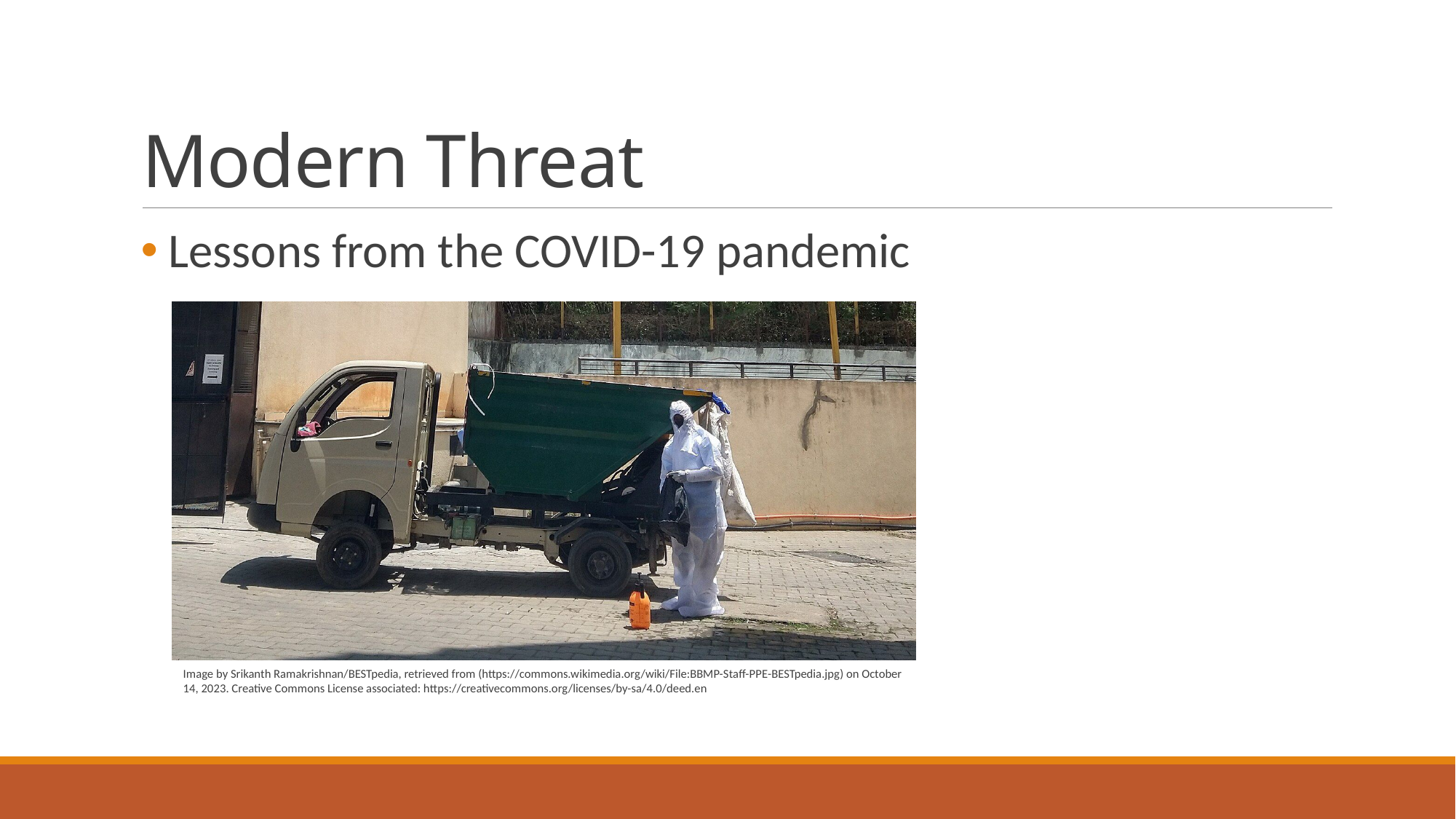

# Modern Threat
 Lessons from the COVID-19 pandemic
Image by Srikanth Ramakrishnan/BESTpedia, retrieved from (https://commons.wikimedia.org/wiki/File:BBMP-Staff-PPE-BESTpedia.jpg) on October 14, 2023. Creative Commons License associated: https://creativecommons.org/licenses/by-sa/4.0/deed.en

## Slide 6
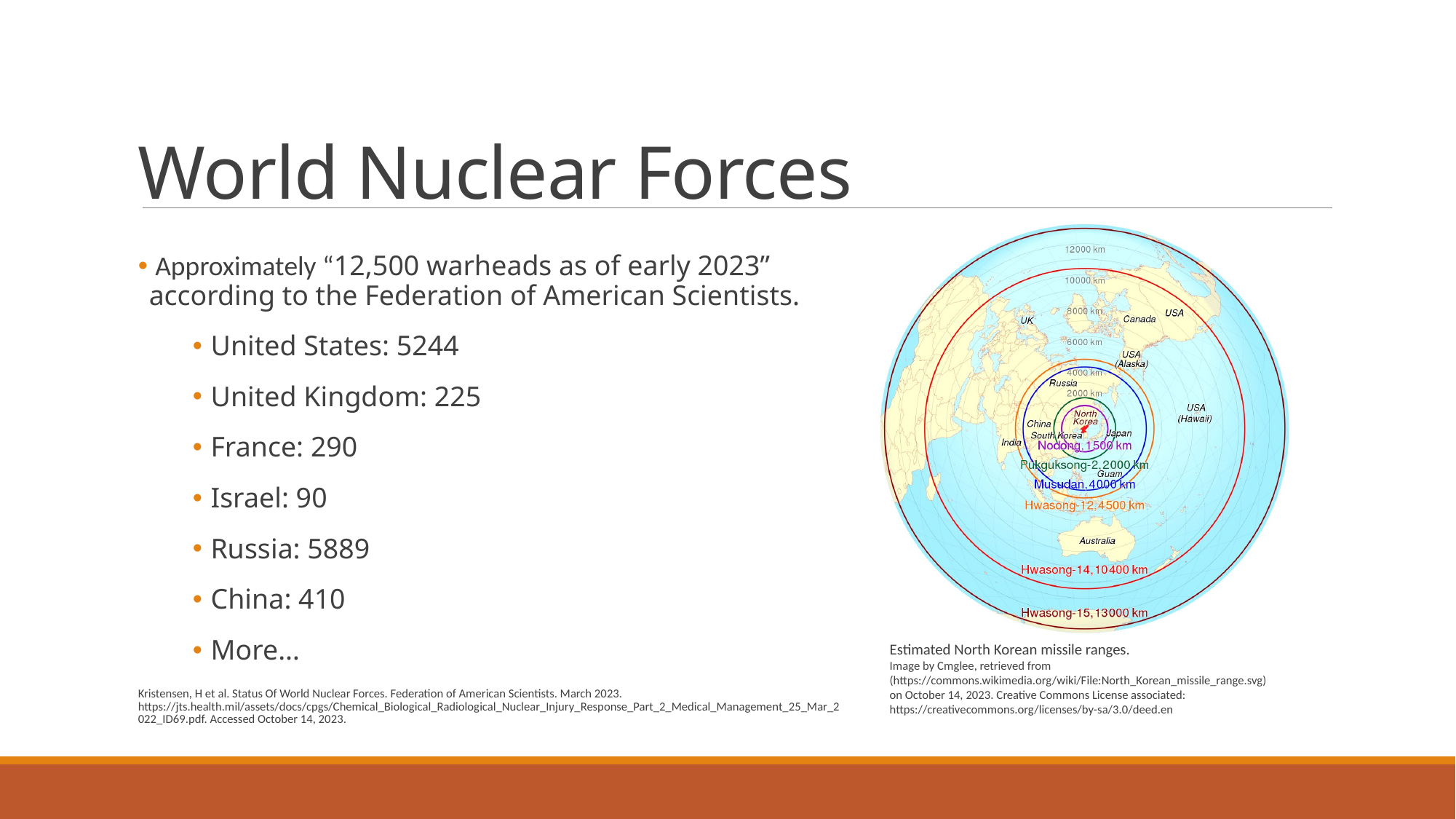

# World Nuclear Forces
 Approximately “12,500 warheads as of early 2023” according to the Federation of American Scientists.
 United States: 5244
 United Kingdom: 225
 France: 290
 Israel: 90
 Russia: 5889
 China: 410
 More…
Kristensen, H et al. Status Of World Nuclear Forces. Federation of American Scientists. March 2023. https://jts.health.mil/assets/docs/cpgs/Chemical_Biological_Radiological_Nuclear_Injury_Response_Part_2_Medical_Management_25_Mar_2022_ID69.pdf. Accessed October 14, 2023.
Estimated North Korean missile ranges.
Image by Cmglee, retrieved from (https://commons.wikimedia.org/wiki/File:North_Korean_missile_range.svg) on October 14, 2023. Creative Commons License associated: https://creativecommons.org/licenses/by-sa/3.0/deed.en

## Slide 7
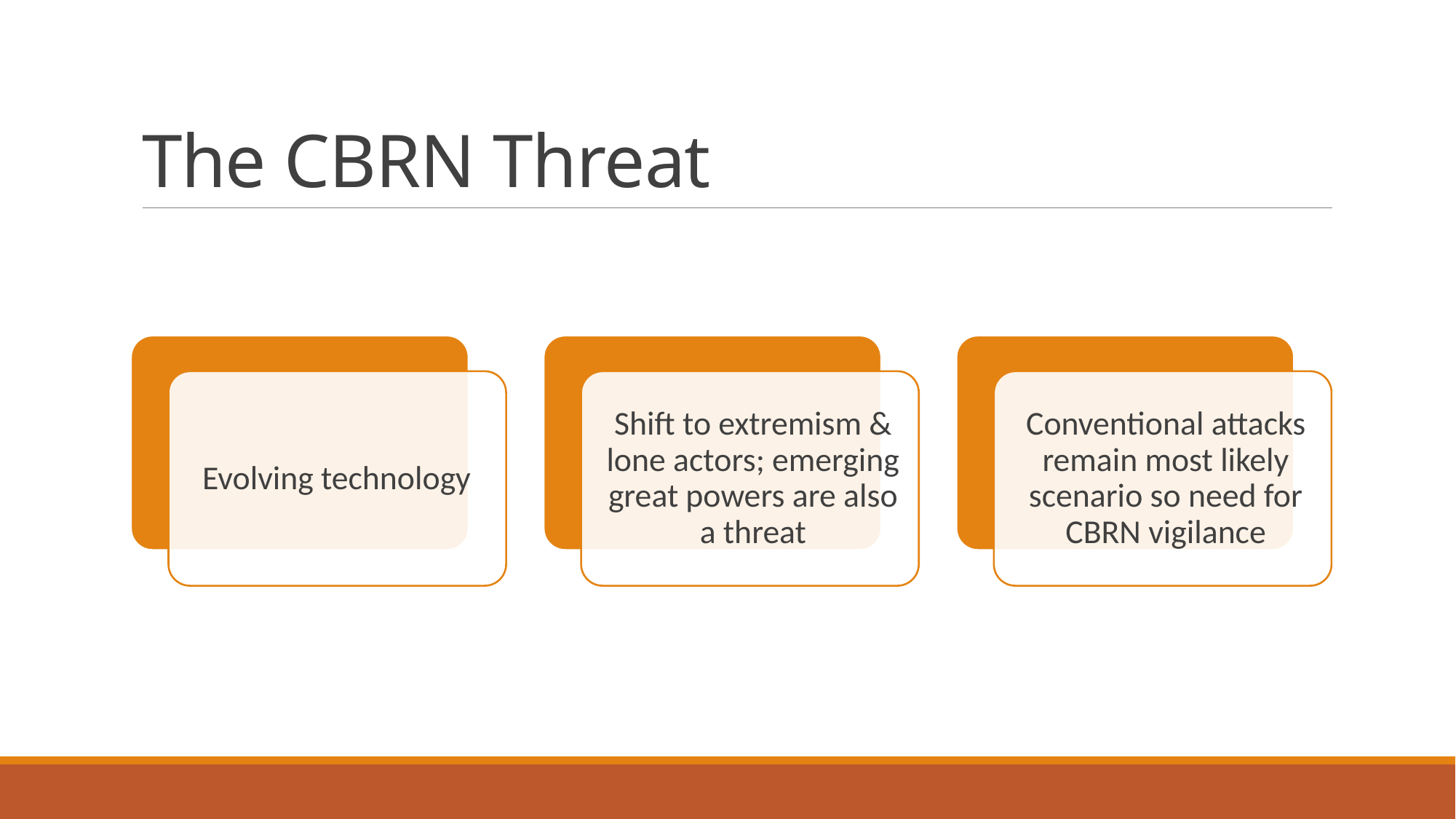

# The CBRN Threat

## Slide 8
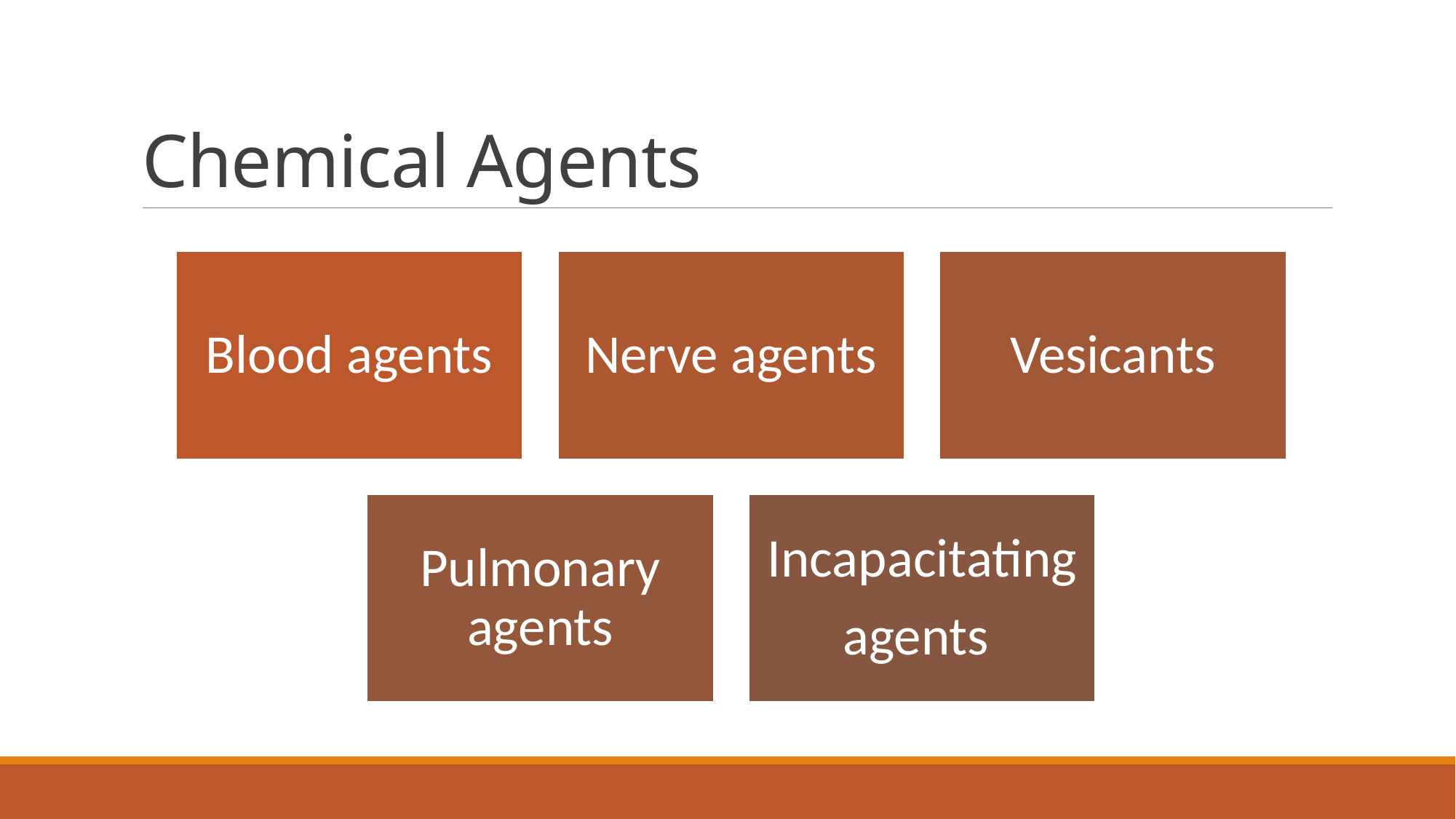

# Chemical Agents

## Slide 9
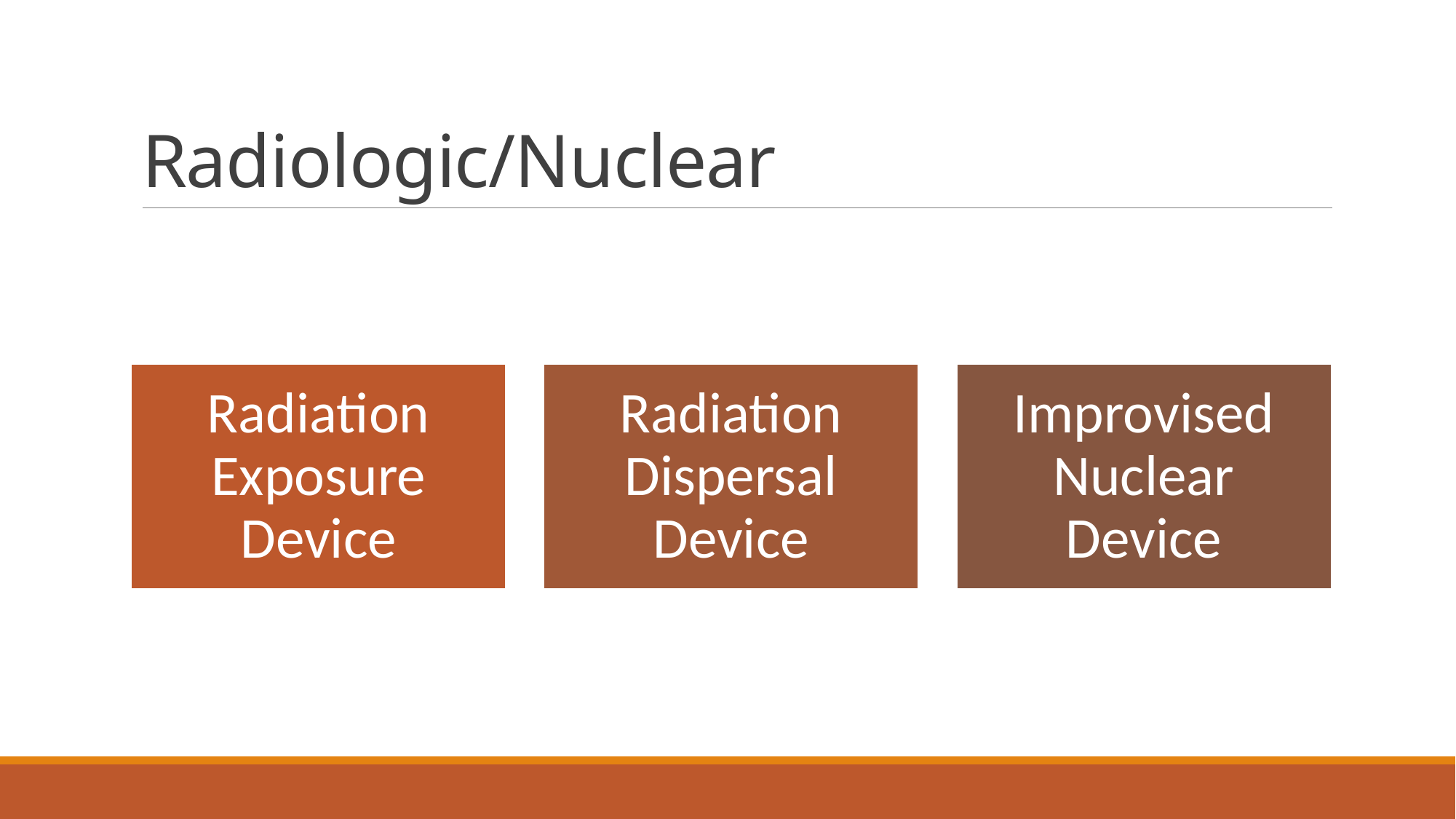

# Radiologic/Nuclear

## Slide 10
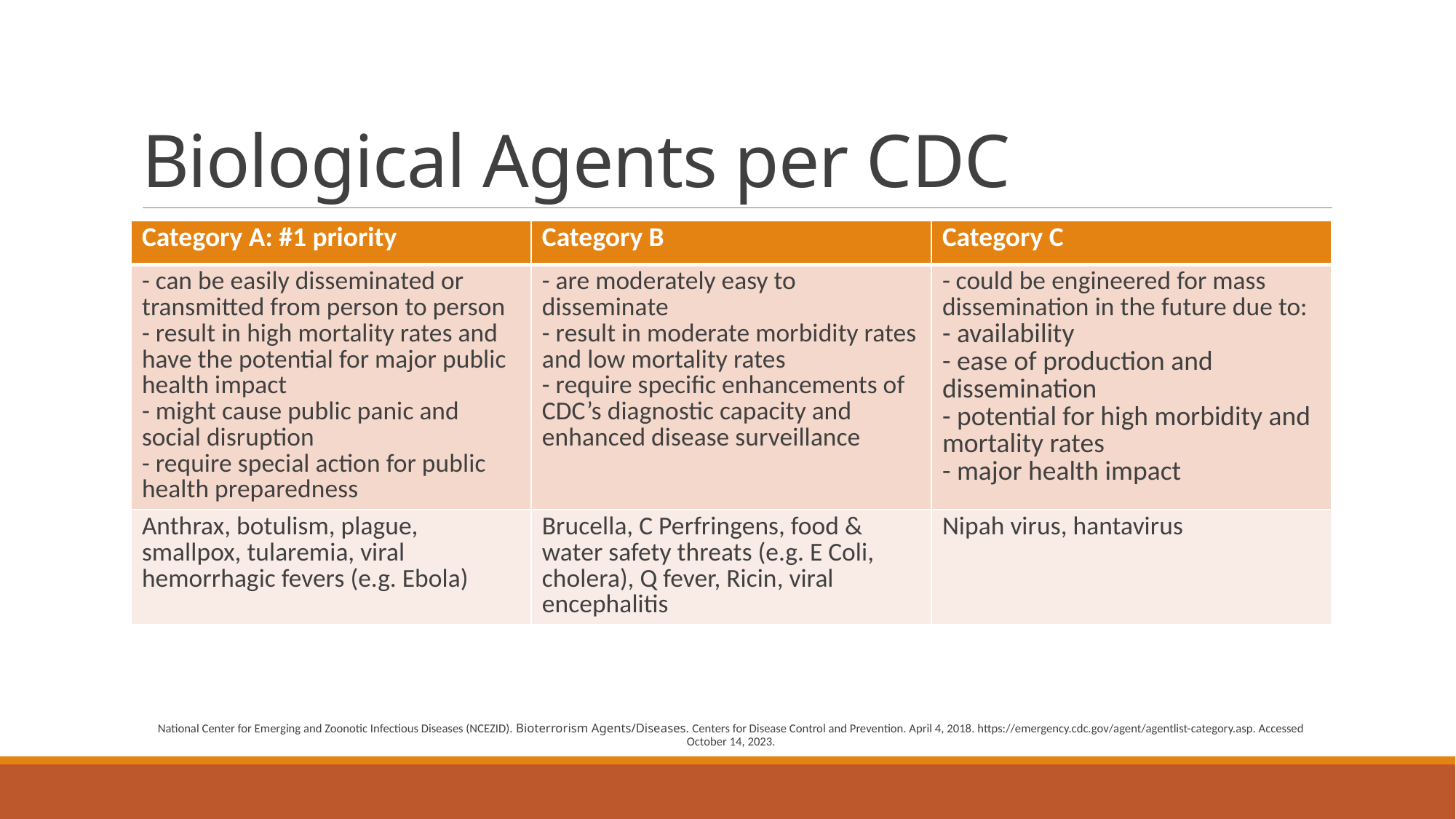

# Biological Agents per CDC
| Category A: #1 priority | Category B | Category C |
| --- | --- | --- |
| - can be easily disseminated or transmitted from person to person - result in high mortality rates and have the potential for major public health impact - might cause public panic and social disruption - require special action for public health preparedness | - are moderately easy to disseminate - result in moderate morbidity rates and low mortality rates - require specific enhancements of CDC’s diagnostic capacity and enhanced disease surveillance | - could be engineered for mass dissemination in the future due to: - availability - ease of production and dissemination - potential for high morbidity and mortality rates - major health impact |
| Anthrax, botulism, plague, smallpox, tularemia, viral hemorrhagic fevers (e.g. Ebola) | Brucella, C Perfringens, food & water safety threats (e.g. E Coli, cholera), Q fever, Ricin, viral encephalitis | Nipah virus, hantavirus |
National Center for Emerging and Zoonotic Infectious Diseases (NCEZID). Bioterrorism Agents/Diseases. Centers for Disease Control and Prevention. April 4, 2018. https://emergency.cdc.gov/agent/agentlist-category.asp. Accessed October 14, 2023.

## Slide 11
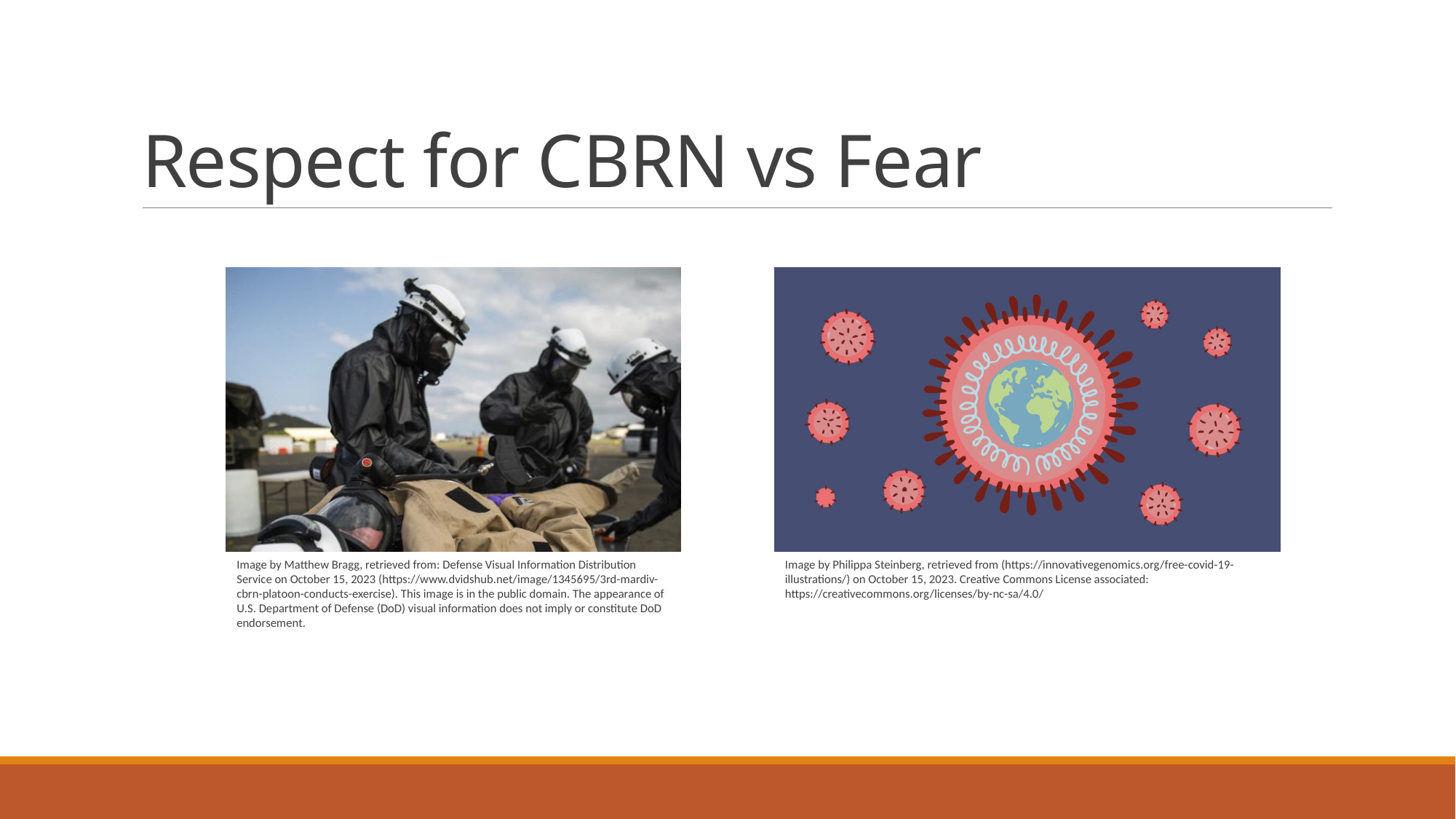

# Respect for CBRN vs Fear
Image by Matthew Bragg, retrieved from: Defense Visual Information Distribution Service on October 15, 2023 (https://www.dvidshub.net/image/1345695/3rd-mardiv-cbrn-platoon-conducts-exercise). This image is in the public domain. The appearance of U.S. Department of Defense (DoD) visual information does not imply or constitute DoD endorsement.
Image by Philippa Steinberg, retrieved from (https://innovativegenomics.org/free-covid-19-illustrations/) on October 15, 2023. Creative Commons License associated: https://creativecommons.org/licenses/by-nc-sa/4.0/

## Slide 12
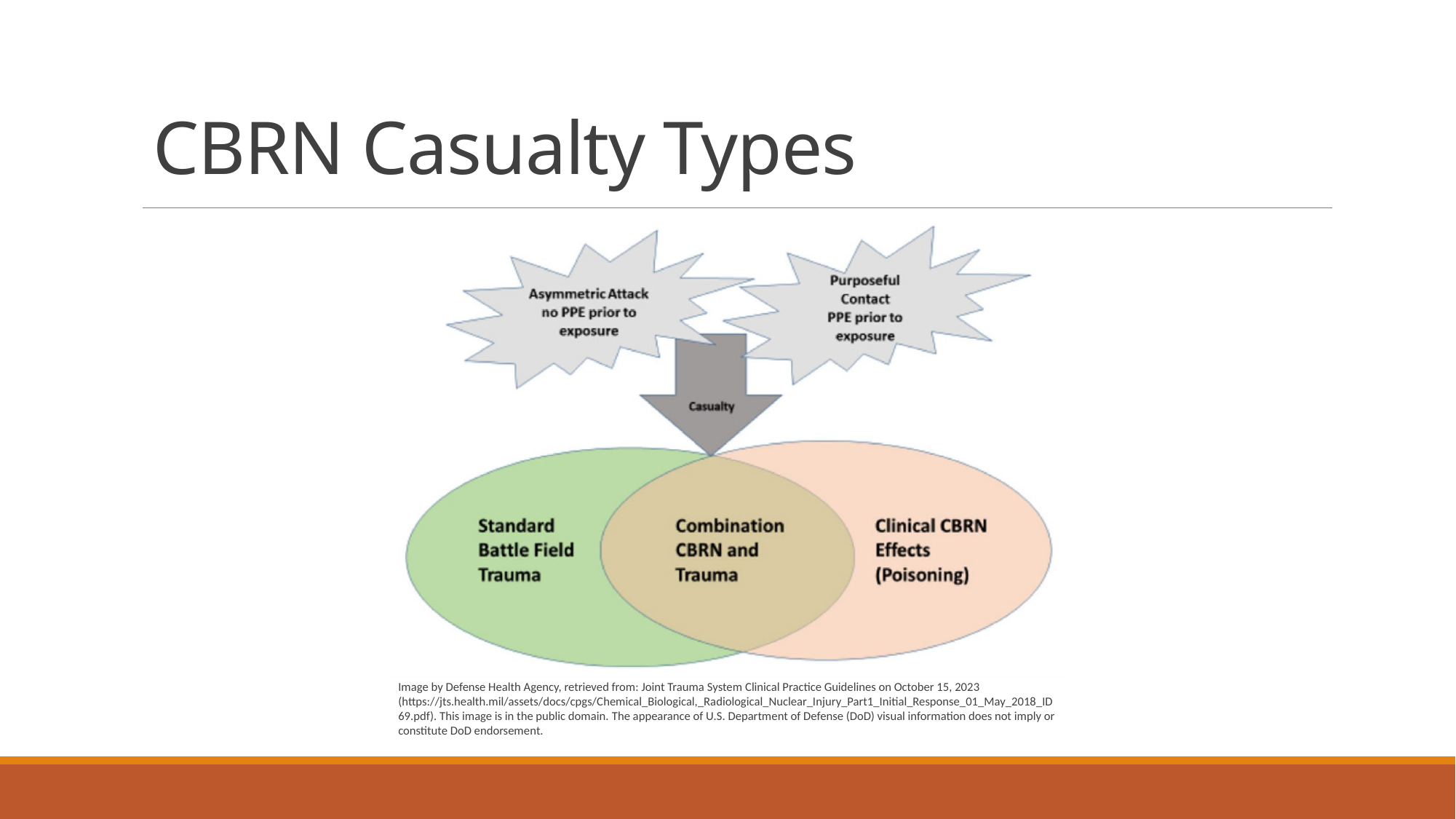

# CBRN Casualty Types
Image by Defense Health Agency, retrieved from: Joint Trauma System Clinical Practice Guidelines on October 15, 2023 (https://jts.health.mil/assets/docs/cpgs/Chemical_Biological,_Radiological_Nuclear_Injury_Part1_Initial_Response_01_May_2018_ID69.pdf). This image is in the public domain. The appearance of U.S. Department of Defense (DoD) visual information does not imply or constitute DoD endorsement.

## Slide 13
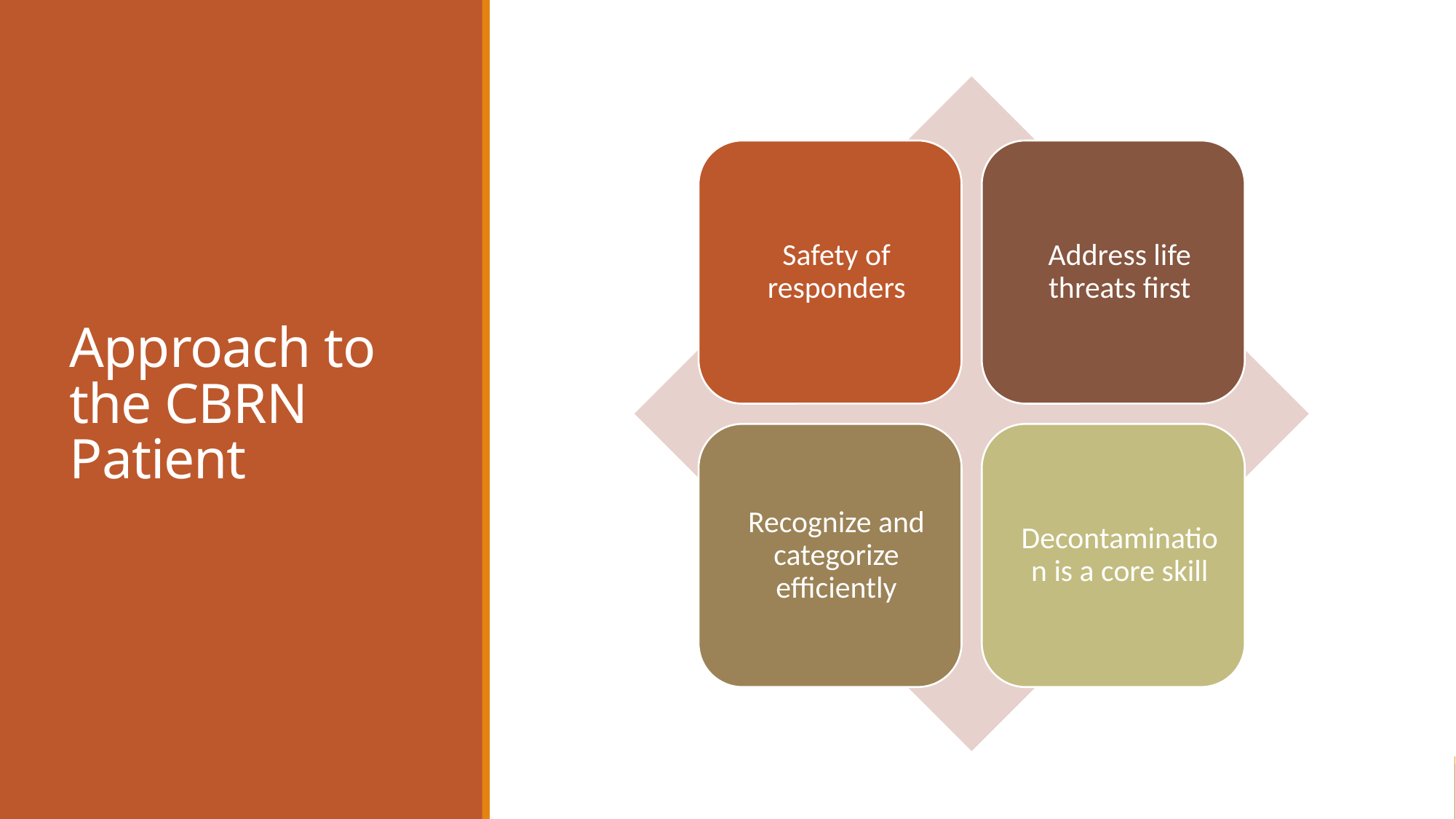

# Approach to the CBRN Patient

## Slide 14
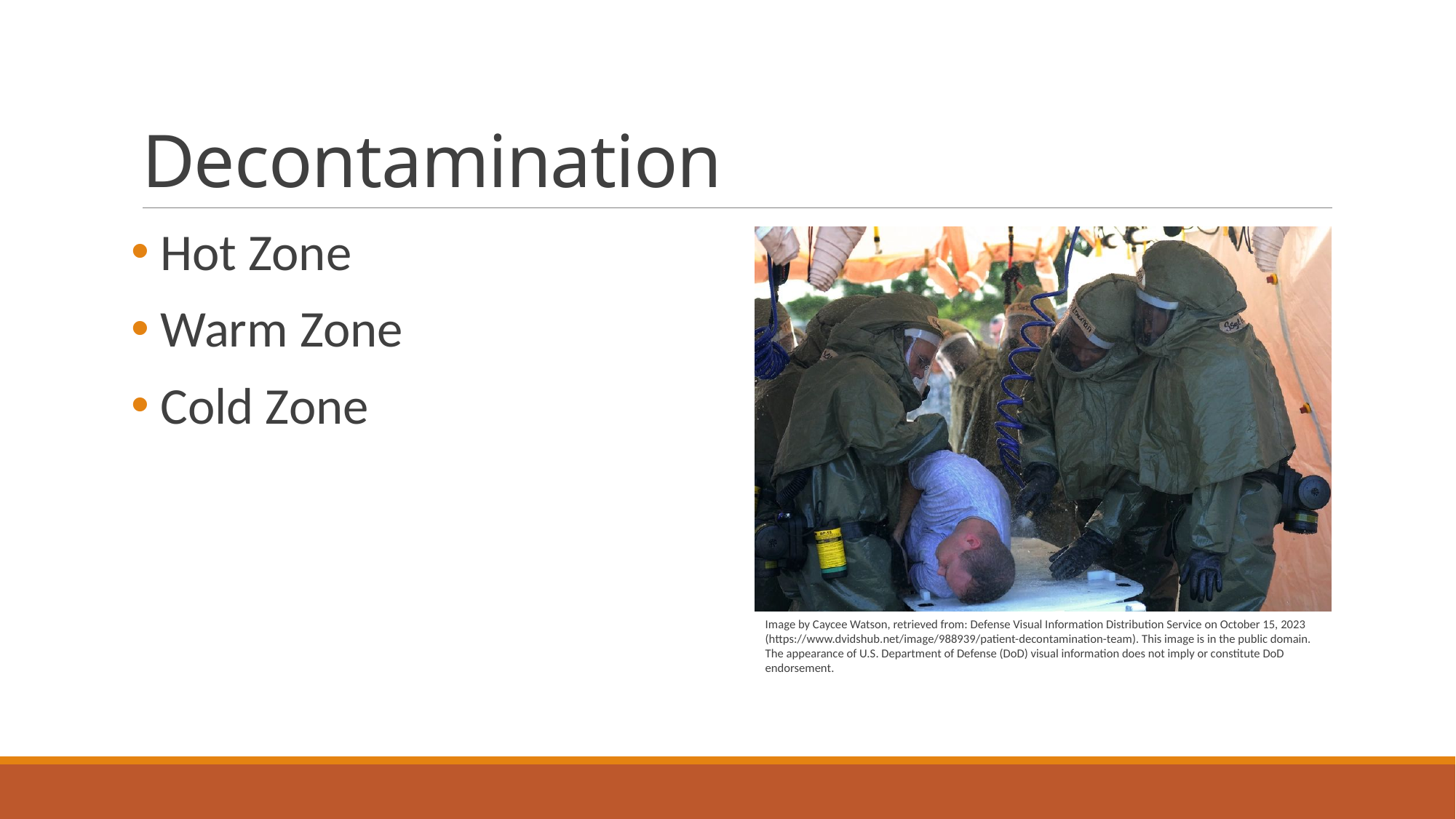

# Decontamination
 Hot Zone
 Warm Zone
 Cold Zone
Image by Caycee Watson, retrieved from: Defense Visual Information Distribution Service on October 15, 2023 (https://www.dvidshub.net/image/988939/patient-decontamination-team). This image is in the public domain. The appearance of U.S. Department of Defense (DoD) visual information does not imply or constitute DoD endorsement.

## Slide 15
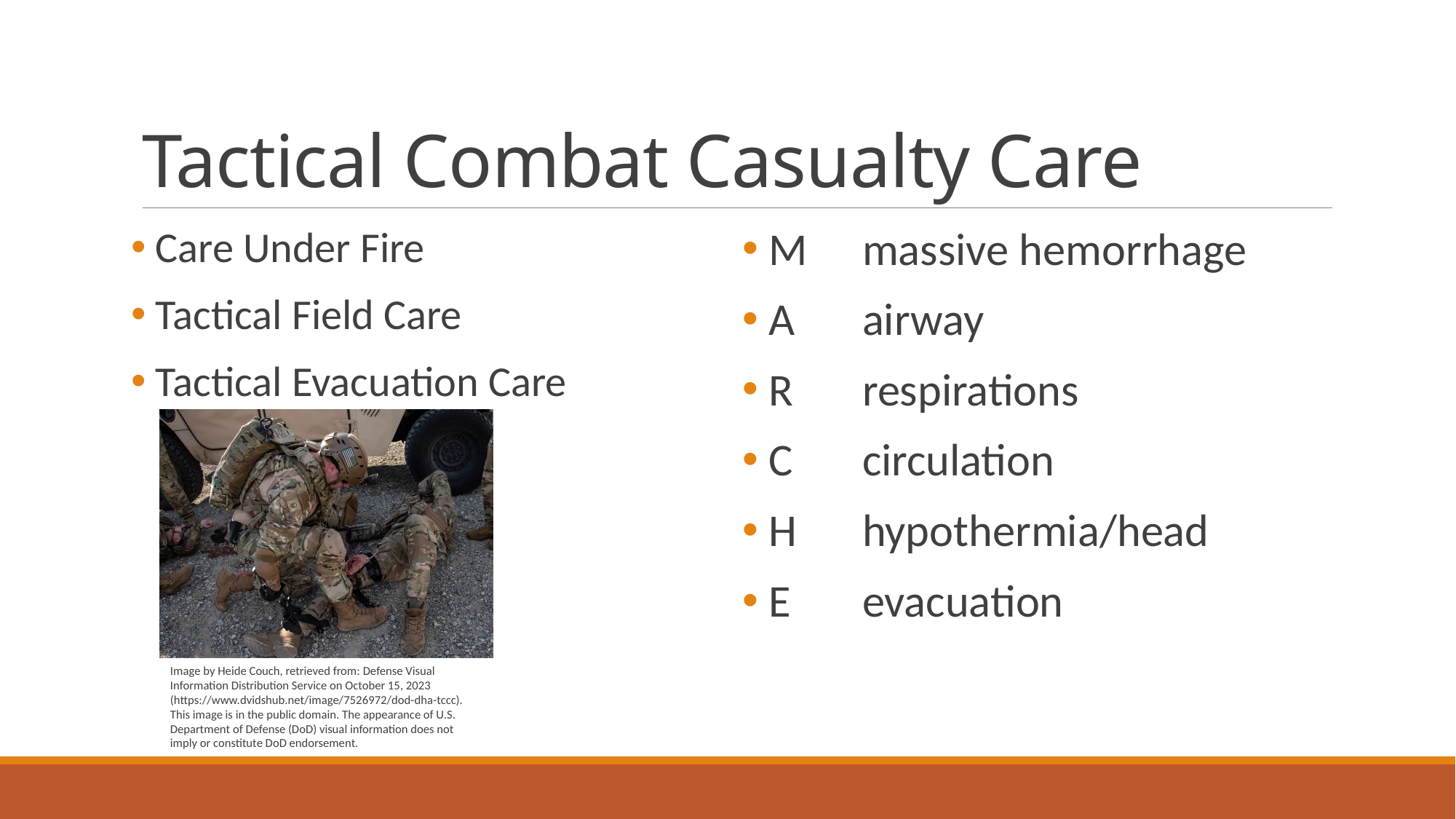

# Tactical Combat Casualty Care
 Care Under Fire
 Tactical Field Care
 Tactical Evacuation Care
 M	massive hemorrhage
 A	airway
 R	respirations
 C	circulation
 H	hypothermia/head
 E	evacuation
Image by Heide Couch, retrieved from: Defense Visual Information Distribution Service on October 15, 2023 (https://www.dvidshub.net/image/7526972/dod-dha-tccc). This image is in the public domain. The appearance of U.S. Department of Defense (DoD) visual information does not imply or constitute DoD endorsement.

## Slide 16
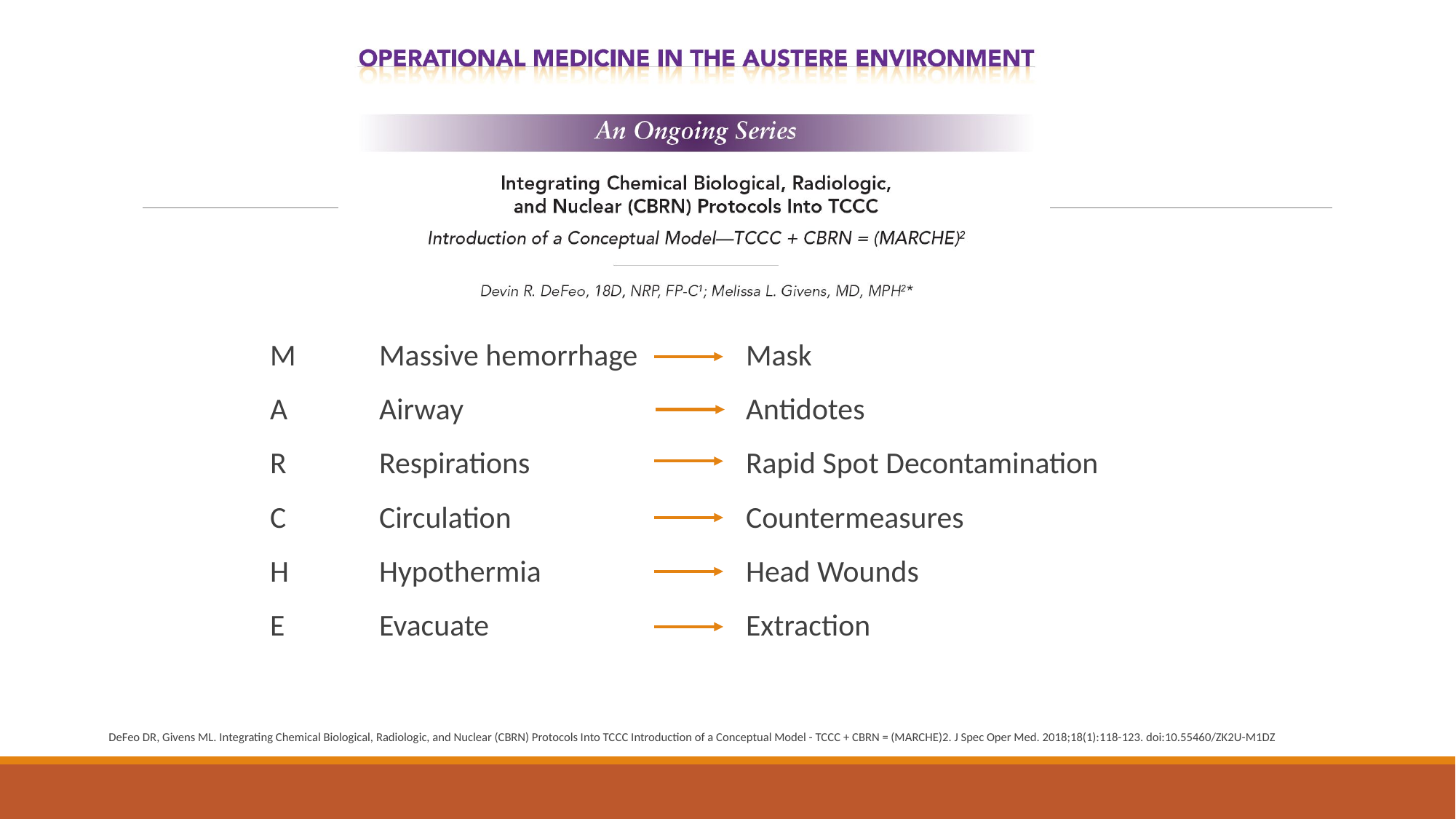

M	Massive hemorrhage
A	Airway
R	Respirations
C	Circulation
H	Hypothermia
E	Evacuate
Mask
Antidotes
Rapid Spot Decontamination
Countermeasures
Head Wounds
Extraction
DeFeo DR, Givens ML. Integrating Chemical Biological, Radiologic, and Nuclear (CBRN) Protocols Into TCCC Introduction of a Conceptual Model - TCCC + CBRN = (MARCHE)2. J Spec Oper Med. 2018;18(1):118-123. doi:10.55460/ZK2U-M1DZ

## Slide 17
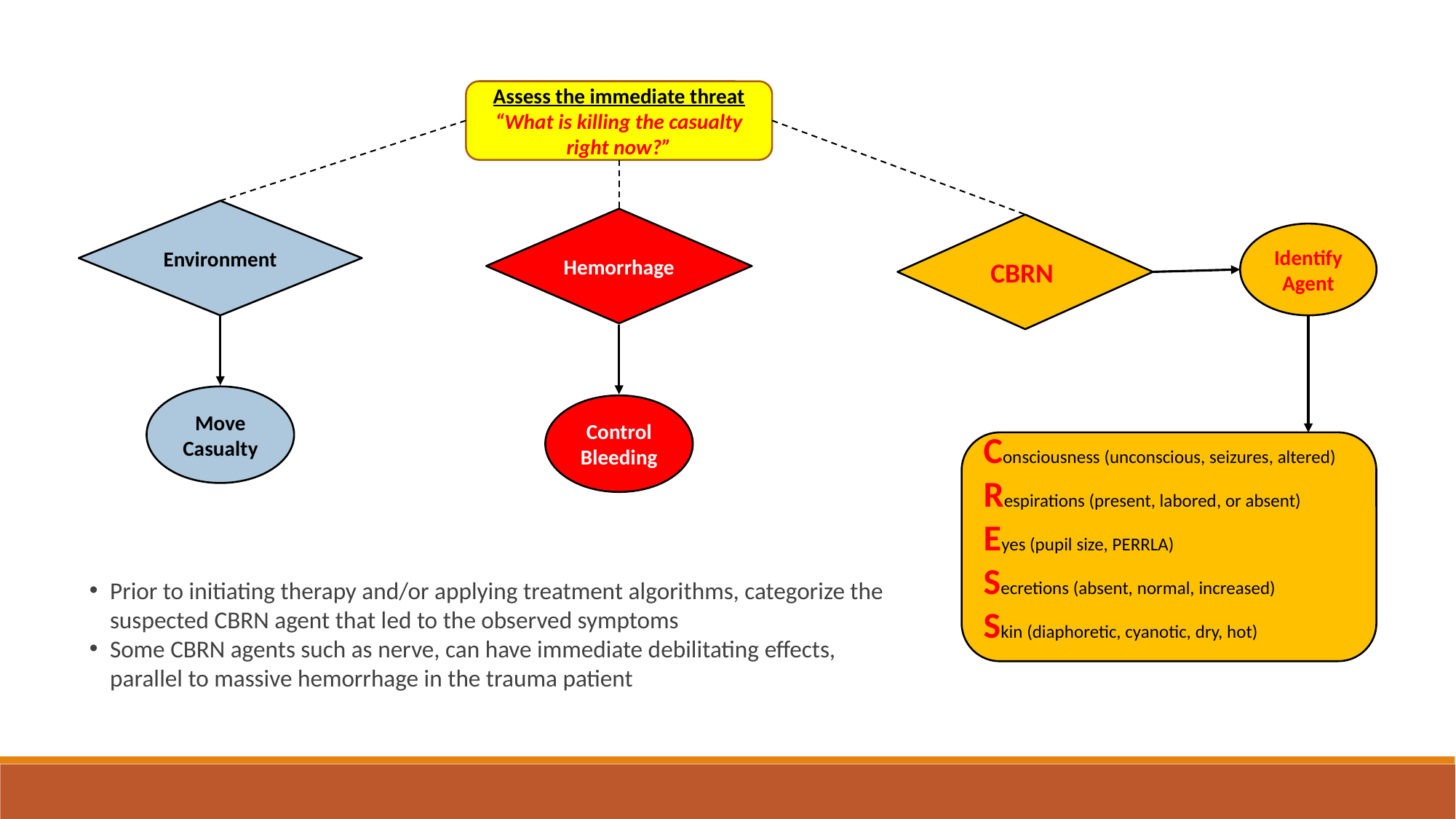

Assess the immediate threat
“What is killing the casualty right now?”
Environment
Hemorrhage
CBRN
Identify Agent
Move Casualty
Control Bleeding
Consciousness (unconscious, seizures, altered)
Respirations (present, labored, or absent)
Eyes (pupil size, PERRLA)
Secretions (absent, normal, increased)
Skin (diaphoretic, cyanotic, dry, hot)
Prior to initiating therapy and/or applying treatment algorithms, categorize the suspected CBRN agent that led to the observed symptoms
Some CBRN agents such as nerve, can have immediate debilitating effects, parallel to massive hemorrhage in the trauma patient

## Slide 18
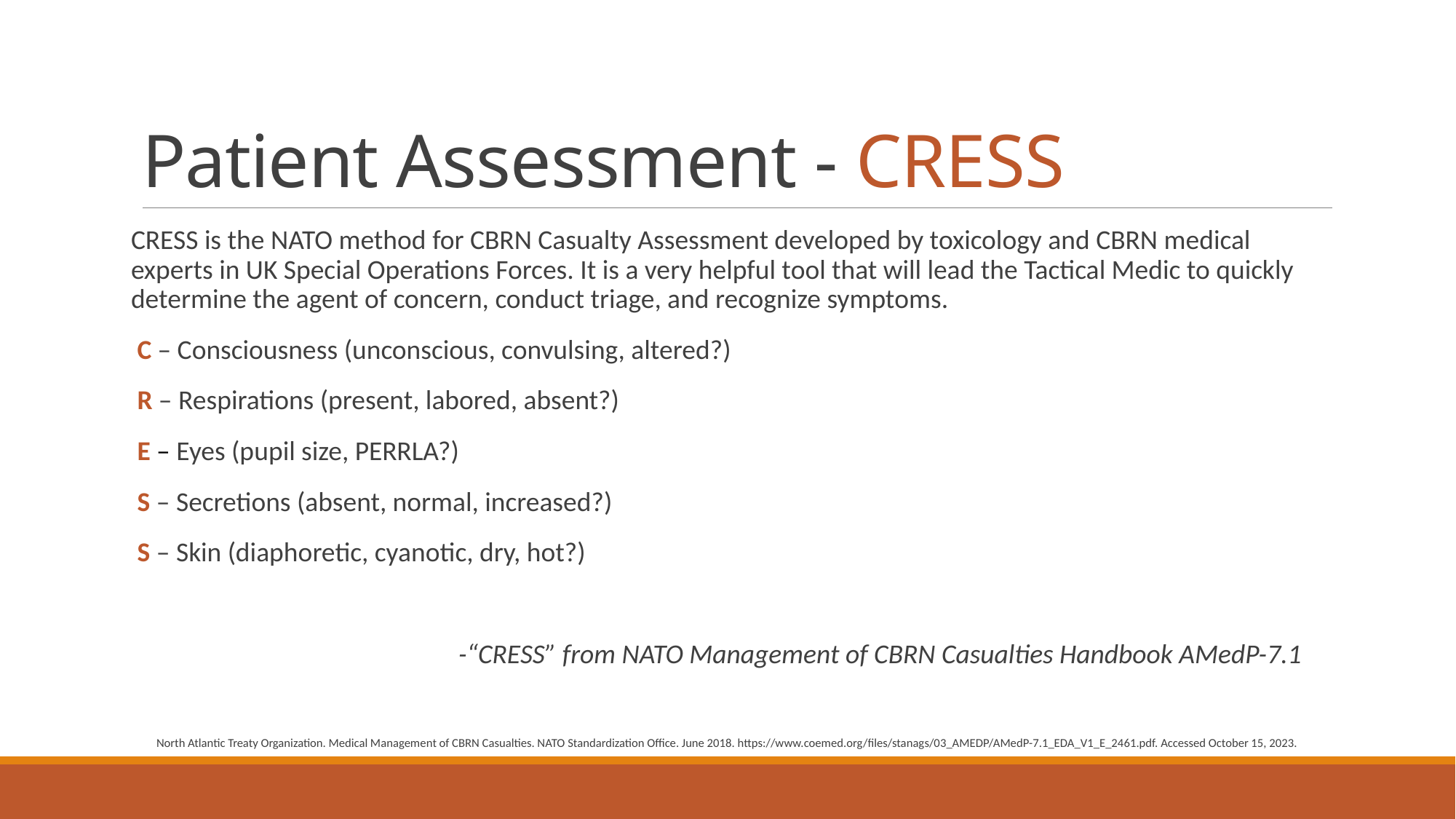

# Patient Assessment - CRESS
CRESS is the NATO method for CBRN Casualty Assessment developed by toxicology and CBRN medical experts in UK Special Operations Forces. It is a very helpful tool that will lead the Tactical Medic to quickly determine the agent of concern, conduct triage, and recognize symptoms.
C – Consciousness (unconscious, convulsing, altered?)
R – Respirations (present, labored, absent?)
E – Eyes (pupil size, PERRLA?)
S – Secretions (absent, normal, increased?)
S – Skin (diaphoretic, cyanotic, dry, hot?)
			-“CRESS” from NATO Management of CBRN Casualties Handbook AMedP-7.1
North Atlantic Treaty Organization. Medical Management of CBRN Casualties. NATO Standardization Office. June 2018. https://www.coemed.org/files/stanags/03_AMEDP/AMedP-7.1_EDA_V1_E_2461.pdf. Accessed October 15, 2023.

## Slide 19
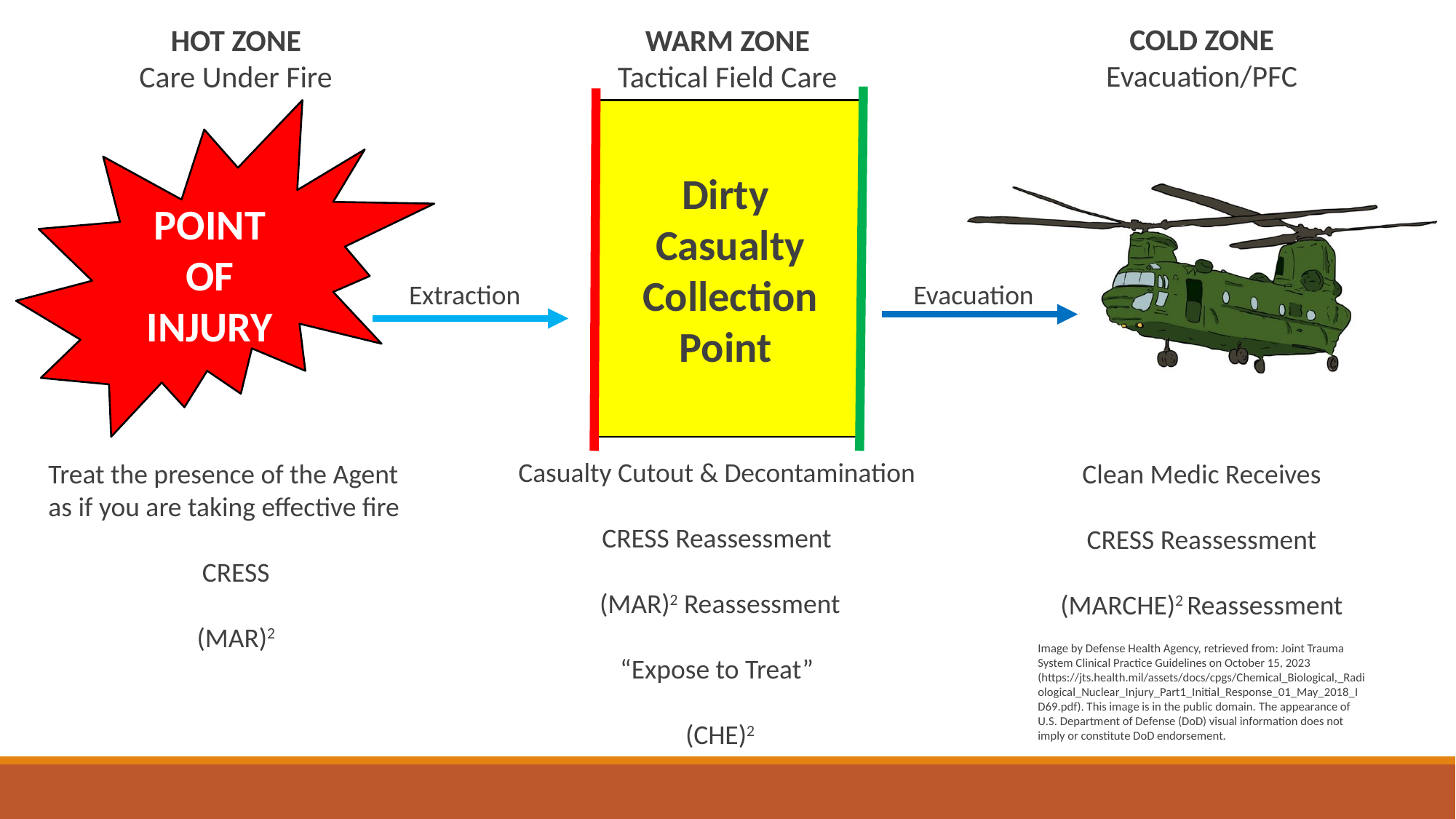

COLD ZONE
Evacuation/PFC
HOT ZONE
Care Under Fire
WARM ZONE
Tactical Field Care
POINT OF INJURY
Dirty
Casualty Collection Point
Evacuation
Extraction
Casualty Cutout & Decontamination
CRESS Reassessment
 (MAR)2 Reassessment
“Expose to Treat”
 (CHE)2
Treat the presence of the Agent as if you are taking effective fire
CRESS
(MAR)2
Clean Medic Receives
CRESS Reassessment
(MARCHE)2 Reassessment
Image by Defense Health Agency, retrieved from: Joint Trauma System Clinical Practice Guidelines on October 15, 2023 (https://jts.health.mil/assets/docs/cpgs/Chemical_Biological,_Radiological_Nuclear_Injury_Part1_Initial_Response_01_May_2018_ID69.pdf). This image is in the public domain. The appearance of U.S. Department of Defense (DoD) visual information does not imply or constitute DoD endorsement.

## Slide 20
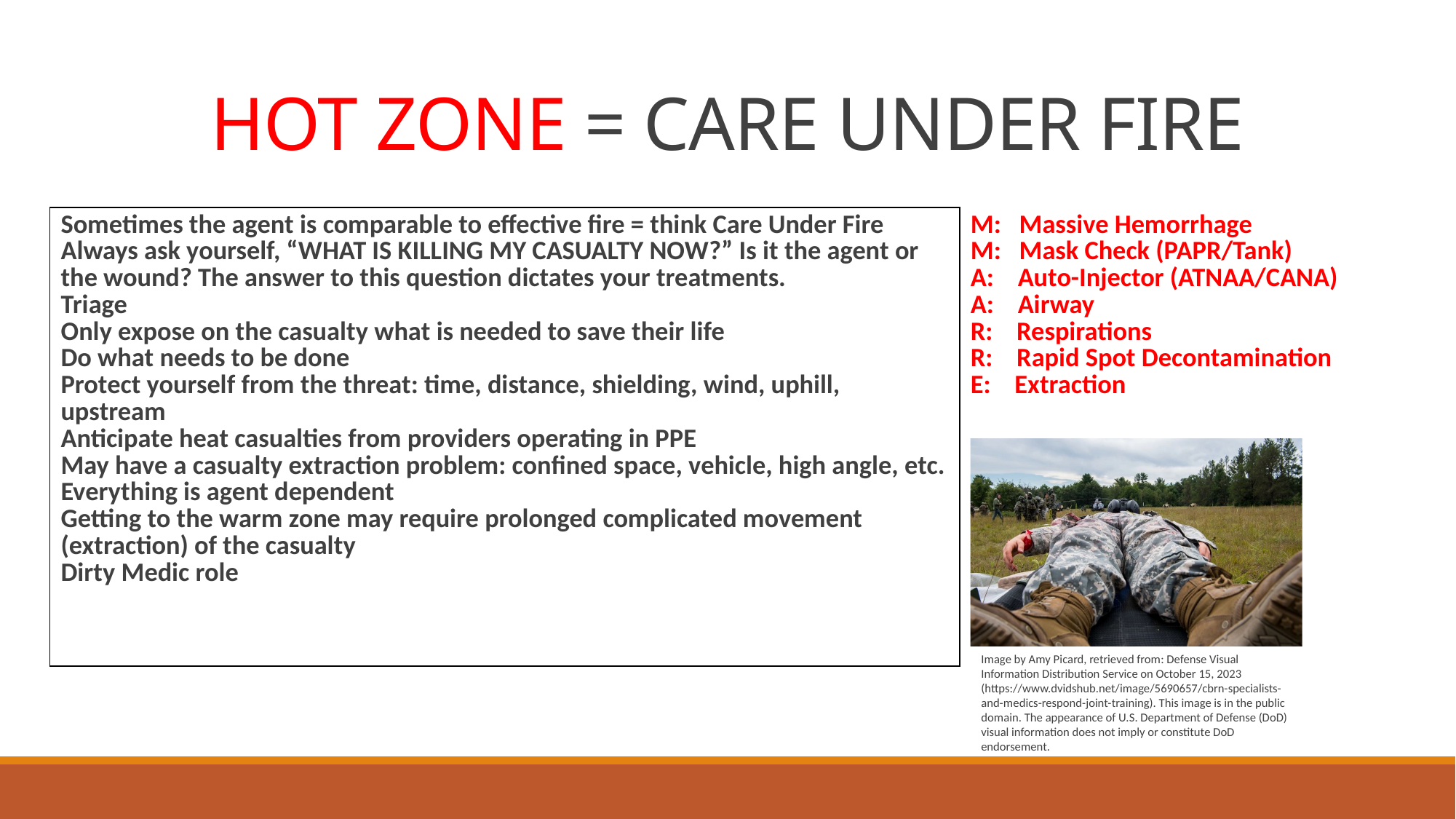

# HOT ZONE = CARE UNDER FIRE
| Sometimes the agent is comparable to effective fire = think Care Under Fire Always ask yourself, “WHAT IS KILLING MY CASUALTY NOW?” Is it the agent or the wound? The answer to this question dictates your treatments. Triage Only expose on the casualty what is needed to save their life Do what needs to be done Protect yourself from the threat: time, distance, shielding, wind, uphill, upstream Anticipate heat casualties from providers operating in PPE May have a casualty extraction problem: confined space, vehicle, high angle, etc. Everything is agent dependent Getting to the warm zone may require prolonged complicated movement (extraction) of the casualty Dirty Medic role | M: Massive Hemorrhage M: Mask Check (PAPR/Tank) A: Auto-Injector (ATNAA/CANA) A: Airway R: Respirations R: Rapid Spot Decontamination E: Extraction |
| --- | --- |
Image by Amy Picard, retrieved from: Defense Visual Information Distribution Service on October 15, 2023 (https://www.dvidshub.net/image/5690657/cbrn-specialists-and-medics-respond-joint-training). This image is in the public domain. The appearance of U.S. Department of Defense (DoD) visual information does not imply or constitute DoD endorsement.

## Slide 21
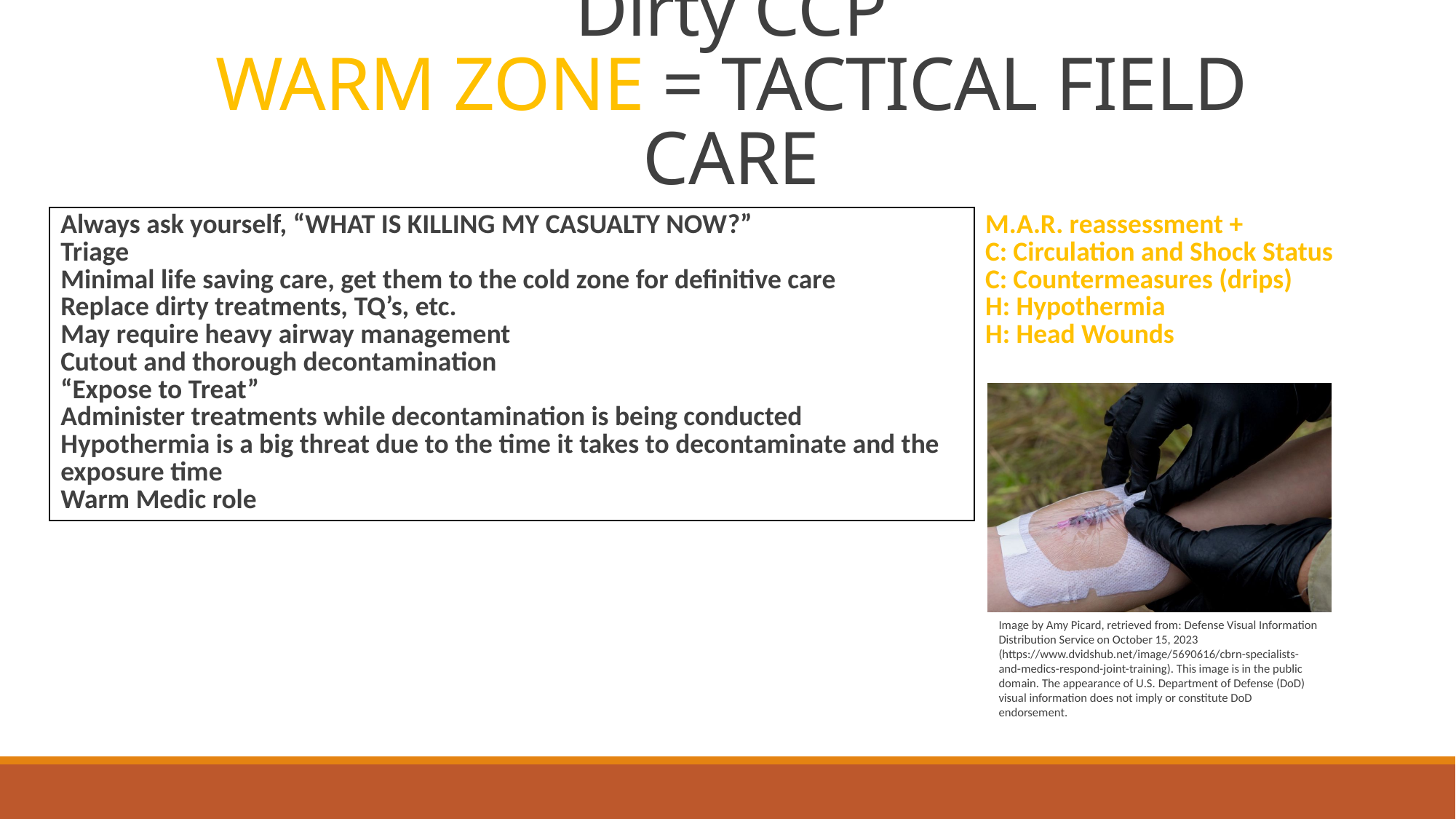

# Dirty CCP WARM ZONE = TACTICAL FIELD CARE
| Always ask yourself, “WHAT IS KILLING MY CASUALTY NOW?” Triage Minimal life saving care, get them to the cold zone for definitive care Replace dirty treatments, TQ’s, etc. May require heavy airway management Cutout and thorough decontamination “Expose to Treat” Administer treatments while decontamination is being conducted Hypothermia is a big threat due to the time it takes to decontaminate and the exposure time Warm Medic role | M.A.R. reassessment + C: Circulation and Shock Status C: Countermeasures (drips) H: Hypothermia H: Head Wounds |
| --- | --- |
Image by Amy Picard, retrieved from: Defense Visual Information Distribution Service on October 15, 2023 (https://www.dvidshub.net/image/5690616/cbrn-specialists-and-medics-respond-joint-training). This image is in the public domain. The appearance of U.S. Department of Defense (DoD) visual information does not imply or constitute DoD endorsement.

## Slide 22
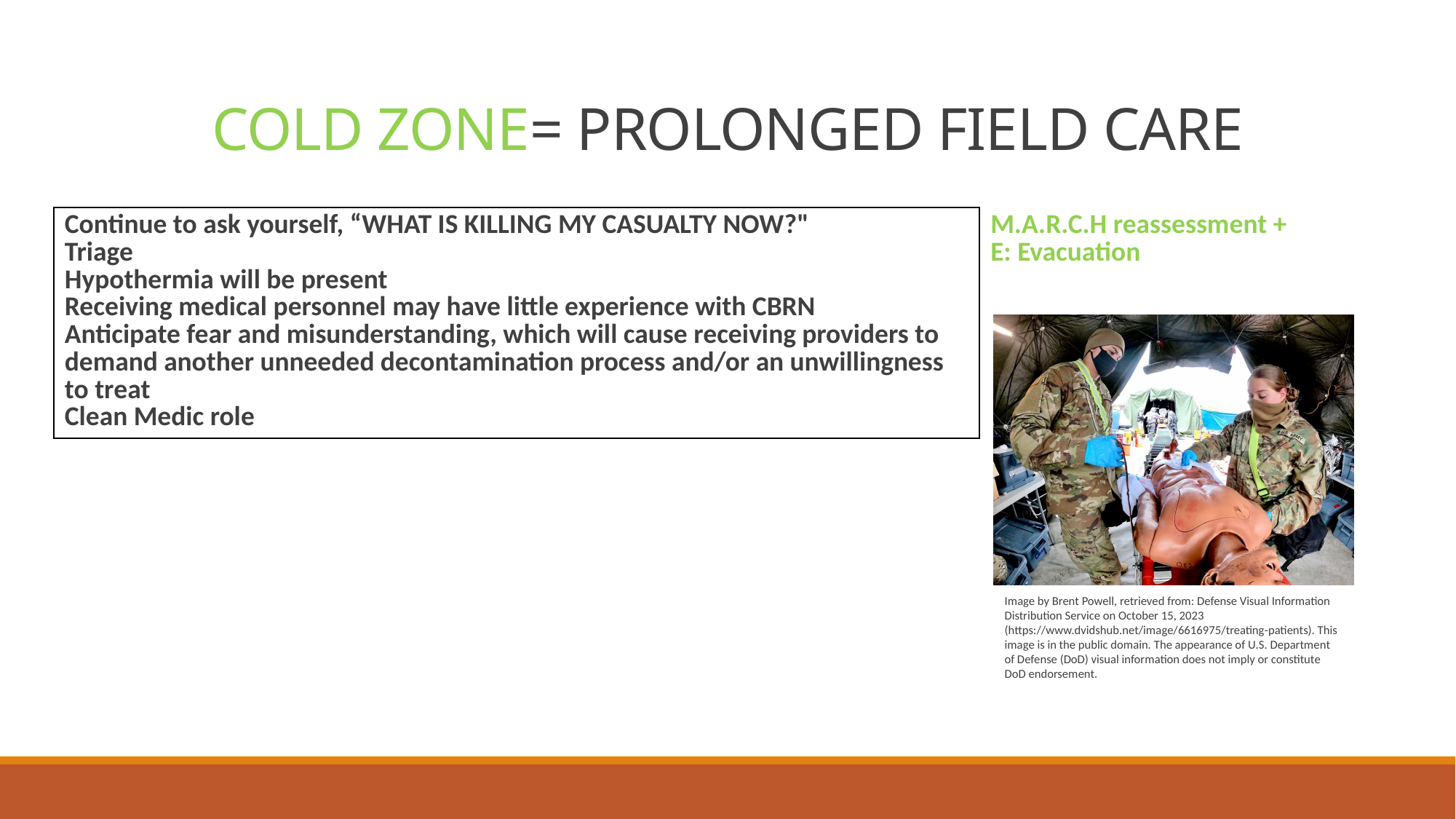

# COLD ZONE= PROLONGED FIELD CARE
| Continue to ask yourself, “WHAT IS KILLING MY CASUALTY NOW?" Triage Hypothermia will be present Receiving medical personnel may have little experience with CBRN Anticipate fear and misunderstanding, which will cause receiving providers to demand another unneeded decontamination process and/or an unwillingness to treat Clean Medic role | M.A.R.C.H reassessment + E: Evacuation |
| --- | --- |
Image by Brent Powell, retrieved from: Defense Visual Information Distribution Service on October 15, 2023 (https://www.dvidshub.net/image/6616975/treating-patients). This image is in the public domain. The appearance of U.S. Department of Defense (DoD) visual information does not imply or constitute DoD endorsement.

## Slide 23
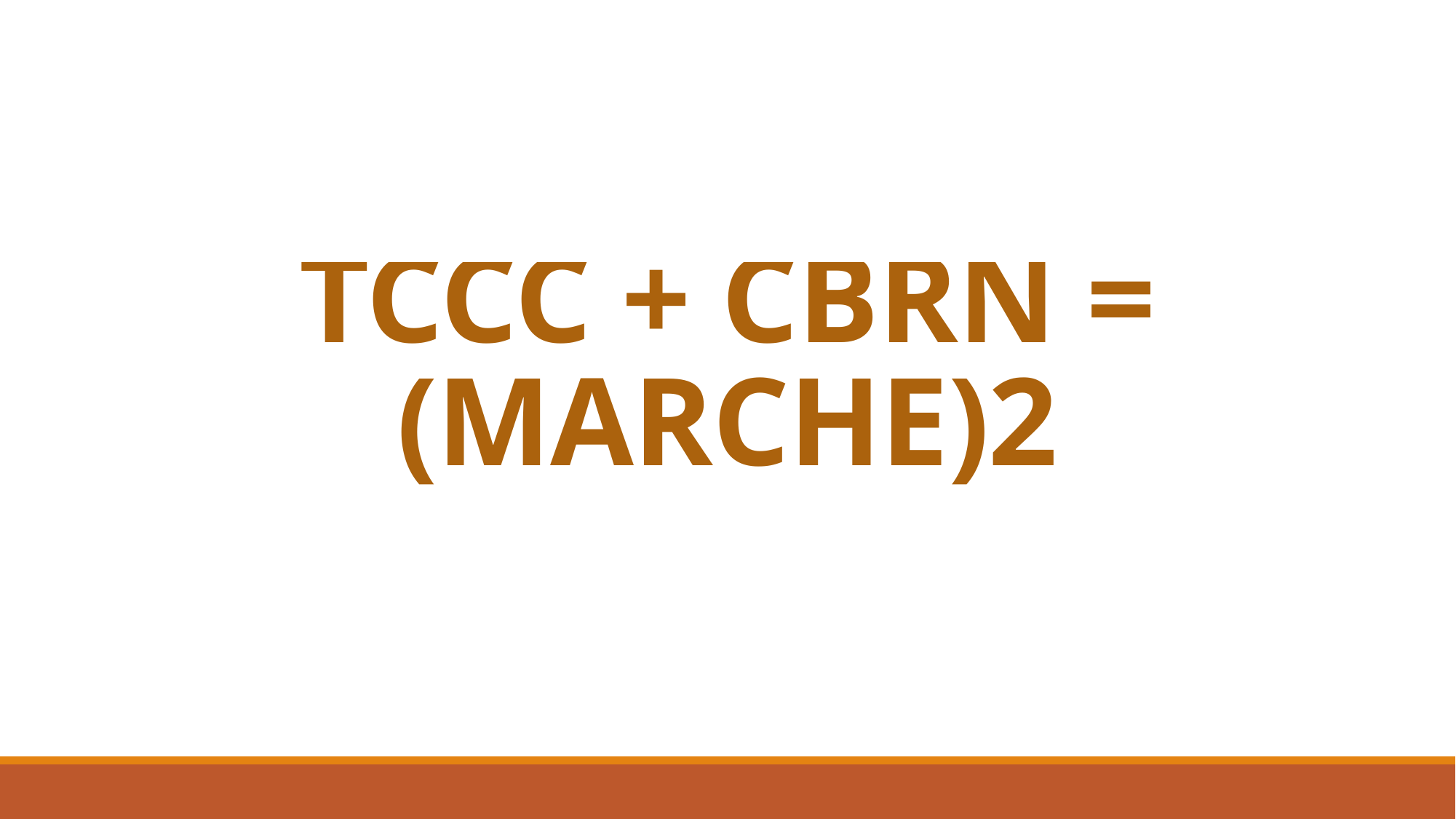

# TCCC + CBRN = (MARCHE)2

## Slide 24
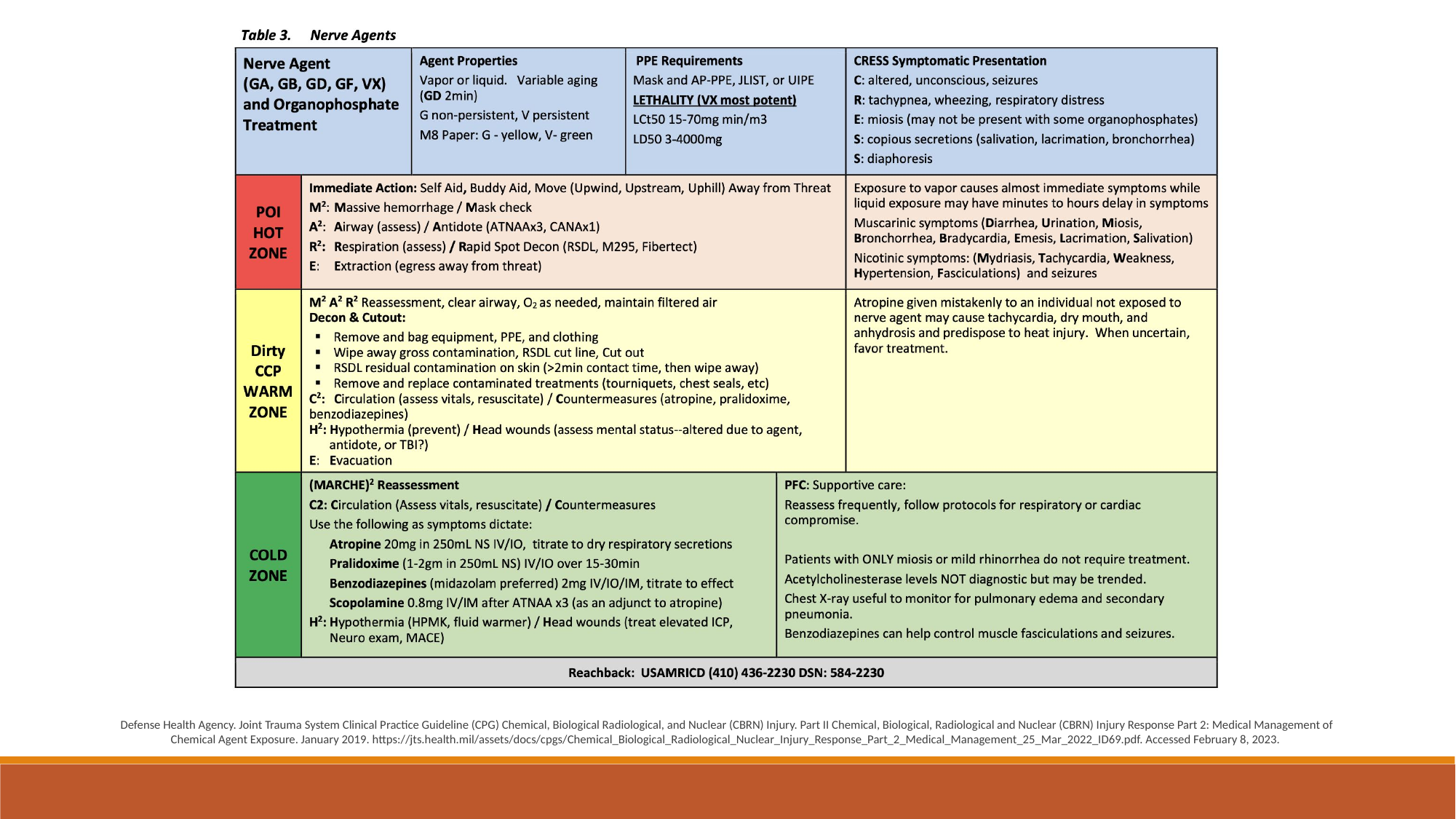

Defense Health Agency. Joint Trauma System Clinical Practice Guideline (CPG) Chemical, Biological Radiological, and Nuclear (CBRN) Injury. Part II Chemical, Biological, Radiological and Nuclear (CBRN) Injury Response Part 2: Medical Management of Chemical Agent Exposure. January 2019. https://jts.health.mil/assets/docs/cpgs/Chemical_Biological_Radiological_Nuclear_Injury_Response_Part_2_Medical_Management_25_Mar_2022_ID69.pdf. Accessed February 8, 2023.

## Slide 25
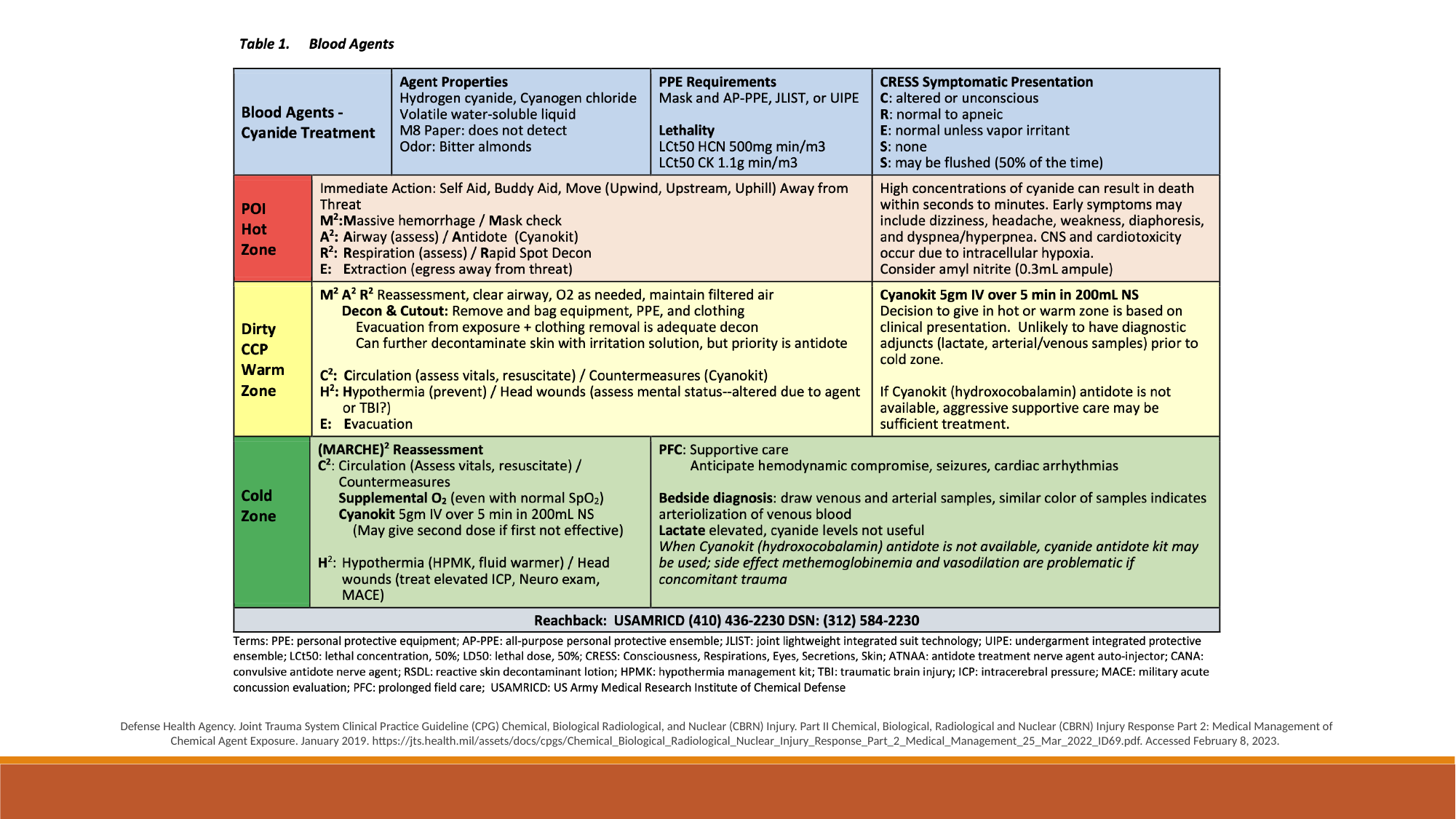

Defense Health Agency. Joint Trauma System Clinical Practice Guideline (CPG) Chemical, Biological Radiological, and Nuclear (CBRN) Injury. Part II Chemical, Biological, Radiological and Nuclear (CBRN) Injury Response Part 2: Medical Management of Chemical Agent Exposure. January 2019. https://jts.health.mil/assets/docs/cpgs/Chemical_Biological_Radiological_Nuclear_Injury_Response_Part_2_Medical_Management_25_Mar_2022_ID69.pdf. Accessed February 8, 2023.

## Slide 26
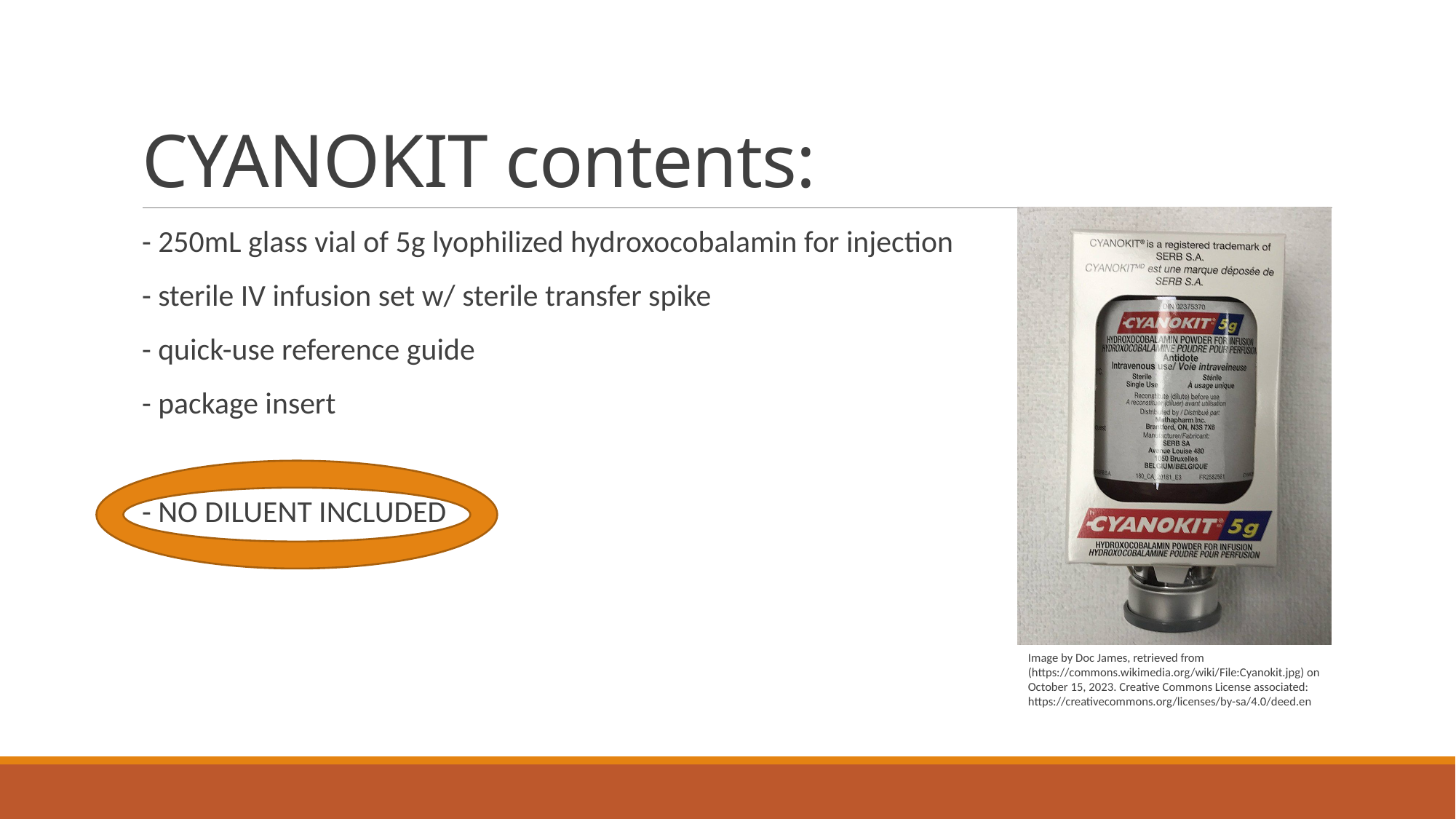

# CYANOKIT contents:
Image by Doc James, retrieved from (https://commons.wikimedia.org/wiki/File:Cyanokit.jpg) on October 15, 2023. Creative Commons License associated: https://creativecommons.org/licenses/by-sa/4.0/deed.en
- 250mL glass vial of 5g lyophilized hydroxocobalamin for injection
- sterile IV infusion set w/ sterile transfer spike
- quick-use reference guide
- package insert
- NO DILUENT INCLUDED

## Slide 27
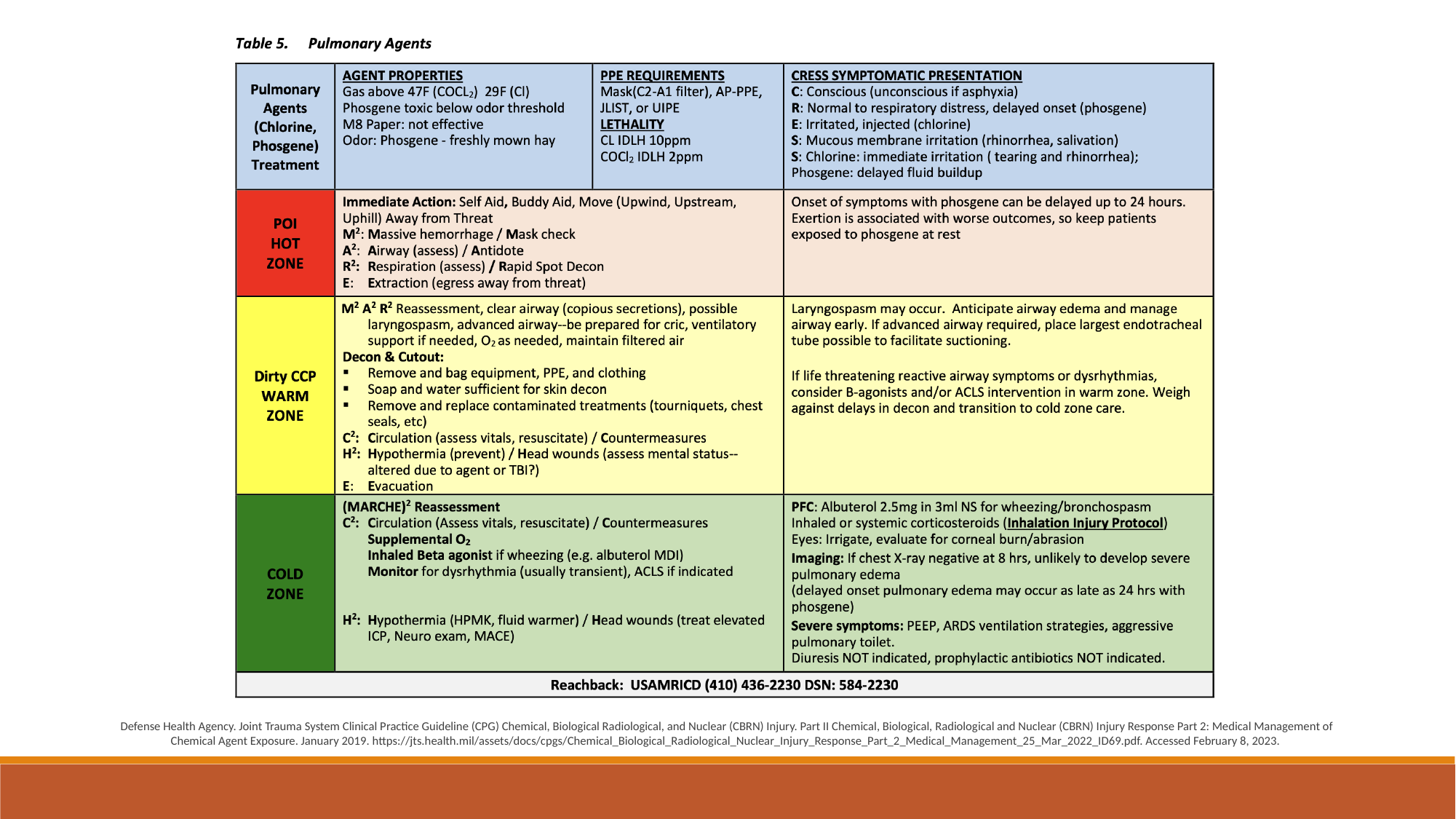

Defense Health Agency. Joint Trauma System Clinical Practice Guideline (CPG) Chemical, Biological Radiological, and Nuclear (CBRN) Injury. Part II Chemical, Biological, Radiological and Nuclear (CBRN) Injury Response Part 2: Medical Management of Chemical Agent Exposure. January 2019. https://jts.health.mil/assets/docs/cpgs/Chemical_Biological_Radiological_Nuclear_Injury_Response_Part_2_Medical_Management_25_Mar_2022_ID69.pdf. Accessed February 8, 2023.

## Slide 28
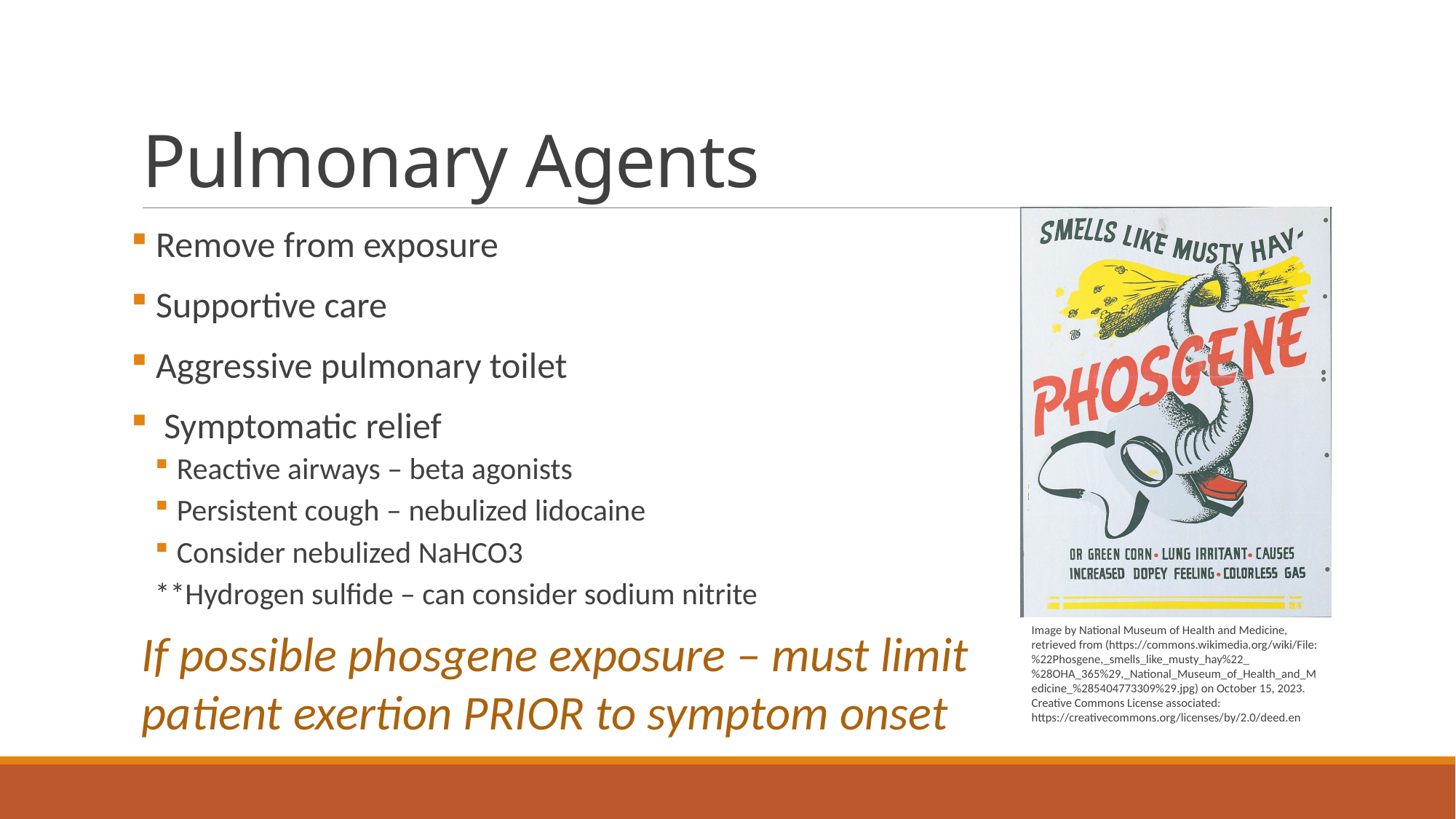

# Pulmonary Agents
Image by National Museum of Health and Medicine, retrieved from (https://commons.wikimedia.org/wiki/File:%22Phosgene,_smells_like_musty_hay%22_%28OHA_365%29,_National_Museum_of_Health_and_Medicine_%285404773309%29.jpg) on October 15, 2023. Creative Commons License associated: https://creativecommons.org/licenses/by/2.0/deed.en
 Remove from exposure
 Supportive care
 Aggressive pulmonary toilet
 Symptomatic relief
Reactive airways – beta agonists
Persistent cough – nebulized lidocaine
Consider nebulized NaHCO3
**Hydrogen sulfide – can consider sodium nitrite
If possible phosgene exposure – must limit patient exertion PRIOR to symptom onset

## Slide 29
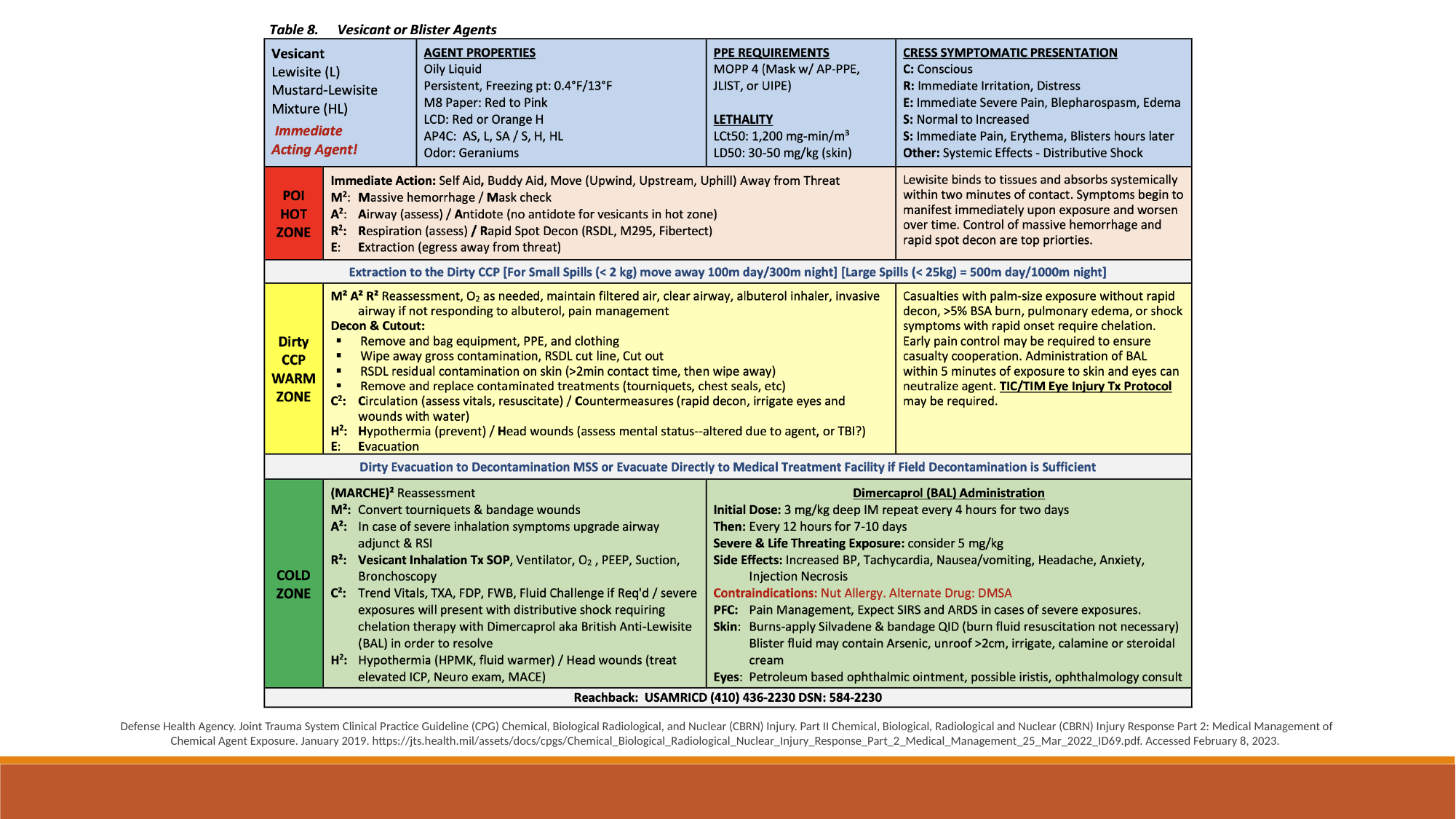

Defense Health Agency. Joint Trauma System Clinical Practice Guideline (CPG) Chemical, Biological Radiological, and Nuclear (CBRN) Injury. Part II Chemical, Biological, Radiological and Nuclear (CBRN) Injury Response Part 2: Medical Management of Chemical Agent Exposure. January 2019. https://jts.health.mil/assets/docs/cpgs/Chemical_Biological_Radiological_Nuclear_Injury_Response_Part_2_Medical_Management_25_Mar_2022_ID69.pdf. Accessed February 8, 2023.

## Slide 30
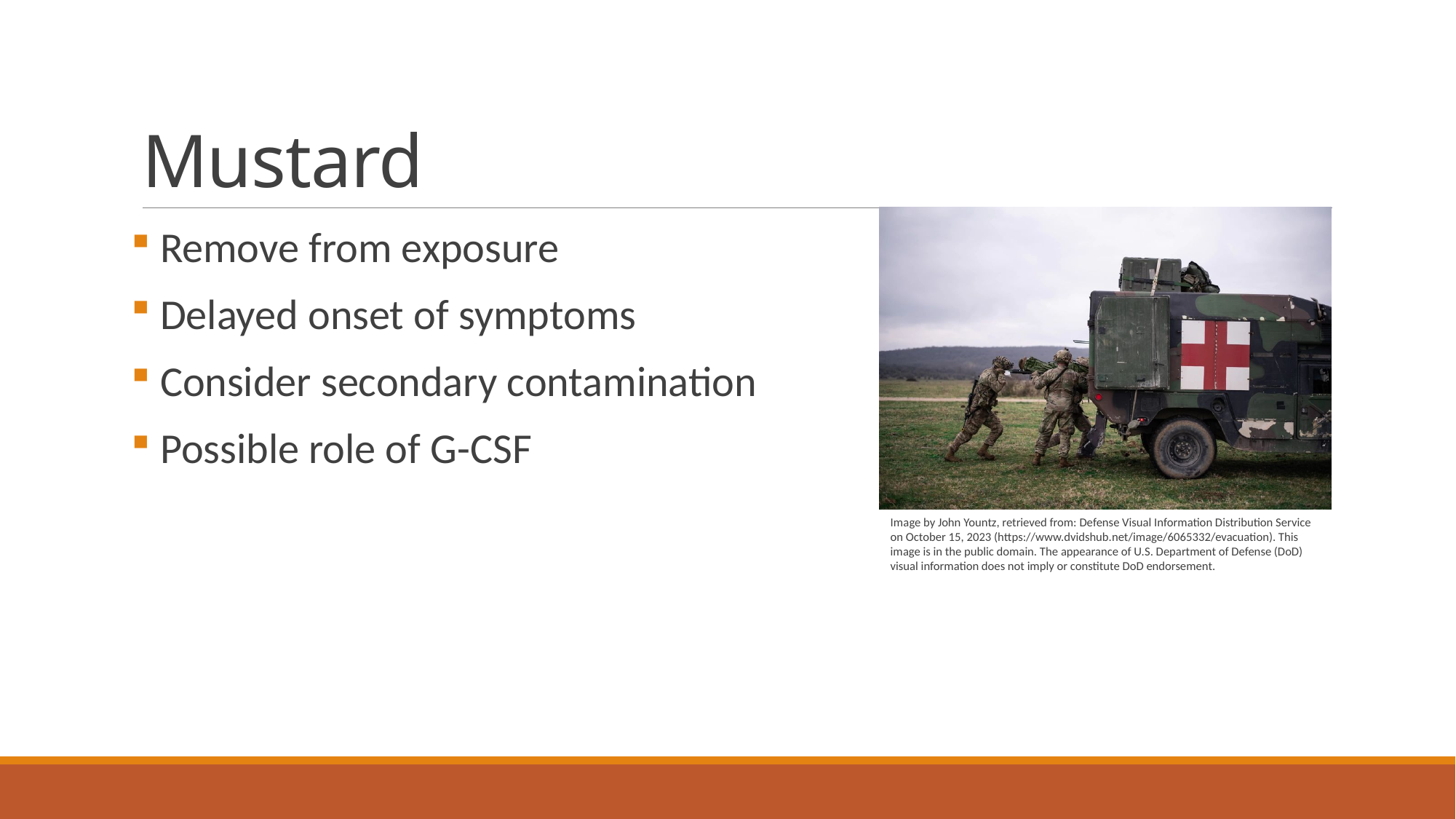

# Mustard
 Remove from exposure
 Delayed onset of symptoms
 Consider secondary contamination
 Possible role of G-CSF
Image by John Yountz, retrieved from: Defense Visual Information Distribution Service on October 15, 2023 (https://www.dvidshub.net/image/6065332/evacuation). This image is in the public domain. The appearance of U.S. Department of Defense (DoD) visual information does not imply or constitute DoD endorsement.

## Slide 31
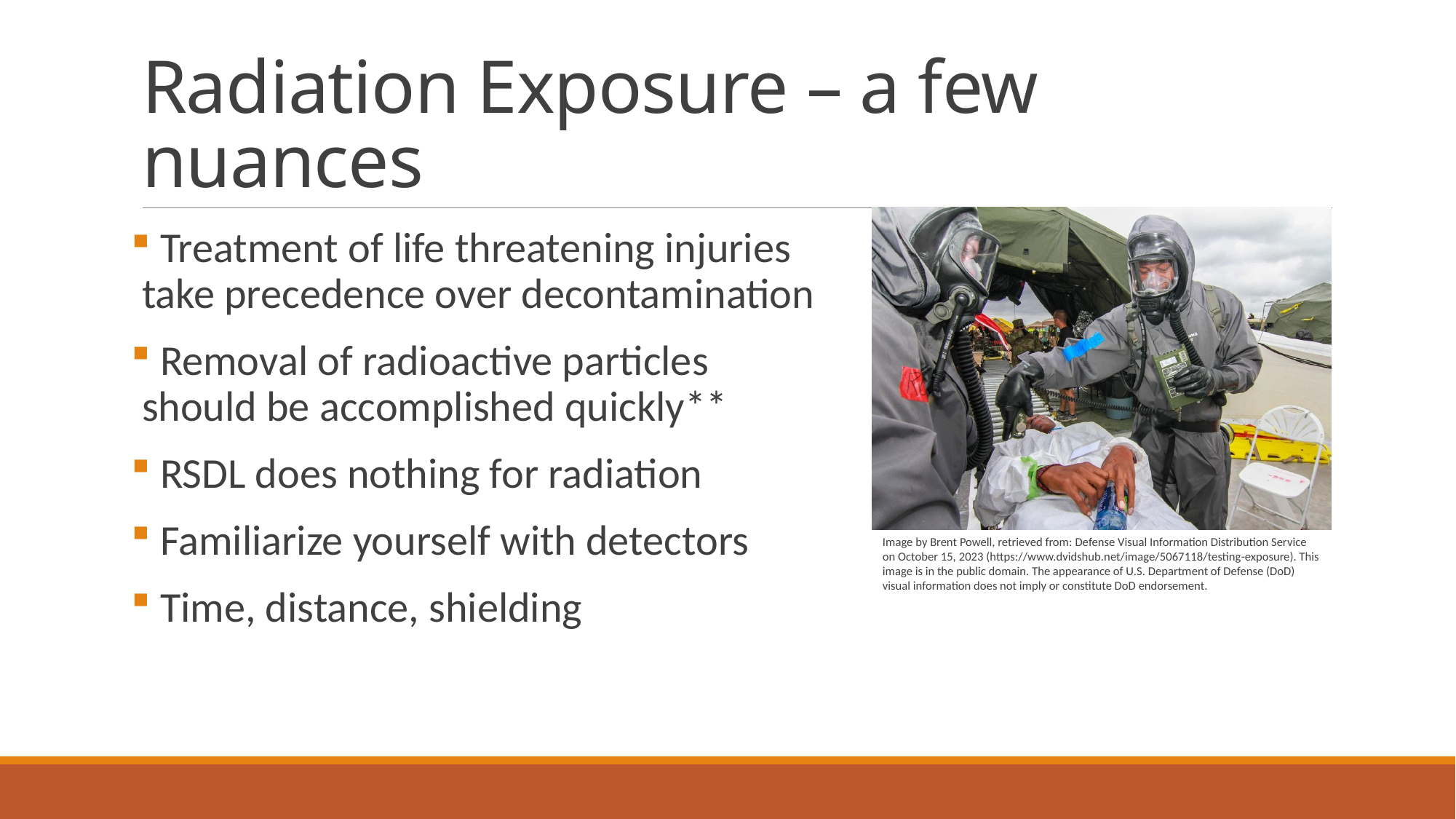

# Radiation Exposure – a few nuances
 Treatment of life threatening injuries take precedence over decontamination
 Removal of radioactive particles should be accomplished quickly**
 RSDL does nothing for radiation
 Familiarize yourself with detectors
 Time, distance, shielding
Image by Brent Powell, retrieved from: Defense Visual Information Distribution Service on October 15, 2023 (https://www.dvidshub.net/image/5067118/testing-exposure). This image is in the public domain. The appearance of U.S. Department of Defense (DoD) visual information does not imply or constitute DoD endorsement.

## Slide 32
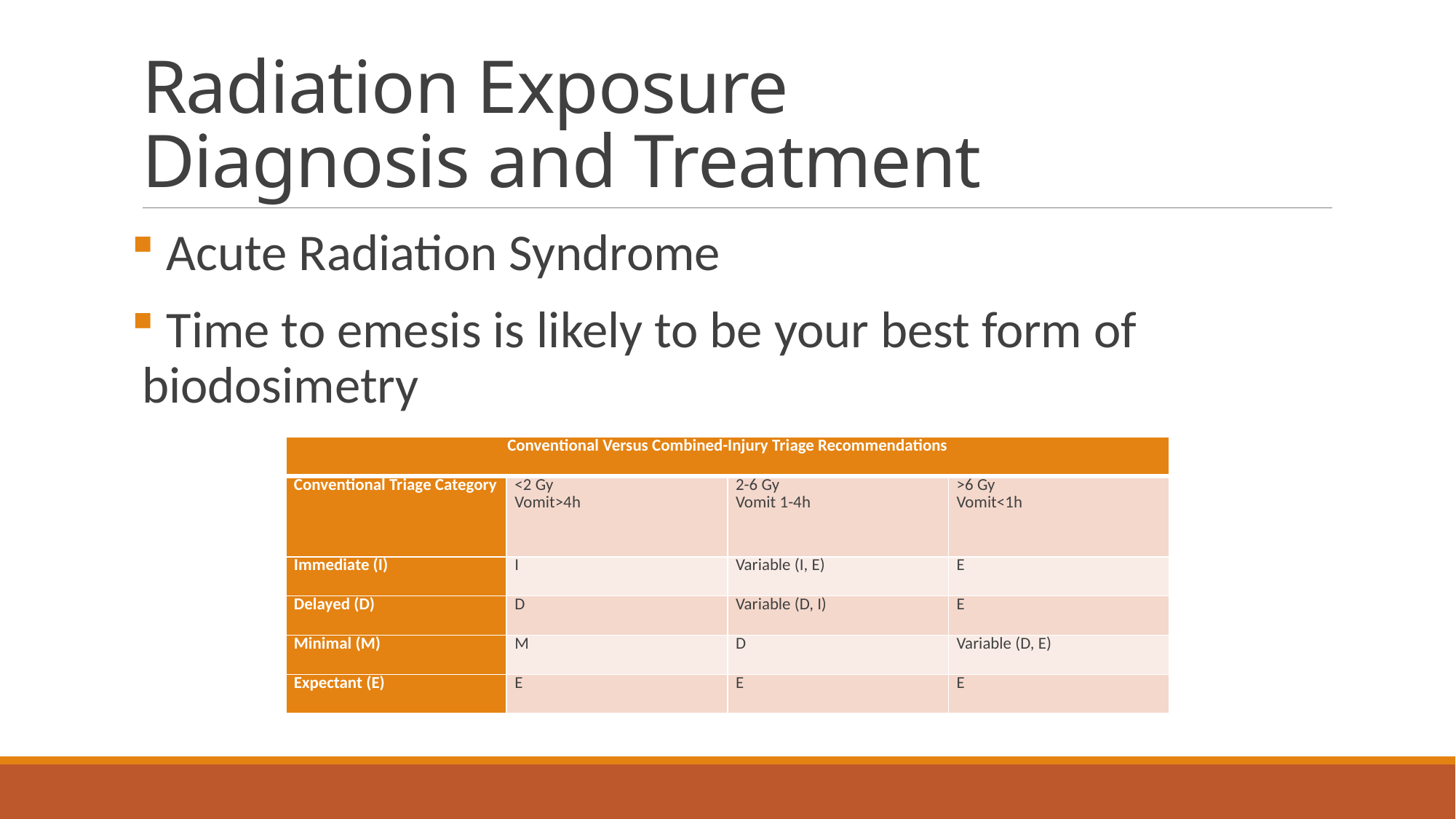

# Radiation Exposure Diagnosis and Treatment
 Acute Radiation Syndrome
 Time to emesis is likely to be your best form of biodosimetry
| Conventional Versus Combined-Injury Triage Recommendations | | | |
| --- | --- | --- | --- |
| Conventional Triage Category | <2 Gy Vomit>4h | 2-6 Gy Vomit 1-4h | >6 Gy Vomit<1h |
| Immediate (I) | I | Variable (I, E) | E |
| Delayed (D) | D | Variable (D, I) | E |
| Minimal (M) | M | D | Variable (D, E) |
| Expectant (E) | E | E | E |

## Slide 33
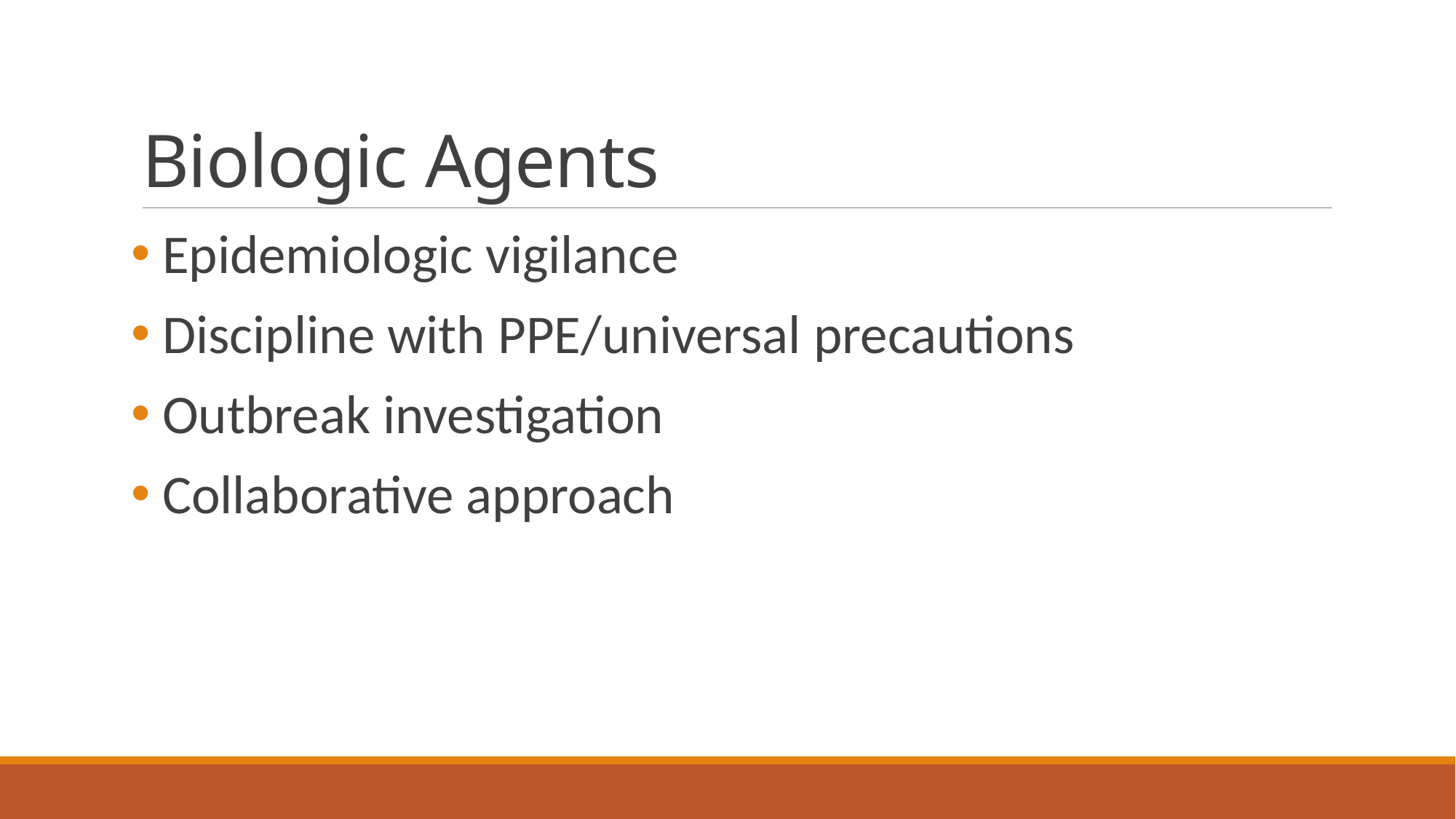

# Biologic Agents
 Epidemiologic vigilance
 Discipline with PPE/universal precautions
 Outbreak investigation
 Collaborative approach

## Slide 34
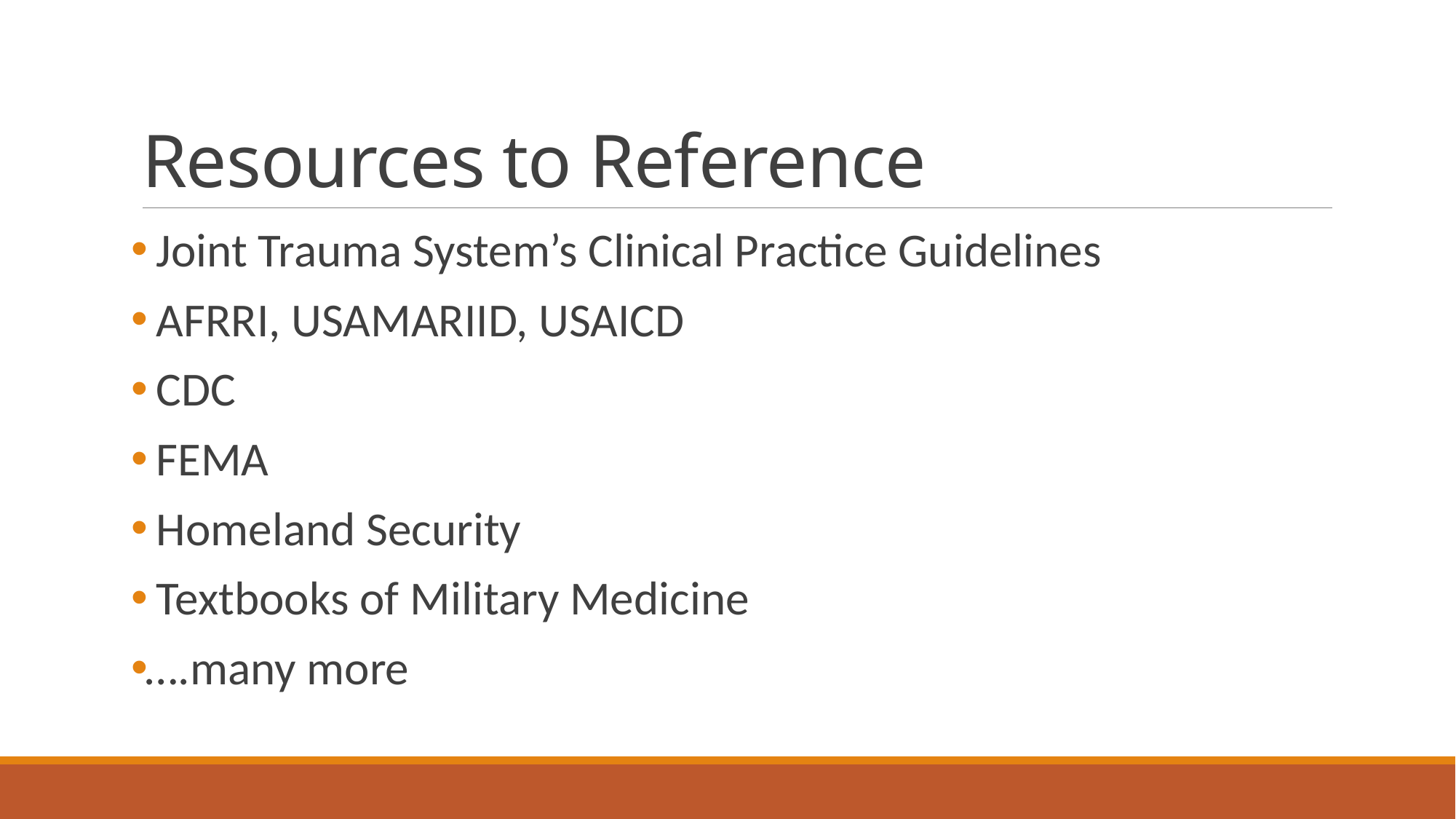

# Resources to Reference
 Joint Trauma System’s Clinical Practice Guidelines
 AFRRI, USAMARIID, USAICD
 CDC
 FEMA
 Homeland Security
 Textbooks of Military Medicine
….many more

## Slide 35
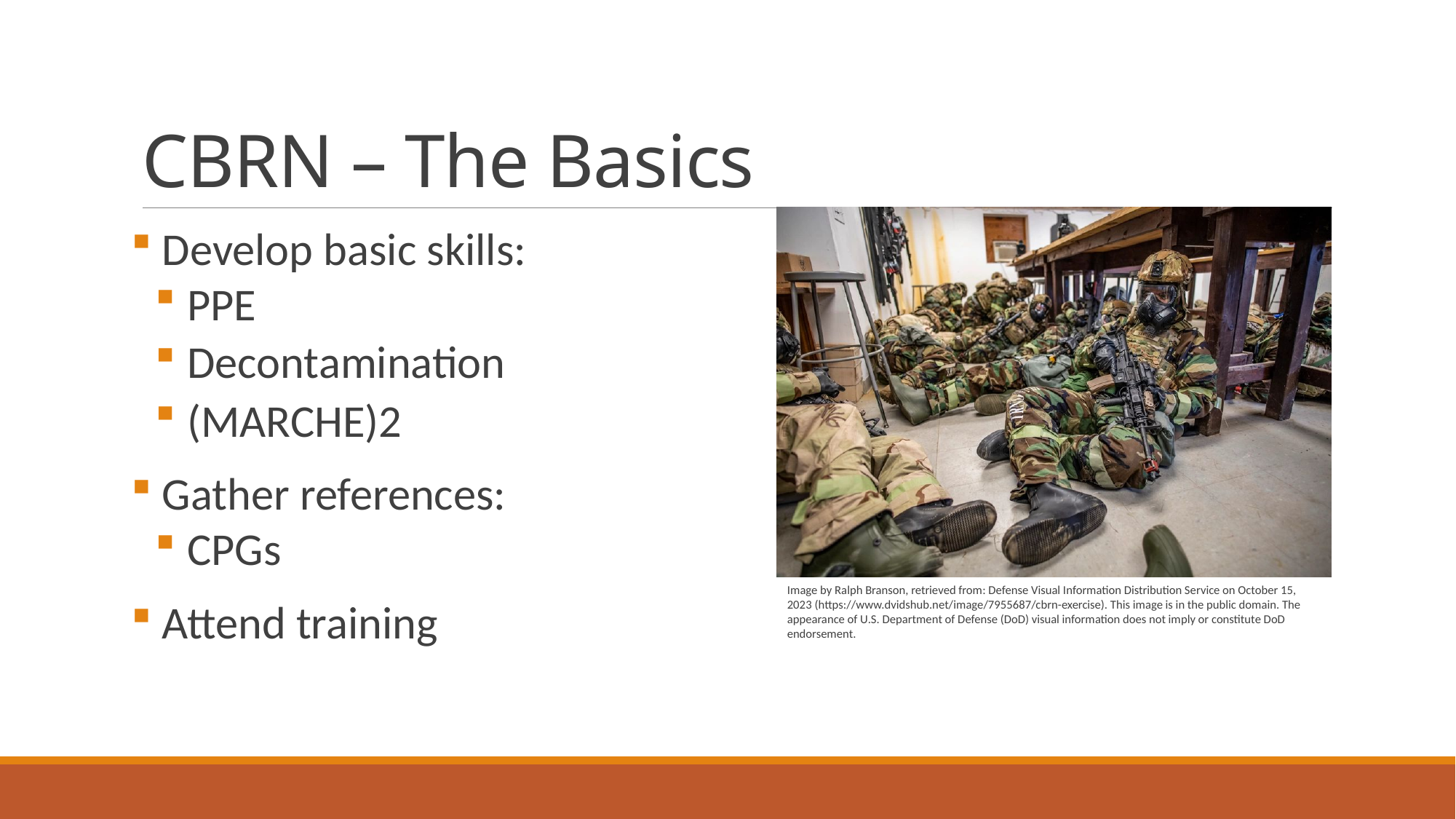

# CBRN – The Basics
 Develop basic skills:
 PPE
 Decontamination
 (MARCHE)2
 Gather references:
 CPGs
 Attend training
Image by Ralph Branson, retrieved from: Defense Visual Information Distribution Service on October 15, 2023 (https://www.dvidshub.net/image/7955687/cbrn-exercise). This image is in the public domain. The appearance of U.S. Department of Defense (DoD) visual information does not imply or constitute DoD endorsement.
