# Supplementary material for: Introduction to Treating Patients Exposed to Chemical, Biological, Radiological, and Nuclear (CBRN) Threats: A Military Medical Case-Based Curriculum
Source: MedEdPORTAL. 2024 Sep 13;20:11433. doi: 10.15766/mep_2374-8265.11433 (PMC11393073; doi:10.15766/mep_2374-8265.11433)
Supplement: Supplementary file 1 — Session One Lecture.pptxSupplemental Resources for Session One.docxCBRN Patient Worksheet.docxPatient Worksheet Video - Introduction to CBRN Patient.mp4Patient Worksheet Video - CBRN Corpsman Response.mp4Patient Worksheet Video - Physician Assessment.mp4Check on Knowledge Form.docxCBRN Patient Worksheet - Facilitator Version.docxFacilitator Guide.docxStudent Survey.docxSupplemental Resources for Session Two.docx [file mep_2374-8265.11433-s001.zip › C. CBRN Patient Worksheet.docx]

**Appendix C. CBRN Patient Worksheet**

The following worksheet was originally formatted for distribution to students with embedded hyperlinks (indicated in blue and underlined) for ease of access during the activity. It is meant to be completed as a student group, with each group filling out one worksheet. Time available for completion is 60-90 minutes.

Given its current distribution as a reusable educational material, the hyperlinks have been removed from the document and replaced with references to optional supporting materials.

For your reference, the Check on Knowledge Form is available in Appendix G. The Student Survey is available in Appendix J. The feedback for each section of the CBRN Patient Worksheet is available as part of the Facilitator’s Version of the worksheet in Appendix H. The videos are available in Appendices D, E, and F, as noted within the worksheet.

| INTRODUCTION TO THE CBRN PATIENT CASE SCENARIO |
| --- |
| **OBJECTIVES** |
| - Employ the CRESS algorithm to rapidly evaluate for potential chemical agent exposure - Develop an organized approach to the evaluation and treatment of a CBRN patient using principles of (MARCHE)2 - Compare and contrast the presenting signs and symptoms of nerve and pulmonary agents - Describe the mechanism of action for nerve agents and how this informs treatment of these patients |
| **INSTRUCTIONS** |
| **Pre-work:** Make sure you have read the JTS Clinical Practice Guidelines prior to beginning the worksheet.  **Getting Started**: One team member needs to make a copy of this worksheet on the Google drive platform, rename it (Company-Platoon-Fire Team, i.e. A-1-1a), and share it with their team. Complete the Worksheet as a team. To minimize the amount of time you need to spend looking through open windows to find the one you want, it is highly recommended that you close all windows except those needed to complete the activity. The team is expected to work together on each step before moving to the next step of the scenario.  **Hyperlinks:** There are hyperlinks that help drive the scenario, and provide additional information and feedback (please see note on page 1).  **Check on Knowledge Form (Google Form)**: The Check on Knowledge Google Form will ask you to enter team responses. One student should open and manage the google form during the activity. **IMPORTANT**: Once you open the google form, ***keep it open during the entire activity***. If you close the form, the form will take you back to the beginning and you will need to start over.  **Submission**: Download your completed worksheet as a **PDF** and submit it for review. Only one team member/team needs to submit the document. |
| **SCENARIO** |
| Your team has been instructed to operate in MOPP 1 (Mission-Oriented Protective Postures, with some CBRN protective gear worn and some carried) due to intelligence that improvised chemical warfare devices were found locally in your Area of Operation. Every unit member is carrying one nerve agent antidote kit (ATNAA). You are providing medical coverage for a small team that is destroying a weapons cache that was found when clearing a building that your team intended to use as a Casualty Collection Point (CCP). One of the team members was stacking weapons and brushed up against an unidentified liquid.  Optional reference to MOPP 1: Department of Defense. Mission Oriented Protective Posture (MOPP). August 15, 2011. https://media.defense.gov/2012/Jan/13/2000186472/1200/1200/0/120113-F-SU363-001.JPG. Accessed October 14, 2023. |
| 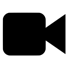 Click here to see what happens next! (Please see Appendix D. Patient Worksheet Video_ Introduction to CBRN Patient)  Image by Kaldari, retrieved from (https://commons.wikimedia.org/wiki/File:Video_Camera_Icon.svg) on October 14, 2023. Creative Commons License associated: https://creativecommons.org/publicdomain/zero/1.0/deed.en |
| **OPTIONAL RESOURCES** |
| - Defense Health Agency. Joint Trauma System Clinical Practice Guideline (CPG) Chemical, Biological Radiological, and Nuclear (CBRN) Injury. Part I: Initial Response to CBRN Agents. May 2018. https://jts.health.mil/assets/docs/cpgs/Chemical_Biological,_Radiological_Nuclear_Injury_Part1_Initial_Response_01_May_2018_ID69.pdf. Accessed October 14, 2023. - Defense Health Agency. Joint Trauma System Clinical Practice Guideline (CPG) Chemical, Biological Radiological, and Nuclear (CBRN) Injury. Part II Chemical, Biological, Radiological and Nuclear (CBRN) Injury Response Part 2: Medical Management of Chemical Agent Exposure. January 2019. https://jts.health.mil/assets/docs/cpgs/Chemical_Biological_Radiological_Nuclear_Injury_Response_Part_2_Medical_Management_25_Mar_2022_ID69.pdf. Accessed October 14, 2023. - Lecture Slides: Appendix A, author owned. - DeFeo DR, Givens ML. Integrating Chemical Biological, Radiologic, and Nuclear (CBRN) Protocols Into TCCC Introduction of a Conceptual Model - TCCC + CBRN = (MARCHE)2. J Spec Oper Med. 2018;18(1):118-123. doi:10.55460/ZK2U-M1DZ - Ciottone GR. Toxidrome Recognition in Chemical-Weapons Attacks. N Engl J Med. 2018;378(17):1611-1620. doi:10.1056/NEJMra1705224 - Henretig FM, Kirk MA, McKay CA Jr. Hazardous Chemical Emergencies and Poisonings. N Engl J Med. 2019;380(17):1638-1655. doi:10.1056/NEJMra1504690 - United States Army Combined Arms Center. GTA 03-08-002 Contaminated Casualty Care. January 23, 2017. https://usacac.army.mil/organizations/mccoe/call/publication/GTA_03-08-002. Accessed October 14, 2023. - Jones SL, Walsh RS, Stearney SA, Allen R. Multi-service Tactics, Techniques, and Procedures for Health Service Support in a Chemical, Biological, Radiological and Nuclear Environment. Army Publishing Directorate. March 2016. https://armypubs.army.mil/epubs/DR_pubs/DR_a/pdf/web/atp4_02x7.pdf. Accessed October 14, 2023. |
| **PART I: Introduction to the Patient** |
| 1. This patient was treated as a suspected nerve agent. What are some other possible causes for this patient’s symptoms? |
| 2. What features on history or physical exam would help you determine the most likely etiology of the patient’s symptoms? |
| 3. If you had received the call made to the medic, what would be your immediate instructions for this patient? Be specific in the instructions and be able to explain to the patient. Justify instructions and be able to explain the intent and intended outcomes. |
| 4. What other actions would you take as the medical leader? |
| 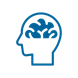 **Part I Check on Knowledge:** Go to the Check on Knowledge google form (Appendix G) to answer questions 1-3 and receive Part I feedback (Appendix H).  Image by AomAm, retrieved from (https://commons.wikimedia.org/wiki/File:Brain_icon_from_Noun_Project.png) on October 14, 2023. Creative Commons License associated: https://creativecommons.org/licenses/by/3.0/deed.en |
| Place your cursor in the row below; click on the “Background Color” icon above and change to “no color” to reveal a team task. |
| Have one team member role play an **UNCONSCIOUS** patient; the other team members must don the mask onto the unconscious patient. |
| **Part II: Corpsman Response** |
| 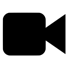 Click here to see what happens next! (Please see Appendix E. Patient Worksheet Video_ CBRN Corpsman Response)  Image by Kaldari, retrieved from (https://commons.wikimedia.org/wiki/File:Video_Camera_Icon.svg) on October 14, 2023. Creative Commons License associated: https://creativecommons.org/publicdomain/zero/1.0/deed.en |
| 1. Using MARCHE(2) what were the immediate actions/instructions for the medic responding to this patient? What other measures could have been taken? |
| 2. What are your concerns for this patient? |
| 3. Based on the wind direction at your proposed site below, how would you orient the Patient Decontamination Site (PDS) given the potential for contaminants to travel downwind? Recall that patient decontamination occurs in the warm zone.  Optional reference to PDS: United States Army Combined Arms Center. GTA 03-08-002 Contaminated Casualty Care. January 23, 2017. https://usacac.army.mil/organizations/mccoe/call/publication/GTA_03-08-002. Accessed October 14, 2023.  Drag and drop the “Drop Off Point” icon onto the graphic below to display your site orientation. |
| 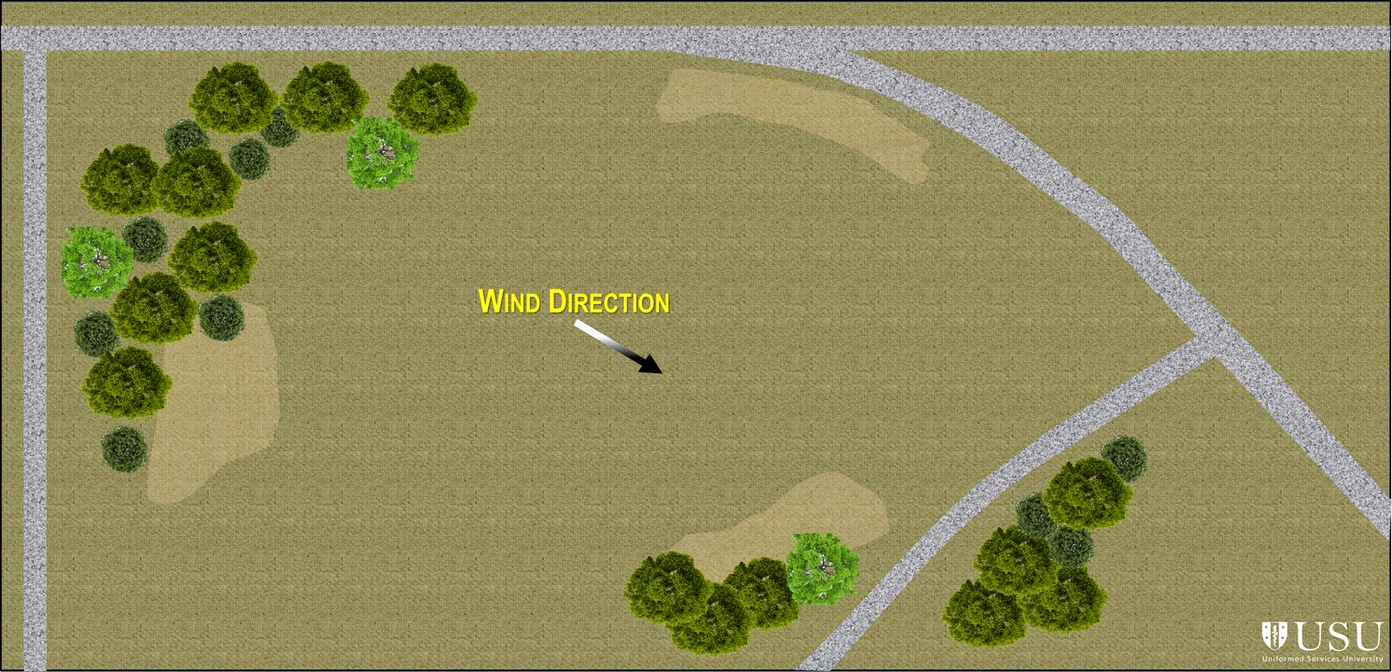  Image is author owned. |
| 4. If this patient needed an immediate lifesaving intervention at the PDS drop off point, where would this patient be moved to within the PDS once triaged? |
| 5. Describe the mechanism of action of nerve agents and how this relates to history and physical exam findings. |
| 6. What further history would you like to obtain? How could you obtain further history if the patient is altered? |
| 7. What physical exam findings would help you in narrowing your differential diagnosis? |
| 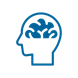 **Part II Check on Knowledge:** Go to the Check on Knowledge google form (Appendix G) to answer questions 4-5 and receive Part II feedback (Appendix H).  Image by AomAm, retrieved from (https://commons.wikimedia.org/wiki/File:Brain_icon_from_Noun_Project.png) on October 14, 2023. Creative Commons License associated: https://creativecommons.org/licenses/by/3.0/deed.en |
| **Part III: Physician Response** |
| The patient is decontaminated and brought to your vehicle where you have established a hasty aid station. Your aid station is considered a clean area. The patient has been fully decontaminated and cut out of their protective gear and clothing. The patient was washed down with water and reactive skin decontamination lotion (RSDL) was reapplied to the area where their arm was exposed to the liquid. The patient continues to have an altered mental status and copious secretions.  Optional reference to RSDL: Jones SL, Walsh RS, Stearney SA, Allen R. Multi-service Tactics, Techniques, and Procedures for Health Service Support in a Chemical, Biological, Radiological and Nuclear Environment. Army Publishing Directorate. March 2016. https://armypubs.army.mil/epubs/DR_pubs/DR_a/pdf/web/atp4_02x7.pdf. Accessed October 14, 2023. |
| 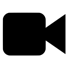 Click here to see what happens next! (Please see Appendix F. Patient Worksheet Video_ Physician Assessment)  Image by Kaldari, retrieved from (https://commons.wikimedia.org/wiki/File:Video_Camera_Icon.svg) on October 14, 2023. Creative Commons License associated: https://creativecommons.org/publicdomain/zero/1.0/deed.en |
| Place your cursor in the row below; click on the “Background Color” icon above and change to “no color” to reveal the patient’s vital signs and full physical exam. |
| ➔ BP 148/92, HR 110, RR 24, SpO2 89%  ➔ Gen: post ictal and beginning to respond to stimuli  ➔ HEENT: + lacrimation, + rhinorrhea, +miosis, pooling secretions in oropharynx with noisy respirations  ➔ Neck: no JVD  ➔ CV: tachycardia, regular no m/r/g  ➔ Lungs: bilateral rhonchi and rales  ➔ Abd: increased BS, non-tender  ➔ Extremities: local fasciculations on arm, no rash, + diaphoresis |
| 1. What are your actions upon his arrival to the aid station? |
| 2. Based on your reassessment, you note the patient is in respiratory distress. Describe your actions to manage this patient’s airway and respiratory status. |
| 3. Is it appropriate to give atropine to this patient with tachycardia? Why or why not? |
| 4. The patient’s mental status continues to improve, but he is not yet fully alert. Does this patient require additional benzodiazepines? Describe how benzodiazepines work to stop seizures. |
| 5. Consider the discussion points below:  How might RSI mask the ability to note seizure activity?  How does administration of benzodiazepines affect your ability to assess patient mental status? |
| 6. What vital sign was omitted in the reassessment? |
| 7. What are the considerations for body temperature in this patient? |
| 8. What are the evacuation considerations for this patient? |
| 9. What *evacuation* category is this patient? |
| 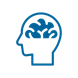 **Part III Check on Knowledge:** Go to the Check on Knowledge google form (Appendix G) to answer questions 6-8 and receive Part III feedback (Appendix H).  Image by AomAm, retrieved from (https://commons.wikimedia.org/wiki/File:Brain_icon_from_Noun_Project.png) on October 14, 2023. Creative Commons License associated: https://creativecommons.org/licenses/by/3.0/deed.en |
| Consider how this case would have progressed differently if the patient suffered a GSW to the arm that resulted in a JLIST suit breach and exposure. |
| 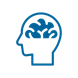 Go to the **Check on Knowledge Google form** to complete the end of lesson student survey (Appendix J).  Image by AomAm, retrieved from (https://commons.wikimedia.org/wiki/File:Brain_icon_from_Noun_Project.png) on October 14, 2023. Creative Commons License associated: https://creativecommons.org/licenses/by/3.0/deed.en |
