# Supplementary material for: Introduction to Treating Patients Exposed to Chemical, Biological, Radiological, and Nuclear (CBRN) Threats: A Military Medical Case-Based Curriculum
Source: MedEdPORTAL. 2024 Sep 13;20:11433. doi: 10.15766/mep_2374-8265.11433 (PMC11393073; doi:10.15766/mep_2374-8265.11433)
Supplement: Supplementary file 1 — Session One Lecture.pptxSupplemental Resources for Session One.docxCBRN Patient Worksheet.docxPatient Worksheet Video - Introduction to CBRN Patient.mp4Patient Worksheet Video - CBRN Corpsman Response.mp4Patient Worksheet Video - Physician Assessment.mp4Check on Knowledge Form.docxCBRN Patient Worksheet - Facilitator Version.docxFacilitator Guide.docxStudent Survey.docxSupplemental Resources for Session Two.docx [file mep_2374-8265.11433-s001.zip › G. Check on Knowledge Form.docx]

**Appendix G. Check on Knowledge Form**

The following Check on Knowledge Form was developed using Google Forms platform and integrated with the Student Survey (Appendix J) to facilitate completion. The students opened this form and kept it open throughout the CBRN Patient Worksheet completion, returning to the form at the conclusion of each section to answer questions and receive immediate feedback upon submission of their selections (Appendix H).

Check on Knowledge Form

1. Identify your team members:

PART I - Introduction to the Patient

1. What was the first immediate instruction given to the patient?
2. Name one additional action you would take or recommend as a medical leader?

See Part I Immediate Feedback on page 6.

Do not proceed to the next section unless directed to on the CBRN Patient Worksheet.

PART II – Corpsman Response

1. Using MARCHE(2), what was the immediate action/instruction provided by the corpsman in the video responding to this patient (mark only one option)?
   - Administering ATNAA/CANA
   - Evacuating from site
   - Securing the chemical mask
   - Securing the chemical suit
2. According to the GTA 03-08-002 JAN 2017, Contaminated Casualty Care, which statement(s) are TRUE for initial wound decontamination (select all that apply)?
   - During thorough patient decontamination, all bandages suspected of contamination are removed and the wounds are flushed with isotonic saline solution or water.
   - Bandages are replaced only if bleeding begins after decontamination.
   - Both bandage replacement and tourniquet replacement are performed by medical personnel in the warm zone before transferring to the cold zone.
   - Tourniquets suspected of being contaminated are replaced with clean tourniquets, and the sites of the original tourniquets are decontaminated.
   - Splints are thoroughly decontaminated but removed only by a medic or under a physician’s supervision.

**Reference:**

United States Army Combined Arms Center. GTA 03-08-002 Contaminated Casualty Care. January 23, 2017. https://usacac.army.mil/organizations/mccoe/call/publication/GTA_03-08-002. Accessed February 8, 2023.

See Part II Immediate Feedback on page 8.

Do not proceed to the next section unless directed to on the CBRN Patient Worksheet.

PART III – Physician Response

1. What was the physician's immediate action upon the patient's arrival to the aid station (mark only one option)?
   - Decontamination of the patient
   - Immediate call for an NPA and Suction
   - Primary Assessment
   - Secondary Assessment
2. According to the Multi-service Tactics, Techniques, and Procedures for Health Service Support in a Chemical, Biological, Radiological and Nuclear Environment, which statement(s) below are TRUE for Reactive Skin Decontamination Lotion (RSDL) (select all that apply)?
   - The RSDL can be used for the decontamination of intact skin around wounds, but is not approved for the decontamination of open wounds.
   - RSDL is safe to be left on the skin for up to 24 hours.
   - RSDL should remain in contact with the skin for at least two minutes and then be removed with soap and water when conditions permit.
   - Basis of allocation of RSDL is two packets per individual.
3. Provide two evacuation considerations for this patient.

**Reference**

Jones SL, Walsh RS, Stearney SA, Allen R. Multi-service Tactics, Techniques, and Procedures for Health Service Support in a Chemical, Biological, Radiological and Nuclear Environment. Army Publishing Directorate. March 2016. https://armypubs.army.mil/epubs/DR_pubs/DR_a/pdf/web/atp4_02x7.pdf. Accessed October 14, 2023.

See Part III Immediate Feedback on page 11.

Do not proceed to the next section until directed to complete the end of lesson formative assessment.
