# Supplementary material for: Introduction to Treating Patients Exposed to Chemical, Biological, Radiological, and Nuclear (CBRN) Threats: A Military Medical Case-Based Curriculum
Source: MedEdPORTAL. 2024 Sep 13;20:11433. doi: 10.15766/mep_2374-8265.11433 (PMC11393073; doi:10.15766/mep_2374-8265.11433)
Supplement: Supplementary file 1 — Session One Lecture.pptxSupplemental Resources for Session One.docxCBRN Patient Worksheet.docxPatient Worksheet Video - Introduction to CBRN Patient.mp4Patient Worksheet Video - CBRN Corpsman Response.mp4Patient Worksheet Video - Physician Assessment.mp4Check on Knowledge Form.docxCBRN Patient Worksheet - Facilitator Version.docxFacilitator Guide.docxStudent Survey.docxSupplemental Resources for Session Two.docx [file mep_2374-8265.11433-s001.zip › H. CBRN Patient Worksheet - Facilitator Version.docx]

**Appendix H. CBRN Patient Worksheet: Facilitator's Version**

This is the facilitator’s version of the CBRN Patient Worksheet (Appendix C) with answers embedded within the worksheet. This document also includes the answers and immediate feedback for each question on the Check on Knowledge Form (Appendix G), which was developed on the Google Forms platform (please see pages 12-13 below). The immediate feedback was integrated as embedded links within the Check on Knowledge Google Form.

As indicated above, the worksheet was originally formatted for distribution to students with embedded hyperlinks (indicated in blue and underlined) for ease of access during the activity. It is meant to be completed as a student group, with each group filling out one worksheet. Time available for completion is 60-90 minutes.

Given its current distribution as a reusable educational material, the hyperlinks have been removed from the document and replaced with references to optional supporting materials.

For your reference, the Check on Knowledge Form is available in Appendix G. The Student Survey is available in Appendix J. The videos are available in Appendices D, E, and F, as noted within the worksheet.

All facilitator information to include answers, notes, and pertinent references are in red text.

| INTRODUCTION TO THE CBRN PATIENT CASE SCENARIO |
| --- |
| **OBJECTIVES** |
| - Employ the CRESS algorithm to rapidly evaluate for potential chemical agent exposure - Develop an organized approach to the evaluation and treatment of a CBRN patient using principles of (MARCHE)2 - Compare and contrast the presenting signs and symptoms of nerve and pulmonary agents - Describe the mechanism of action for nerve agents and how this informs treatment of these patients |
| **INSTRUCTIONS** |
| **Pre-work:** Make sure you have read the JTS Clinical Practice Guidelines prior to beginning the worksheet.  **Getting Started**: One team member needs to make a copy of this worksheet on the Google drive platform, rename it (Company-Platoon-Fire Team, i.e. A-1-1a), and share it with their team. Complete the Worksheet as a team. To minimize the amount of time you need to spend looking through open windows to find the one you want, it is highly recommended that you close all windows except those needed to complete the activity. The team is expected to work together on each step before moving to the next step of the scenario.  **Hyperlinks:** There are hyperlinks that help drive the scenario, and provide additional information and feedback (please see note on page 1).  **Check on Knowledge Form (Google Form)**: The Check on Knowledge Google Form will ask you to enter team responses. One student should open and manage the google form during the activity. **IMPORTANT**: Once you open the google form, ***keep it open during the entire activity***. If you close the form, the form will take you back to the beginning and you will need to start over.  **Submission**: Download your completed worksheet as a **PDF** and submit it for review. Only one team member/team needs to submit the document. |
| **SCENARIO** |
| Your team has been instructed to operate in MOPP 1 (Mission-Oriented Protective Postures, with some CBRN protective gear worn and some carried) due to intelligence that improvised chemical warfare devices were found locally in your Area of Operation. Every unit member is carrying one nerve agent antidote kit (ATNAA). You are providing medical coverage for a small team that is destroying a weapons cache that was found when clearing a building that your team intended to use as a Casualty Collection Point (CCP). One of the team members was stacking weapons and brushed up against an unidentified liquid.  Optional reference to MOPP 1: Department of Defense. Mission Oriented Protective Posture (MOPP). August 15, 2011. https://media.defense.gov/2012/Jan/13/2000186472/1200/1200/0/120113-F-SU363-001.JPG. Accessed October 14, 2023. |
| 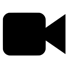 Click here to see what happens next! (Please see Appendix D. Patient Worksheet Video_ Introduction to CBRN Patient)  Image by Kaldari, retrieved from (https://commons.wikimedia.org/wiki/File:Video_Camera_Icon.svg) on October 14, 2023. Creative Commons License associated: https://creativecommons.org/publicdomain/zero/1.0/deed.en |
| **OPTIONAL RESOURCES** |
| - Defense Health Agency. Joint Trauma System Clinical Practice Guideline (CPG) Chemical, Biological Radiological, and Nuclear (CBRN) Injury. Part I: Initial Response to CBRN Agents. May 2018. https://jts.health.mil/assets/docs/cpgs/Chemical_Biological,_Radiological_Nuclear_Injury_Part1_Initial_Response_01_May_2018_ID69.pdf. Accessed October 14, 2023. - Defense Health Agency. Joint Trauma System Clinical Practice Guideline (CPG) Chemical, Biological Radiological, and Nuclear (CBRN) Injury. Part II Chemical, Biological, Radiological and Nuclear (CBRN) Injury Response Part 2: Medical Management of Chemical Agent Exposure. January 2019. https://jts.health.mil/assets/docs/cpgs/Chemical_Biological_Radiological_Nuclear_Injury_Response_Part_2_Medical_Management_25_Mar_2022_ID69.pdf. Accessed October 14, 2023. - Lecture Slides: Appendix A, author owned. - DeFeo DR, Givens ML. Integrating Chemical Biological, Radiologic, and Nuclear (CBRN) Protocols Into TCCC Introduction of a Conceptual Model - TCCC + CBRN = (MARCHE)2. J Spec Oper Med. 2018;18(1):118-123. doi:10.55460/ZK2U-M1DZ - Ciottone GR. Toxidrome Recognition in Chemical-Weapons Attacks. N Engl J Med. 2018;378(17):1611-1620. doi:10.1056/NEJMra1705224 - Henretig FM, Kirk MA, McKay CA Jr. Hazardous Chemical Emergencies and Poisonings. N Engl J Med. 2019;380(17):1638-1655. doi:10.1056/NEJMra1504690 - United States Army Combined Arms Center. GTA 03-08-002 Contaminated Casualty Care. January 23, 2017. https://usacac.army.mil/organizations/mccoe/call/publication/GTA_03-08-002. Accessed October 14, 2023. - Jones SL, Walsh RS, Stearney SA, Allen R. Multi-service Tactics, Techniques, and Procedures for Health Service Support in a Chemical, Biological, Radiological and Nuclear Environment. Army Publishing Directorate. March 2016. https://armypubs.army.mil/epubs/DR_pubs/DR_a/pdf/web/atp4_02x7.pdf. Accessed October 14, 2023. |
| **PART I: Introduction to the Patient** |
| 1. This patient was treated as a suspected nerve agent. What are some other possible causes for this patient’s symptoms?   - Exposure to chemical agent - Viral illness - Acute coronary syndrome - Gastrointestinal disease - Exertional heat injury |
| 2. What features on history or physical exam would help you determine the most likely etiology of the patient’s symptoms?   - Note immediacy of symptoms relative to the contact with the unknown liquid - Patient is otherwise healthy young male – low risk for CVD - If asked on further history, other unit members all ate same food and none are ill - No recent ill contacts - Patient also has rhinorrhea, lacrimation, and noticed fasciculations near exposure site if queried   **Reference for Questions 1 & 2:**  Defense Health Agency. Joint Trauma System Clinical Practice Guideline (CPG) Chemical, Biological Radiological, and Nuclear (CBRN) Injury. Part I: Initial Response to CBRN Agents. May 2018. https://jts.health.mil/assets/docs/cpgs/Chemical_Biological,_Radiological_Nuclear_Injury_Part1_Initial_Response_01_May_2018_ID69.pdf. Accessed February 8, 2023.  See “Table 2. Chemical casualty assessment” on page 9.  **Facilitator Notes**:  CBRN casualties can be categorized by circumstances of exposure and presence/absence of CBRN effects. CRESS (Consciousness, Respirations, Eyes, Secretions, Skin) is a tool to rapidly evaluate for CBRN injuries. It can be used to identify/categorize CBRN injury and improve rapid identification of the type of chemical agent exposure. Casualty treatment should never be delayed pending confirmatory agent identification. Clinical assessment is necessary to determine immediate therapy. |
| 3. If you had received the call made to the medic, what would be your immediate instructions for this patient? Be specific in the instructions and be able to explain to the patient. Justify instructions and be able to explain the intent and intended outcomes.   - Immediately don protective mask to limit exposure - Self-administer ATNAA/CANA – need for immediate treatment to prevent irreversible aging of nerve agent. Note that in this scenario, only ATNAA was self-administered. - Apply RSDL to site of exposure – helps to neutralize the agent - Egress from exposure site – again, the goal is to limit exposure   **Reference for Question 3:**  Defense Health Agency. Joint Trauma System Clinical Practice Guideline (CPG) Chemical, Biological Radiological, and Nuclear (CBRN) Injury. Part I: Initial Response to CBRN Agents. May 2018. https://jts.health.mil/assets/docs/cpgs/Chemical_Biological,_Radiological_Nuclear_Injury_Part1_Initial_Response_01_May_2018_ID69.pdf. Accessed February 8, 2023.  See “Step 1: Hot Zone / Care Under Fire” on page 13.  **Facilitator Notes**:  Direct patient to perform appropriate self-aid measures for agent of concern based on CRESS symptoms identified (Nerve Agent). |
| 4. What other actions would you take as the medical leader?   - Advise command to assume MOPP 4 in the immediate vicinity - Advise evacuation of all unit members from the building and immediate vicinity - Establish a hot line and coordinate a decontamination station - Communicate in proper channels   **Reference for Question 4:**  Defense Health Agency. Joint Trauma System Clinical Practice Guideline (CPG) Chemical, Biological Radiological, and Nuclear (CBRN) Injury. Part I: Initial Response to CBRN Agents. May 2018. https://jts.health.mil/assets/docs/cpgs/Chemical_Biological,_Radiological_Nuclear_Injury_Part1_Initial_Response_01_May_2018_ID69.pdf. Accessed February 8, 2023.  See “Critical Task List” on page 3. |
| 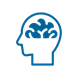 **Part I Check on Knowledge:** Go to the Check on Knowledge google form (Appendix G) to answer questions 1-3 and receive Part I feedback (feedback available at conclusion of this document).  Image by AomAm, retrieved from (https://commons.wikimedia.org/wiki/File:Brain_icon_from_Noun_Project.png) on October 14, 2023. Creative Commons License associated: https://creativecommons.org/licenses/by/3.0/deed.en |
| Place your cursor in the row below; click on the “Background Color” icon above and change to “no color” to reveal a team task. |
| Have one team member role play an **UNCONSCIOUS** patient; the other team members must don the mask onto the unconscious patient. |
| **Part II: Corpsman Response** |
| 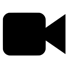 Click here to see what happens next! (Please see Appendix E. Patient Worksheet Video_ CBRN Corpsman Response)  Image by Kaldari, retrieved from (https://commons.wikimedia.org/wiki/File:Video_Camera_Icon.svg) on October 14, 2023. Creative Commons License associated: https://creativecommons.org/publicdomain/zero/1.0/deed.en |
| 1. Using MARCHE(2) what were the immediate actions/instructions for the medic responding to this patient? What other measures could have been taken?   - Ensure medical responders are in adequate protective gear - Ensure patient’s mask is on with an appropriate seal - Assess response to antidote and administer ATNAA/CANA - Apply RSDL to the site of contamination   **Reference for Question 1:**  Defense Health Agency. Joint Trauma System Clinical Practice Guideline (CPG) Chemical, Biological Radiological, and Nuclear (CBRN) Injury. Part I: Initial Response to CBRN Agents. May 2018. https://jts.health.mil/assets/docs/cpgs/Chemical_Biological,_Radiological_Nuclear_Injury_Part1_Initial_Response_01_May_2018_ID69.pdf. Accessed February 8, 2023.  See “Step 1: Hot Zone / Care Under Fire” on page 13.  **Facilitator Notes**:  Direct patient to perform appropriate self-aid measures for agents of concern based on CRESS symptoms identified (Nerve Agent). |
| 2. What are your concerns for this patient?   - - Airway management   - Increased support requirement for altered patient who is non-ambulatory   - Prepare for recurrent seizures   - Continue to consider differential diagnosis while treating for nerve agent exposure and note altered mental status |
| 3. Based on the wind direction at your proposed site below, how would you orient the Patient Decontamination Site (PDS) given the potential for contaminants to travel downwind? Recall that patient decontamination occurs in the warm zone.  Optional reference to PDS: United States Army Combined Arms Center. GTA 03-08-002 Contaminated Casualty Care. January 23, 2017. https://usacac.army.mil/organizations/mccoe/call/publication/GTA_03-08-002. Accessed October 14, 2023.  Drag and drop the “Drop Off Point” icon onto the graphic below to display your site orientation. |
| 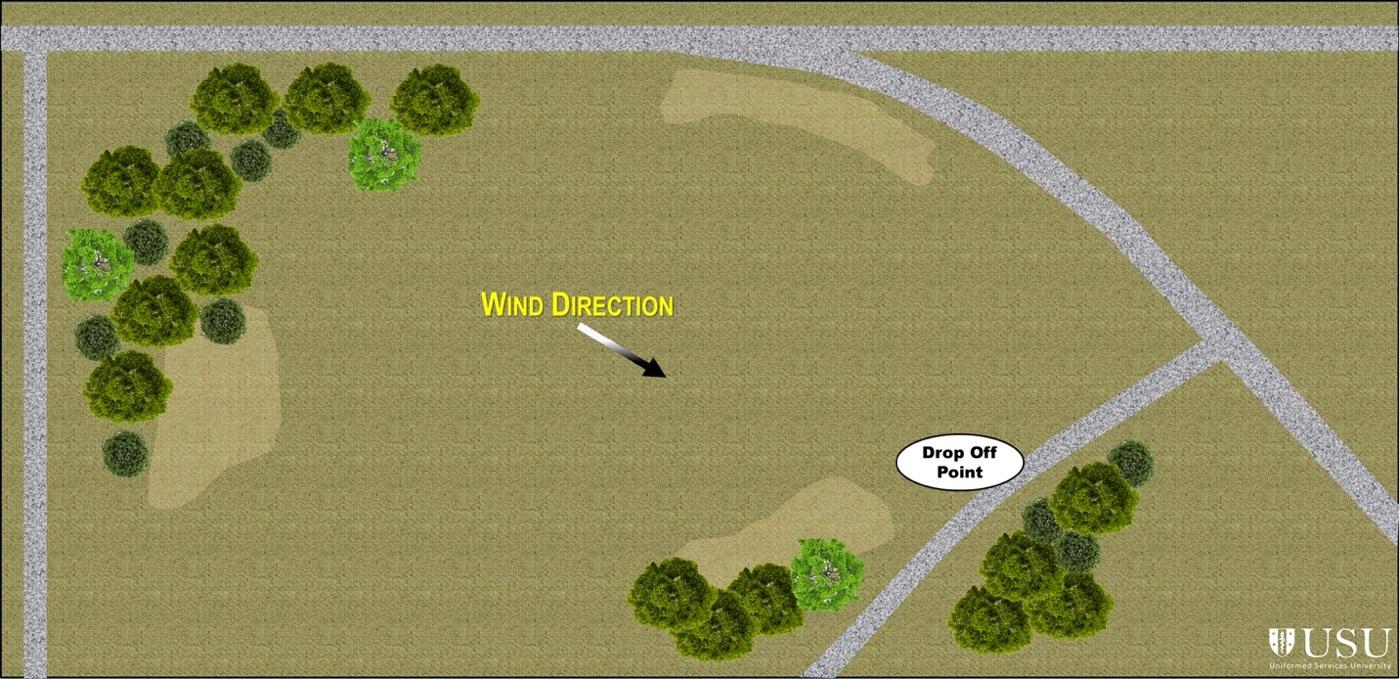  Image is author owned. |
| 4. If this patient needed an immediate lifesaving intervention at the PDS drop off point, where would this patient be moved to within the PDS once triaged?   - - Emergency Medical Treatment (Warm Side) |
| 5. Describe the mechanism of action of nerve agents and how this relates to history and physical exam findings.   - - Nerve agents bind to acetylcholinesterase which results in excess acetylcholine and subsequent cholinergic toxidrome (diarrhea, urination, miosis, bradycardia, bronchorrhea, emesis, lacrimation, salivation) and nicotinic findings (mydriasis, tachycardia, weakness, hypertension, fasciculations). Differentiation between nerve agent and pulmonary agent is important. Pulmonary agent exposure can also result in lacrimation, salivation, rhinorrhea, and respiratory distress. Pulmonary agents’ effects are typically limited to their irritant effect (lacrimation, rhinorrhea, bronchospasm) and damage to the respiratory tract. Cholinergic findings such as bradycardia, sweating, diarrhea, and urination can help differentiate. Miosis, when present, can be very helpful to confirm nerve agent exposure, but it is not always present with small skin exposures. Seizures and fasciculations are findings that are highly suggestive of nerve agent.   **References for Question 5:**  Defense Health Agency. Joint Trauma System Clinical Practice Guideline (CPG) Chemical, Biological Radiological, and Nuclear (CBRN) Injury. Part II Chemical, Biological, Radiological and Nuclear (CBRN) Injury Response Part 2: Medical Management of Chemical Agent Exposure. January 2019. https://jts.health.mil/assets/docs/cpgs/Chemical_Biological_Radiological_Nuclear_Injury_Response_Part_2_Medical_Management_25_Mar_2022_ID69.pdf. Accessed February 8, 2023.  See “Nerve Agent Exposure” on page 7.  Henretig FM, Kirk MA, McKay CA Jr. Hazardous Chemical Emergencies and Poisonings. *N Engl J Med*. 2019;380(17):1638-1655. doi:10.1056/NEJMra1504690  See figure 2 “organophosphate poisoning” on page 1648. |
| 6. What further history would you like to obtain? How could you obtain further history if the patient is altered?   - - Obtain history from patient’s team members   - No other team members were in the same room or came in contact with the liquid (per team leader)   - The patient has no other medical problems, no medications, and no allergies (per team medic) |
| 7. What physical exam findings would help you in narrowing your differential diagnosis?   - - The presence of miosis in the setting of increased secretions is highly suggestive of nerve agent. Fasciculations are a manifestation of the nicotinic effects of acetylcholine excess. These findings can help differentiate from a pulmonary irritant which may also cause increased secretions, respiratory symptoms, and tachycardia. Note that miosis is not universally present in nerve agent exposures and the absence of miosis should not preclude treatment.   **Reference for Question 7:**  Defense Health Agency. Joint Trauma System Clinical Practice Guideline (CPG) Chemical, Biological Radiological, and Nuclear (CBRN) Injury. Part I: Initial Response to CBRN Agents. May 2018. https://jts.health.mil/assets/docs/cpgs/Chemical_Biological,_Radiological_Nuclear_Injury_Part1_Initial_Response_01_May_2018_ID69.pdf. Accessed February 8, 2023.  See “Table 2. Chemical casualty assessment” on page 9.  **Facilitator Notes:**  CBRN casualties can be categorized by circumstances of exposure and presence/absence of CBRN effects. CRESS (Consciousness, Respirations, Eyes, Secretions, Skin) is a tool to rapidly evaluate for CBRN injuries. It can be used to rule out or rule in CBRN injury and improve rapid identification of the type of chemical agent exposure. Casualty treatment should never be delayed pending confirmatory agent identification. Clinical assessment is necessary to determine immediate therapy. |
| 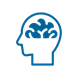 **Part II Check on Knowledge:** Go to the Check on Knowledge google form (Appendix G) to answer questions 4-5 and receive Part II feedback (feedback available at conclusion of this document).  Image by AomAm, retrieved from (https://commons.wikimedia.org/wiki/File:Brain_icon_from_Noun_Project.png) on October 14, 2023. Creative Commons License associated: https://creativecommons.org/licenses/by/3.0/deed.en |
| **Part III: Physician Response** |
| The patient is decontaminated and brought to your vehicle where you have established a hasty aid station. Your aid station is considered a clean area. The patient has been fully decontaminated and cut out of their protective gear and clothing. The patient was washed down with water and reactive skin decontamination lotion (RSDL) was reapplied to the area where their arm was exposed to the liquid. The patient continues to have an altered mental status and copious secretions.  Optional reference to RSDL: Jones SL, Walsh RS, Stearney SA, Allen R. Multi-service Tactics, Techniques, and Procedures for Health Service Support in a Chemical, Biological, Radiological and Nuclear Environment. Army Publishing Directorate. March 2016. https://armypubs.army.mil/epubs/DR_pubs/DR_a/pdf/web/atp4_02x7.pdf. Accessed October 14, 2023. |
| 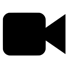 Click here to see what happens next! (Please see Appendix F. Patient Worksheet Video_ Physician Assessment)  Image by Kaldari, retrieved from (https://commons.wikimedia.org/wiki/File:Video_Camera_Icon.svg) on October 14, 2023. Creative Commons License associated: https://creativecommons.org/publicdomain/zero/1.0/deed.en |
| Place your cursor in the row below; click on the “Background Color” icon above and change to “no color” to reveal the patient’s vital signs and full physical exam. |
| ➔ BP 148/92, HR 110, RR 24, SpO2 89%  ➔ Gen: post ictal and beginning to respond to stimuli  ➔ HEENT: + lacrimation, + rhinorrhea, +miosis, pooling secretions in oropharynx with noisy respirations  ➔ Neck: no JVD  ➔ CV: tachycardia, regular no m/r/g  ➔ Lungs: bilateral rhonchi and rales  ➔ Abd: increased BS, non-tender  ➔ Extremities: local fasciculations on arm, no rash, + diaphoresis |
| 1. What are your actions upon his arrival to the aid station?   - - Immediate action is to reassess the patient |
| 2. Based on your reassessment, you note the patient is in respiratory distress. Describe your actions to manage this patient’s airway and respiratory status.   - - This patient is still showing cholinergic symptoms, so it is appropriate to give more anticholinergic treatment (atropine or scopolamine). IV/IO access should be established. Treatment should continue until respiratory secretions resolve. Patient positioning to support the airway along with suctioning and clearing the airway of secretions should be done in conjunction with medication. Additional dosing of pralidoxime is appropriate. For this scenario, we can discuss pros/cons of intubation relative to equipment and resources. Is oxygen available? Is a ventilator available? |
| 3. Is it appropriate to give atropine to this patient with tachycardia? Why or why not?   - - Atropine is often used to treat bradycardia as its anticholinergic effects block the parasympathetic effects of the vagus nerve, resulting in increased heart rate. It is likely that this patient’s tachycardia is due to respiratory distress and, therefore, treating the respiratory symptoms would likely lead to improvement in the heart rate. Atropine should not be withheld in the setting of respiratory symptoms with tachycardia. |
| 4. The patient’s mental status continues to improve, but he is not yet fully alert. Does this patient require additional benzodiazepines? Describe how benzodiazepines work to stop seizures.   - - The patient has altered mental status which could be post-ictal from the seizure or direct effects of the nerve agent, but it is important to continue to monitor neurological status to ensure recovery or identification of other causes for AMS.   - Benzodiazepines are GABA agonists. GABA is an inhibitory neurotransmitter that typically terminates seizures by countering neuronal excitation.   - Further use of benzodiazepines for seizure control is not indicated in the absence of seizure activity AND improving mental status. However, if fasciculations are painful or diffuse, benzodiazepines may provide relief.   **Reference for Question 4:**  Defense Health Agency. Joint Trauma System Clinical Practice Guideline (CPG) Chemical, Biological Radiological, and Nuclear (CBRN) Injury. Part II Chemical, Biological, Radiological and Nuclear (CBRN) Injury Response Part 2: Medical Management of Chemical Agent Exposure. January 2019. https://jts.health.mil/assets/docs/cpgs/Chemical_Biological_Radiological_Nuclear_Injury_Response_Part_2_Medical_Management_25_Mar_2022_ID69.pdf. Accessed February 8, 2023.  See “Nerve Agent Exposure” on page 7. |
| 5. Consider the discussion points below:  How might RSI mask the ability to note seizure activity?   - - Using a paralytic agent would mask motor activity associated with seizures. Even though there is no motor activity, there could still be seizure activity in the brain.   How does administration of benzodiazepines affect your ability to assess patient mental status?   - - Benzodiazepines have sedating effects and cause depressed level of consciousness. |
| 6. What vital sign was omitted in the reassessment?   - - Temperature. |
| 7. What are the considerations for body temperature in this patient?   - - Working in protective gear can cause elevated body temperature and predispose to heat injury.   - Seizure activity could further exacerbate elevation in core body temperature.   - Decontamination could result in hypothermia due to removal of protective clothing with subsequent convection and conduction losses to the environment, and heat loss due to evaporation when using water for decontamination.   - Appropriate measures should be taken to either facilitate return to normothermia or prevent heat losses that would result in hypothermia.   **Reference for Question 7:**  Defense Health Agency. Joint Trauma System Clinical Practice Guideline (CPG) Chemical, Biological Radiological, and Nuclear (CBRN) Injury. Part I: Initial Response to CBRN Agents. May 2018. https://jts.health.mil/assets/docs/cpgs/Chemical_Biological,_Radiological_Nuclear_Injury_Part1_Initial_Response_01_May_2018_ID69.pdf. Accessed February 8, 2023.  See “Step 2: Warm Zone / Tactical Field Care” on page 14. |
| 8. What are the evacuation considerations for this patient?   - - Prepare to handle respiratory decompensation, recurrence of seizures, ongoing neurologic assessment, and maintain body temperature. |
| 9. What *evacuation* category is this patient?   - - Urgent (could also argue priority if respiratory status is stable and mental status improving)   **Reference for Question 9:**  Defense Health Agency. Joint Trauma System Clinical Practice Guideline (CPG) Chemical, Biological Radiological, and Nuclear (CBRN) Injury. Part I: Initial Response to CBRN Agents. May 2018. https://jts.health.mil/assets/docs/cpgs/Chemical_Biological,_Radiological_Nuclear_Injury_Part1_Initial_Response_01_May_2018_ID69.pdf. Accessed February 8, 2023.  See “Step 1: Hot Zone / Care Under Fire” on page 13. |
| 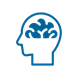 **Part III Check on Knowledge:** Go to the Check on Knowledge google form (Appendix G) to answer questions 6-8 and receive Part III feedback (feedback available at conclusion of this document).  Image by AomAm, retrieved from (https://commons.wikimedia.org/wiki/File:Brain_icon_from_Noun_Project.png) on October 14, 2023. Creative Commons License associated: https://creativecommons.org/licenses/by/3.0/deed.en |
| Consider how this case would have progressed differently if the patient suffered a GSW to the arm that resulted in a JLIST suit breach and exposure.  **Facilitator Notes:**  Considerations include addressing immediate life threats first, such as massive hemorrhage. Control of the hemorrhage, per TCCC guidelines, would take priority over CBRN measures. Also note that breach of the suit would require rapid decontamination with a potential need to replace contaminated tourniquets and bandages in the warm zone if bleeding is not controlled. Consider whether airway or breathing interventions can be delayed until after mask application.  **Reference:**  Defense Health Agency. Joint Trauma System Clinical Practice Guideline (CPG) Chemical, Biological Radiological, and Nuclear (CBRN) Injury. Part I: Initial Response to CBRN Agents. May 2018. https://jts.health.mil/assets/docs/cpgs/Chemical_Biological,_Radiological_Nuclear_Injury_Part1_Initial_Response_01_May_2018_ID69.pdf. Accessed February 8, 2023.  See “Step 1: Hot Zone / Care Under Fire” on page 13. |
| 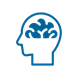 Go to the **Check on Knowledge Google form** to complete the end of lesson student survey (Appendix J).  Image by AomAm, retrieved from (https://commons.wikimedia.org/wiki/File:Brain_icon_from_Noun_Project.png) on October 14, 2023. Creative Commons License associated: https://creativecommons.org/licenses/by/3.0/deed.en |

The following are the responses included in the feedback for students at the conclusion of each section of the CBRN Patient Worksheet and their corresponding Check on Knowledge questions (Appendix G). Correct responses are in red text.

Part I Feedback

**2. What was the first immediate instruction given to the patient?**

- Don a mask & treat self with antidote

**3. Name one additional action you would take or recommend as a medical leader? (Any of the following are acceptable responses.)**

- MOPP alert to unit and command/advise unit to don appropriate protective gear
- Remove patient from exposure area after donning PPE
- Evacuate additional unit members
- Prepare for additional casualties & communicate to other medical assets
- Prepare to establish casualty collection point, decontamination, triage, etc.
- 9-line/MEDEVAC request

Part II Feedback

**4. Using MARCHE(2), what was the immediate action/instruction provided by the corpsman in the video responding to this patient (mark only one option)?**

- Administering ATNAA/CANA
- Evacuating from site
- Securing the chemical mask
- Securing the chemical suit

**5. According to the GTA 03-08-002 JAN 2017, Contaminated Casualty Care, which statement(s) are TRUE for initial wound decontamination (select all that apply)?**

- During thorough patient decontamination, all bandages suspected of contamination are removed and the wounds are flushed with isotonic saline solution or water.
- Bandages are replaced only if bleeding begins after decontamination.
- Both bandage replacement and tourniquet replacement are performed by medical personnel in the warm zone before transferring to the cold zone.
- Tourniquets suspected of being contaminated are replaced with clean tourniquets, and the sites of the original tourniquets are decontaminated.
- Splints are thoroughly decontaminated but removed only by a medic or under a physician’s supervision.

Part III Feedback

**6. What was the physician's immediate action upon the patient's arrival to the aid station (mark only one option)?**

- Decontamination of the patient
- Immediate call for an NPA and Suction
- Primary Assessment
- Secondary Assessment

**7. According to the Multi-service Tactics, Techniques, and Procedures for Health Service Support in a Chemical, Biological, Radiological and Nuclear Environment, which statement(s) below are TRUE for Reactive Skin Decontamination Lotion (RSDL) (select all that apply)?**

- The RSDL can be used for the decontamination of intact skin around wounds, but is not approved for the decontamination of open wounds.
- RSDL is safe to be left on the skin for up to 24 hours.
- RSDL should remain in contact with the skin for at least two minutes and then be removed with soap and water when conditions permit.
- Basis of allocation of RSDL is two packets per individual (incorrect, as the allocation is one packet per individual).

**8. Provide two evacuation considerations for this patient. (Any of the following are acceptable responses.)**

- Ensure completion of casualty care card, to include communicating antidote administration & times delivered during patient handoff
- Communicate necessity of respiratory support capabilities, non-ambulatory state, potential for seizures, possible enemy presence in area, active CBRN threat, etc. in 9-line/MEDEVAC request
- Continue reassessment
- Ensure patient’s body temperature is maintained
- Evacuation from upwind location to avoid contamination
- Need for additional CBRN supplies for patient management
