# Supplementary material for: Introduction to Treating Patients Exposed to Chemical, Biological, Radiological, and Nuclear (CBRN) Threats: A Military Medical Case-Based Curriculum
Source: MedEdPORTAL. 2024 Sep 13;20:11433. doi: 10.15766/mep_2374-8265.11433 (PMC11393073; doi:10.15766/mep_2374-8265.11433)
Supplement: Supplementary file 1 — Session One Lecture.pptxSupplemental Resources for Session One.docxCBRN Patient Worksheet.docxPatient Worksheet Video - Introduction to CBRN Patient.mp4Patient Worksheet Video - CBRN Corpsman Response.mp4Patient Worksheet Video - Physician Assessment.mp4Check on Knowledge Form.docxCBRN Patient Worksheet - Facilitator Version.docxFacilitator Guide.docxStudent Survey.docxSupplemental Resources for Session Two.docx [file mep_2374-8265.11433-s001.zip › J. Student Survey.docx]

**Appendix J. Student Survey**

The following Student Survey was developed using the Google Forms platform and integrated with the Check on Knowledge Form (Appendix G) to facilitate completion. Students completed the survey as a team (one survey per team) at the conclusion of the worksheet.

This is a formative evaluation of the “CBRN Patient Case Study” lesson. The formative evaluation is the collection of data and information during the development of instruction that can be used to improve the effectiveness of instruction. Responses are confidential and will be used for instructional evaluation and lesson improvement.

**Clarity of Instruction**

The following 3 questions evaluate whether the message, or what is being presented, is clear to the individual target learners.

1. Were the instructional materials clearly understood (mark only one option)?
   - Extremely Understandable
   - Quite Understandable
   - Moderately Understandable
   - Slightly Understandable
   - Not at all Understandable
2. Did you find the scenario an appropriate context for this lesson (mark only one option)?
   - Extremely Appropriate
   - Quite Appropriate
   - Moderately Appropriate
   - Slightly Appropriate
   - Not at all Appropriate
3. How would you rate the sequence of instruction from pre-lesson activities to the end of the lesson (mark only one option)?
   - Seamless
   - Quite Connected
   - Moderately Connected
   - Slightly Connected
   - Not at all Connected

**Impact on Learner**

The following 3 questions evaluates the impact of the instruction on individual learner's attitudes and achievement of the goal and objectives.

1. Do you find the materials relevant to your needs and interests (mark only one option)?
   - Very Relevant
   - Quite Relevant
   - Moderately Relevant
   - Slightly Relevant
   - Not at all Relevant
2. How easy or difficult did you find the information or skills to learn (mark only one option)?
   - Very Easy
   - Somewhat Easy
   - Neither Easy nor Difficult
   - Somewhat Difficult
   - Very Difficult
3. How satisfied are you with what you have learned (mark only one option)?
   - Extremely Satisfied
   - Quite Satisfied
   - Moderately Satisfied
   - Slightly Satisfied
   - Not at all Satisfied

**Feasibility**

The following 2 questions evaluates the feasibility of the instruction given the available resources, i.e., time and context.

1. How would you assess your motivation as you progressed through this lesson (mark only one option)?
   - Extremely Motivated
   - Quite Motivated
   - Moderately Motivated
   - Slightly Motivated
   - Not at all Motivated
2. How would you assess the amount of time you had to complete the lesson (mark only one option)?
   - 1 – Not at all adequate
   - 2
   - 3
   - 4
   - 5 – More than adequate

**General**

1. Provide any additional comments.
