# Supplementary material for: Introduction to Treating Patients Exposed to Chemical, Biological, Radiological, and Nuclear (CBRN) Threats: A Military Medical Case-Based Curriculum
Source: MedEdPORTAL. 2024 Sep 13;20:11433. doi: 10.15766/mep_2374-8265.11433 (PMC11393073; doi:10.15766/mep_2374-8265.11433)
Supplement: Supplementary file 1 — Session One Lecture.pptxSupplemental Resources for Session One.docxCBRN Patient Worksheet.docxPatient Worksheet Video - Introduction to CBRN Patient.mp4Patient Worksheet Video - CBRN Corpsman Response.mp4Patient Worksheet Video - Physician Assessment.mp4Check on Knowledge Form.docxCBRN Patient Worksheet - Facilitator Version.docxFacilitator Guide.docxStudent Survey.docxSupplemental Resources for Session Two.docx [file mep_2374-8265.11433-s001.zip › K. Supplemental Resources for Session Two.docx]

**Appendix K. Optional Supplemental Resources for Session Two**

1. Ciottone GR. Toxidrome Recognition in Chemical-Weapons Attacks. N Engl J Med. 2018;378(17):1611-1620. doi:10.1056/NEJMra1705224
2. DeFeo DR, Givens ML. Integrating Chemical Biological, Radiologic, and Nuclear (CBRN) Protocols Into TCCC Introduction of a Conceptual Model - TCCC + CBRN = (MARCHE)2. J Spec Oper Med. 2018;18(1):118-123. doi:10.55460/ZK2U-M1DZ
3. Henretig FM, Kirk MA, McKay CA Jr. Hazardous Chemical Emergencies and Poisonings. N Engl J Med. 2019;380(17):1638-1655. doi:10.1056/NEJMra1504690
