# Supplementary material for: Introduction to Treating Patients Exposed to Chemical, Biological, Radiological, and Nuclear (CBRN) Threats: A Military Medical Case-Based Curriculum
Source: MedEdPORTAL. 2024 Sep 13;20:11433. doi: 10.15766/mep_2374-8265.11433 (PMC11393073; doi:10.15766/mep_2374-8265.11433)
Supplement: Supplementary file 1 — Session One Lecture.pptxSupplemental Resources for Session One.docxCBRN Patient Worksheet.docxPatient Worksheet Video - Introduction to CBRN Patient.mp4Patient Worksheet Video - CBRN Corpsman Response.mp4Patient Worksheet Video - Physician Assessment.mp4Check on Knowledge Form.docxCBRN Patient Worksheet - Facilitator Version.docxFacilitator Guide.docxStudent Survey.docxSupplemental Resources for Session Two.docx [file mep_2374-8265.11433-s001.zip › I. Facilitator Guide.docx]

**Appendix I. Introduction to Treating Patients Exposed to Chemical, Biological, Radiological and Nuclear Threats (CBRN): Facilitator’s Guide**

This facilitator’s guide provides a general background on each element of the sessions. This guide pairs with the facilitator’s version of the CBRN Patient Worksheet (Appendix H), which also includes answers to the Check on Knowledge Form (Appendix G). At the bottom of this document, there are suggestions for how this curriculum may be adapted for Emergency Medical Services (EMS) personnel.

**Session One**

*Materials Needed*

- Lecture hall with projector and microphone capabilities, if needed
- Optional virtual screening platform if providing students with a remote, synchronous viewing option

*Optional Pre-Work for Participants (2 hours)*

- Emergency Preparedness and Response Course: several versions of CBRN response courses are available through the CDC, FEMA, and, for military personnel, Joint Knowledge Online
- Lecture slides available for preview
- Optional reading available in Appendix B
- Reflection (no submission required): Look at the material through the lens of a leader, a clinician caring for a single patient or multiple patients, and as an individual who needs to protect themselves. Try to come up with some questions as you ponder those roles for the discussion at the end of the lecture.

*Lecture: An Introduction to Medical Care of the CBRN Patient (2 hours)*

- Lecture format: available as a live, in-person lecture with option for online/remote viewing
- Speaker notes are provided in the lecture slides (Appendix A)
- It is recommended that an instructor with an emergency medicine or toxicology (preferred) background provide the introductory lecture

*Post-Activity Reflection and Required Reading (1 hour)*

- Students are required to submit a written reflection within 3 days of Session One, addressing the following question: How are the constructs of Tactical Combat Casualty Care (TCCC) (or civilian prehospital trauma care) and the approach to CBRN similar and dissimilar, and why is it important to have a common framework for these topics?
  - Their reflection is graded for completion on a pass/fail basis
- Required Reading: DeFeo DR, Givens ML. Integrating Chemical Biological, Radiologic, and Nuclear (CBRN) Protocols Into TCCC. Introduction of a Conceptual Model - TCCC + CBRN = (MARCHE)2. J Spec Oper Med. 2018;18(1):118-123. doi:10.55460/ZK2U-M1DZ

**Session Two**

*Materials Needed*

- Lecture hall with microphone capabilities for faculty debrief at conclusion of the session
- Students must bring their own computers to access the small groups materials
- Large meeting space or multiple small rooms with sufficient tables for each student group to gather around
- One ChemBio (CB) Respirator (or equivalent per your institution/organization/EMS system) per team of students (provide cleaning supplies to wipe down respirators before and after use)

*Optional Pre-Work for Participants (1 Hour)*

- Review of the Joint Trauma System CBRN Injury clinical practice guidelines (or civilian equivalent):
  - Defense Health Agency. Joint Trauma System Clinical Practice Guideline (CPG) Chemical, Biological Radiological, and Nuclear (CBRN) Injury. Part I: Initial Response to CBRN Agents. May 2018. https://jts.health.mil/assets/docs/cpgs/Chemical_Biological,_Radiological_Nuclear_Injury_Part1_Initial_Response_01_May_2018_ID69.pdf. Accessed October 14, 2023.
  - Defense Health Agency. Joint Trauma System Clinical Practice Guideline (CPG) Chemical, Biological Radiological, and Nuclear (CBRN) Injury. Part II Chemical, Biological, Radiological and Nuclear (CBRN) Injury Response Part 2: Medical Management of Chemical Agent Exposure. January 2019. https://jts.health.mil/assets/docs/cpgs/Chemical_Biological_Radiological_Nuclear_Injury_Response_Part_2_Medical_Management_25_Mar_2022_ID69.pdf. Accessed October 14, 2023.
- Optional readings available in Appendix K
- Reflection (no submission required): Look at the material through the lens of a leader, a clinician caring for a single patient or few patients, and as an individual who needs to protect themselves.

*Resources and Recommendations for Facilitator Preparation*

- We highly recommend reviewing institutional/local/regional guidelines on CBRN response to include but not limited to the agencies involved, equipment availability, and clinical practice guidelines
- For a general overview of chemical agents and toxidrome recognition, please see the following reference (also listed in Appendix K for student review): Henretig FM, Kirk MA, McKay CA Jr. Hazardous Chemical Emergencies and Poisonings. N Engl J Med. 2019;380(17):1638-1655. doi:10.1056/NEJMra1504690
- For more information on the use and administration of atropine, please see the following reference from the FDA: 1. Use of the Rafa atropine auto-injector. U.S. Food and Drug Administration. January 2018. https://www.fda.gov/media/104559/download. Accessed February 23, 2024.

*Small Groups: CBRN Patient Worksheet (Appendix C; 1-1.5 Hours)*

- The worksheet (Appendix C) is a student-driven exercise, with each team working independently at their table
- Emphasize that students should proceed in order through the worksheet, without skipping sections
- All team members should work together as they advance through the worksheet (e.g., teams should not approach the activity with a “divide and conquer” plan, splitting the worksheet amongst its members)
- Allow for 60-90 minutes to complete the worksheet
- Facilitators should be available, moving about the room, to address any clarifying questions by referencing to the Facilitator’s version of the CBRN Patient Worksheet (Appendix H) and to engage in discussion
  - The facilitator can choose to review/debrief the videos with the students as a larger group as they work through the worksheet
- Facilitators may assist by providing time hacks throughout the activity to ensure students are progressing at an appropriate pace
- It may be helpful to ask students to contribute to the Student Survey (Appendix J) as a team
- CB Respirator: Per the worksheet, each team will designate one student to act as an unconscious patient while the other team members work to apply the CB respirator to the unconscious patient. Emphasis should be placed on achieving a good seal (tightening the mask as much as possible) and the fact that the unconscious patient will likely be unable to clear their mask (thus, some degree of contamination likely persists, but is diminished).
  - Please reference local guidelines and the instructions for the specific respirator available to your students/personnel (if any)

*Faculty Debrief (30 mins)*

- Discuss CRESS, toxidromes, and the importance of including CBRN agents early in the differential diagnosis to allow for rapid decontamination
- Consider asking students the following questions:
  - How do the principles of CBRN overlap with daily medical practice?
  - How will you approach your clinical practice to keep CBRN fresh in your mind and be diligent in surveillance?
- It is recommended that an instructor with a toxicology or emergency medicine background provide the debrief

*Required Post-Exercise Submissions (5 mins)*

- Participants must submit the student survey immediately after completing the CBRN worksheet
- Participants must send one copy of the CBRN worksheet per team so that they may be graded for completion (pass/fail)

**Adapting this Curriculum for EMS** **Personnel**

In considering how to adapt this curriculum for different audiences, one should interrogate scope of practice, level of training, equipment availability, and healthcare roles during CBRN events. Please also reference your local health department, EMS, hospital, and regional poison control center guidelines. We have listed suggestions for adapting this curriculum for EMS personnel, with significant expert contributions from Melissa Givens, MD, MPH.

- Discuss available CBRN personal protective gear (e.g., what type of masks are available) and how the gear can be accessed
- Consider allotting time for personnel to practice donning and doffing CBRN personal protective gear
- Cross-train with specialized all-hazard response teams
- Actual curricular content, such as the Session One Lecture slides, does not need to be adjusted extensively for pre-hospital personnel; military medics follow the clinical practice guidelines shared in the slides and participate in CBRN training exercises
- Consider hosting a CBRN mass casualty exercise for skills implementation
- Discuss similarities and differences between EMS specific treatment algorithms and the (MARCHE)2 algorithm
- Provide EMS reference textbooks or online resources that personnel may refer to, when needed
- Create or print algorithm cards for addressing CBRN threats (similar to clinical practice guidelines)
- Consider differences/similarities in deliberate CBRN events versus occupational exposures/accidents
- The activity worksheet is specific to one category of agent; practice with a variety of agents across the categories is recommended
