# Supplementary material for: Introduction to Treating Patients Exposed to Chemical, Biological, Radiological, and Nuclear (CBRN) Threats: A Military Medical Case-Based Curriculum
Source: MedEdPORTAL. 2024 Sep 13;20:11433. doi: 10.15766/mep_2374-8265.11433 (PMC11393073; doi:10.15766/mep_2374-8265.11433)
Supplement: Supplementary file 1 — Session One Lecture.pptxSupplemental Resources for Session One.docxCBRN Patient Worksheet.docxPatient Worksheet Video - Introduction to CBRN Patient.mp4Patient Worksheet Video - CBRN Corpsman Response.mp4Patient Worksheet Video - Physician Assessment.mp4Check on Knowledge Form.docxCBRN Patient Worksheet - Facilitator Version.docxFacilitator Guide.docxStudent Survey.docxSupplemental Resources for Session Two.docx [file mep_2374-8265.11433-s001.zip › B. Supplemental Resources for Session One.docx]

**Appendix B. Optional Supplemental Resources for Session One**

1. Defense Health Agency. Joint Trauma System Clinical Practice Guideline (CPG) Chemical, Biological Radiological, and Nuclear (CBRN) Injury. Part I: Initial Response to CBRN Agents. May 2018. https://jts.health.mil/assets/docs/cpgs/Chemical_Biological,_Radiological_Nuclear_Injury_Part1_Initial_Response_01_May_2018_ID69.pdf. Accessed October 14, 2023.
2. Defense Health Agency. Joint Trauma System Clinical Practice Guideline (CPG) Chemical, Biological Radiological, and Nuclear (CBRN) Injury. Part II Chemical, Biological, Radiological and Nuclear (CBRN) Injury Response Part 2: Medical Management of Chemical Agent Exposure. January 2019. https://jts.health.mil/assets/docs/cpgs/Chemical_Biological_Radiological_Nuclear_Injury_Response_Part_2_Medical_Management_25_Mar_2022_ID69.pdf. Accessed October 14, 2023.
3. Department of Defense. Mission Oriented Protective Posture (MOPP). August 15, 2011. https://media.defense.gov/2012/Jan/13/2000186472/1200/1200/0/120113-F-SU363-001.JPG. Accessed October 14, 2023.
4. Jones SL, Walsh RS, Stearney SA, Allen R. Multi-service Tactics, Techniques, and Procedures for Health Service Support in a Chemical, Biological, Radiological and Nuclear Environment. Army Publishing Directorate. March 2016. https://armypubs.army.mil/epubs/DR_pubs/DR_a/pdf/web/atp4_02x7.pdf. Accessed October 14, 2023.
5. NIAID Biodefense Pathogens. National Institute of Allergy and Infectious Diseases. NIAID Emerging Infectious Diseases/Pathogens. June 13, 2024. https://www.niaid.nih.gov/research/niaid-biodefense-pathogens. Accessed August 22, 2024.
6. United States Army Combined Arms Center. GTA 03-08-002 Contaminated Casualty Care. January 23, 2017. https://usacac.army.mil/organizations/mccoe/call/publication/GTA_03-08-002. Accessed October 14, 2023.
7. US Army Medical Research Institute of Infectious Diseases. USAMRIID Research. July 28, 2023. https://usamriid.health.mil/index.cfm/research/overview. Accessed October 14, 2023.
